# A prognostic model based on autophagy-and senescence-related genes for gastric cancer: implications for immunotherapy and personalized treatment

**DOI:** 10.3389/fonc.2025.1509771

**Published:** 2025-03-20

**Authors:** Shuming Chen, Xiaoxi Han, Yangyang Lu, Shasha Wang, Yuanyuan Fang, Chuanyu Leng, Xueying Sun, Xin Li, Wensheng Qiu, Weiwei Qi

**Affiliations:** Department of Oncology, The Affiliated Hospital of Qingdao University, Qingdao, China

**Keywords:** autophagy, senescence, immunotherapy, TXNIP, gastric cancer

## Abstract

**Background:**

The process of human aging is accompanied by an increased susceptibility to various cancers, including gastric cancer. This heightened susceptibility is linked to the shared molecular characteristics between aging and tumorigenesis. Autophagy is considered a critical mediator connecting aging and cancer, exerting a dynamic regulatory effect in conjunction with cellular senescence during tumor progression. In this study, a combined analysis of autophagy- and senescence-related genes was employed to comprehensively capture tumor heterogeneity.

**Methods:**

The gene expression profiles and clinical data for GC samples were acquired from TCGA and GEO databases. Differentially expressed autophagy- and senescence-related genes (DEASRGs) were identified between tumor and normal tissues. Gene Ontology (GO) and Kyoto Encyclopedia of Genes and Genomes (KEGG) pathway analyses were carried out to provide insights into biological significance. A prognostic signature was established using univariate Cox and LASSO regression analyses. Furthermore, consensus clustering analyses and nomograms were employed for survival prediction. TME and drug sensitivity analyses were conducted to compare differences between the groups. To predict immunotherapy efficacy, the correlations between risk score and immune checkpoints, MSI, TMB, and TIDE scores were investigated.

**Results:**

A fourteen-gene prognostic signature with superior accuracy was constructed. GC patients were stratified into three distinct clusters, each exhibiting significant variations in their prognosis and immune microenvironments. Drug sensitivity analysis revealed that the low-risk group demonstrated greater responsiveness to several commonly used chemotherapeutic agents for gastric cancer, including oxaliplatin. TME analysis further indicated that the high-risk group exhibited increased immune cell infiltration, upregulated expression of ICs, and a higher stromal score, suggesting a greater capacity for immune evasion. In contrast, the low-risk group was characterized by a higher proportion of microsatellite instability-high (MSI-H) cases, an elevated TIDE score, and a greater TMB, indicating a higher likelihood of benefiting from immunotherapy. In addition, Single-cell sequencing demonstrated that TXNIP was expressed in epithelial cells. Cellular experiments preliminarily verified that TXNIP could promote the proliferation and migration of gastric cancer cells.

**Conclusion:**

This study presents a robust predictive model for GC prognosis using autophagy- and senescence-related genes, demonstrating its ability to predict immune infiltration, immunotherapy effectiveness, and guide personalized treatment.

## 1 Introduction

Globally, gastric cancer remains the second leading cause of cancer-related death and the fourth most common cancer (1). Despite a high incidence and mortality rate, the prevalence of GC varies significantly across different regions and individuals (1, 2), indicating its substantial heterogeneity (3). While recent advancements in GC diagnosis and treatment have been considerable, the traditional prognostic system based on tumor stage and histological grade is increasingly inadequate for capturing the observed clinical heterogeneity (4). In the era of precision medicine, developing novel diagnostic and prognostic models based on patients’ molecular signatures and clinical characteristics holds significant promise.

Cancer, including gastric cancer, is well acknowledged as a disease associated with ageing. As individuals age, chronic inflammation, and the accumulation of senescent cells collectively contribute to an environment conducive to cancer formation (5). At the cellular level, senescence is characterized by the irreversible arrest of cell proliferation in response to cellular stress (6). During the initial phases of carcinogenesis, cellular senescence is frequently regarded as a protective mechanism that prevents the proliferation of potentially malignant cells. Senescent cells, however, secrete the senescence-associated secretory phenotype (SASP), which comprises a variety of pro-inflammatory cytokines and chemokines. These secretory components enhance the malignant characteristics of tumor cells and accelerate their immune evasion mechanisms (7, 8). Therefore, comprehending the dual function of senescence and the complex interactions between senescent cells and tumor cells is essential for the formulation of innovative anti-cancer strategies.

Autophagy is recognized as a critical link between aging and cancer (9). It is a highly conserved cellular catabolic process that facilitates the recycling of cellular components through lysosomal degradation (10). During the initial phases of tumorigenesis, autophagy acts as a tumor-suppressive mechanism by eliminating damaged organelles, preserving genomic stability, and promoting cellular senescence (11). In contrast, in advanced tumors, autophagy aids in the survival of senescent cells through metabolic reprogramming. Concurrently, the SASP is activated, releasing pro-inflammatory factors such as IL-6 and TGF-β, which modify the tumor microenvironment (TME), promoting immune evasion and facilitating metastasis (8). However, senescence-related signals can also influence autophagic activity through a feedback loop. Consequently, autophagy and cellular senescence engage in a dynamic, bidirectional regulatory relationship during tumor progression.

In summary, the present study sought to identify a gene signature incorporating autophagy and senescence factors to accurately predict the prognosis of GC. A fourteen-gene signature was constructed using univariate Cox regression and LASSO regression. Additionally, the predictive performance of the model was further enhanced through the establishment of a nomogram. A detailed analysis was performed on gastric cancer subtypes, immune cell infiltration, the distribution of ICs, gene mutation differences, and drug sensitivity differences in the TCGA cohort. In addition, cellular function experiments preliminarily verified the role of TXNIP in gastric cancer. Collectively, this research has the potential to uncover novel characteristic genes that serve as reliable prognostic biomarkers for the personalized treatment of GC patients.

## 2 Materials and methods

### 2.1 Data sources

The raw data was downloaded from TCGA and GEO databases. Duplicate samples and those lacking essential clinical characteristics or survival information were removed. The training cohort consisted of 410 STAD samples and 10 gastric normal samples from TCGA. The validation cohort was selected to be the GSE66229 dataset (12), which was verified using the GPL570 platform. To eliminate discrepancies caused by batch effects and ensure research integrity and reliability, COMBAT was used when merging GEO data. In the survival analyses, patients were included based on the availability of survival status and survival time, with a minimum survival time of 30 days. The list of genes associated with autophagy and senescence was obtained from GeneCards datasets (13).

### 2.2 Analysis of differentially expressed genes

To identify common genes, Venny 2.1.0 was employed. DEGs were detected using the “limma” package in R. A cutoff of |logFC| ≥ 1 and a FDR < 0.05 were applied to determine significant DEGs. A volcano plot was drawn by the “pheatmap” package in R to visually represent the DEGs.

### 2.3 Functional annotation and enrichment analyses

The “clusterProfiler” package was employed to conduct GO and KEGG analyses. These analyses identified the biological functions and pathways associated with the DEASRGs, providing insights into their biological significance. Furthermore, the functional profiles of the different risk groups were assessed using Gene Set Enrichment Analysis (GSEA) to detect any relevant changes.

### 2.4 Consensus clustering to identify DEASRG clusters

DEASRGs were utilized to conduct consensus cluster analysis using the “ConsensusClusterPlus” package in R. Employing optimal k-means clustering, STAD patients were categorized into three distinct groups. Principal component analysis (PCA) was implemented to differentiate these clusters. We used the “estimate” package to determine particular scores in tumor tissue for assessing the extent of infiltration by stroma and immune cells.

### 2.5 Construction and verification of autophagy- and senescence-related risk score signature

Univariate Cox analysis was employed to screen core prognostic DEASRGs and we further assessed their copy number variation (CNV) alterations. Subsequently, LASSO regression was utilized to select genes for constructing the prognostic signature. Through the calculation of the following algorithm, we obtained corresponding risk score for every single patient.


$$Risk\;score=\sum_{\text{i}=1}^{\text{n}}\left(\text{Coefi}*\text{Expi}\right)$$

The variables n, Coefi, and Expi represent the signature gene number, the risk weighting coefficient index, and the expression level of the signature gene, respectively.

The median risk score in the TCGA cohort was used to distinguish the high-risk group from the low-risk group. KM survival curves were generated to compare prognosis between the groups. We constructed a ROC curve to compare the Area Under the Curve (AUC) value of the risk score and several clinical markers. Risk curves and survival status analyses were performed to evaluate the efficiency of model in high- and low-risk groups. Additionally, we conducted PCA analysis to visualize the distribution of patients.

### 2.6 The modification of predictive signature-nomogram

To enhance predictive power the signature, a nomogram was established incorporating risk scores and clinical features. Variables within the nomogram, including age, gender, M stage, T stage, N stage, clinical stage, and risk score, were assigned points based on their relative prognostic contributions. Individual patient scores were calculated by summing these points. The calibration curve allowed us to evaluate the predictive capability of the nomogram across various survival periods. Decision curve analysis (DCA) was used to evaluate the clinical benefits brought by the model.

### 2.7 Stratified analysis of clinicopathological features

To investigate the association between the novel signature and various clinical factors, subgroup analyses were conducted within the TCGA cohort. Available clinicopathological features were extracted for further analysis. Moreover, survival curves were also plotted across distinct clinical subgroups.

### 2.8 Screening of sensitive drugs

Drug sensitivity assessments was performed using data obtained from the Genomics of Drug Sensitivity in Cancer (GDSC) public database (14). The “oncoPredict” package was employed to calculate the half-maximal inhibitory concentration (IC_50_) values for therapeutic drug.

### 2.9 Immune cell infiltration and immunotherapy response

The CIBERSORT algorithm enabled us to accurately assess the composition of infiltrating immune cell types within patient’s tumor sample (15). To assess potential treatment response based on proportions of immune cell, we examined the expression of immune checkpoint genes within the two subgroups. Furthermore, we employed the tumor immune dysfunction and exclusion (TIDE) algorithm to obtain TIDE scores, dysfunction scores, and exclusion scores for each tumor sample. Tumor purity was evaluated using the ESTIMATE algorithm. The stromal score represents the proportion of stromal cells, while the immune score reflects the balance of immune cell populations. Finally, we visualized the distribution differences between risk groups and microsatellite status (MSI) states using boxplots.

### 2.10 Mutation analysis

We downloaded somatic mutation data of training group via the UCSC Xena browser. This data was then visualized using waterfall charts from the “maftools” R package. Next, we computed the tumor mutational burden (TMB) score for every single sample and investigated its correlation with risk score.

### 2.11 Visualization of protein-protein interaction networks

Associations between these model genes were constructed by STRING database. A PPI network was built using interaction scores higher than 0.15 and P < 0.05 as the significant threshold. Genes with interaction scores greater than 0.15 were selected to construct PPI network. Cytohubba plugin was utilized to estimate the MCC score, Stress score, Degree score and Closeness score. In this analysis, genes with the same score were considered to be sequenced equally.

### 2.12 Single-cell data analysis

The raw expression profiling of GSE112302 was retrieved from the GEO dataset. The data pertaining to normal tissue were omitted, while the data corresponding to tumor tissue were utilized for subsequent analysis. The employment of the “Seurat” program was necessary for performing data quality control, PCA, and t-Distributed Stochastic Neighbor Embedding (t-SNE) visualization are all reliant on the utilization of the “Seurat” package. The “SingleR” package was vital for annotating the cell types in each cluster.

### 2.13 Cell culture

The gastric cancer cell lines (SGC7901, AGS, HGC27) and the human normal gastric mucosal cell line GES-1 were procured from Pricella Life Science & Technology Co., Ltd. These cell lines were cultured in RPMI-1640 medium (Pricella Life Science & Technology Co., Ltd) supplemented with 10% Fetal Bovine Serum (FBS, from Shanghai Life-iLab Biotech Co., Ltd.), and containing penicillin and streptomycin.

### 2.14 Cell viability assay

The growth of AGS and HGC27 cells was assessed using the MTT assay. Cells were seeded at a stable density in a 24-well plate and incubated. Subsequently, 0.5 mg/mL MTT solution (M158055, Aladdin) was added to each well at 24, 72, and 120 hours, respectively. After 3-4 hours of incubation at 37°C, the supernatant was removed, and dimethyl sulfoxide was added to dissolve the formazan precipitate. The absorbance of the resulting solution was measured at 490 nm using a microplate spectrophotometer.

### 2.15 Colony formation assay

To assess the proliferation of AGS and HGC27 cells, a cell cloning assay was performed. Cells were seeded into a six-well plate and incubated under standard conditions for 10-14 days. Subsequently, the supernatant was removed, and cells were fixed with 4% paraformaldehyde (30072418, China National Pharmaceutical Group Chemical Reagent Co., Ltd). Following fixation, cells were stained with crystal violet, and the number of colonies formed was manually counted.

### 2.16 Migration assay

To assess the migration of AGS and HGC27 cells, a cell scratch assay was performed. Cells were seeded in a 24-well plate and incubated for 2-3 days until reaching approximately 90% confluency. A wound was created on the cell monolayer using a 200 µL pipette tip. Cell migration into the wound area was observed and evaluated after 48 hours.

### 2.17 Western blot

Use cell lysis buffer (containing 20mM Tris (pH 7.5), 150mM NaCl, 1% Triton X-100, and other components) to lyse cell samples. Protein concentration in the collected lysates was determined using the BCA quantification method. Subsequently, 25-40 µg of protein was separated on an SDS-PAGE gel, followed by transfer to a polyvinylidene fluoride (PVDF) membrane. The membrane was incubated overnight at 4°C with primary antibodies against TXNIP and GAPDH. Afterward, the membrane was incubated with a secondary antibody at room temperature for 2 hours. Protein bands were visualized using ECL chemiluminescence.

### 2.18 Statistical analysis

The work in this study was primarily performed by R software (version 4.3.1). Univariate and multivariate Cox regression analysis were employed to evaluate the independent prognostic significance of variables. For survival analysis, the Kaplan-Meier method was employed to plot the survival curves of different risk groups, and the Log-rank test was utilized to evaluate the significance of survival differences between groups. Regarding the comparison of continuous data, the independent Student’s t-test was carried out for normally distributed data between two groups, while the Wilcoxon test was applied for non - normally distributed continuous variables. The Spearman’s correlation coefficient was computed to evaluate the associations between two variables. Statistical significance was determined by setting the threshold at p < 0.05 for all analyses.

## 3 Results

### 3.1 Identification of autophagy- and senescence-related prognostic DEGs in STAD and functional enrichment analysis

**Figure 1:**
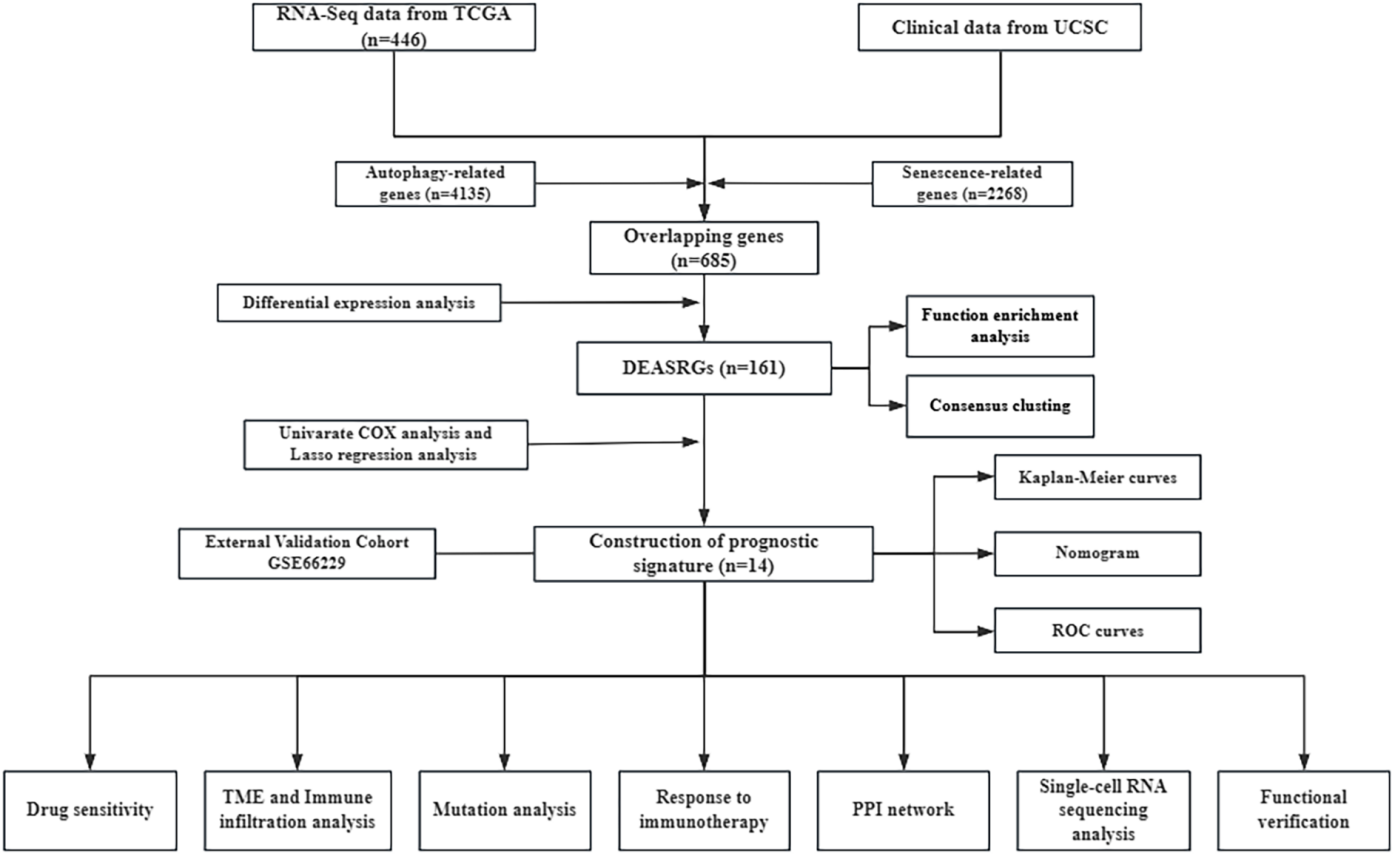
Study workflow.

**Figure 2:**
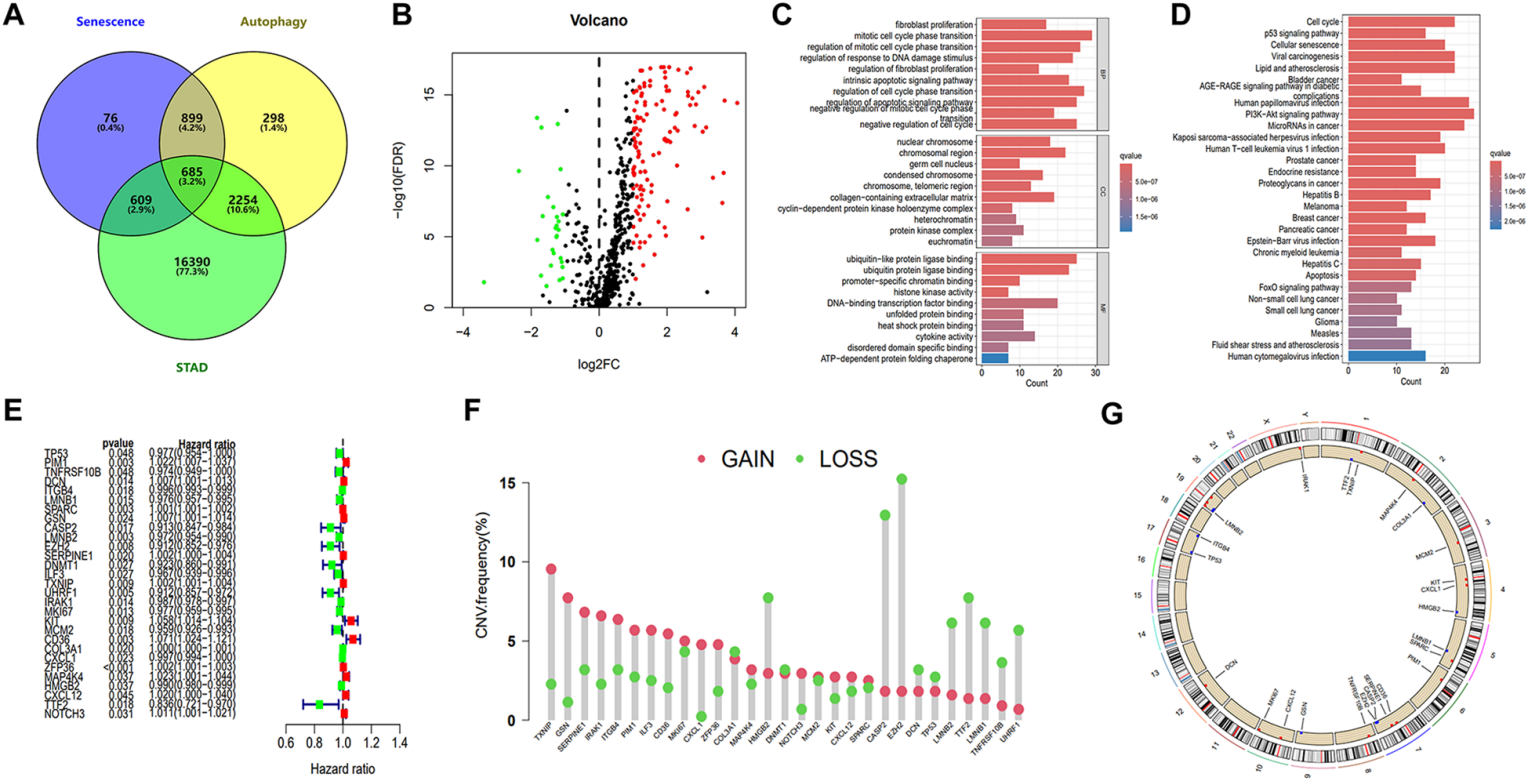
Identification of core prognostic genes and enrichment analysis. **(A)** Venn diagram illustrating the intersection of 685 genes associated with autophagy, senescence, and STAD. **(B)** Volcano plot of DEASRGs based on intersected genes. **(C)** GO functional annotation of DEASRGs. **(D)** KEGG enrichment analysis of DEASRGs. **(E)** Univariate Cox regression analysis identifying 29 genes. **(F)** Frequencies of CNV gain and loss among 29 prognostic genes. **(G)** Circular plots visualizing chromosome distributions of core prognostic genes.

Firstly, a general workflow was constructed to outline the entire analysis process (**Figure 1**). **Supplementary table 1** displayed a fundamental table containing baseline data of certain participants in this study. These participants have available survival status and survival time (≥30 days), and their T, N, M and clinical stage are clearly defined. As illustrated in the Venn plot (**Figure 2A**), 685 overlapping genes were obtained by intersecting 2269 autophagy-related genes and 4136 senescence-related genes from the GeneCards database with TCGA-STAD genes. Through differential expression analysis of these overlapping genes, 161 autophagy- and senescence-related DEGs (DEASRGs) were filtered comparing normal and tumor groups (**Figure 2B**). To provide a clearer understanding of the functional properties of DEASRGs in STAD, GO enrichment analysis was performed. The GO terms for biological processes and molecular functions revealed that the DEASRGs were mainly involved in the regulation of mitotic cell cycle phase transition, regulation of response to DNA damage stimulus, and ubiquitin-like protein ligase binding (**Figure 2C**). Additionally, KEGG pathway analysis was conducted to investigate the possible mechanistic pathways associated with these DEGs, including the cell cycle, cellular senescence, and PI3K-Akt signaling pathway (**Figure 2D**).

### 3.2 Construction and validation of the autophagy- and senescence-related signature

**Figure 3:**
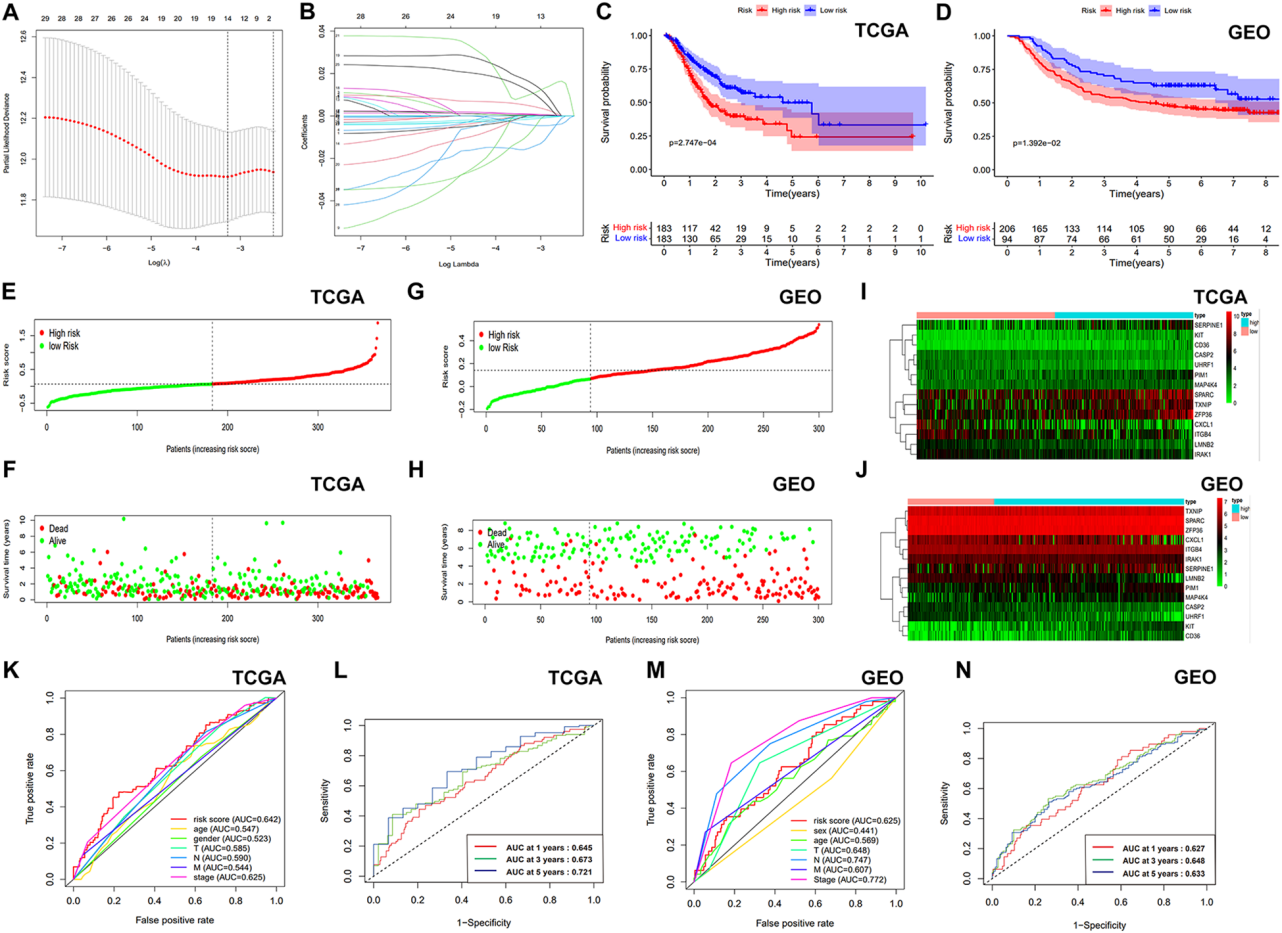
Development and verification the ASRGs signature. **(A)** LASSO regression model selection curve with log(λ) on the x-axis and partial likelihood deviance on the y-axis. **(B)** Coefficients of the LASSO regression model. **(C, D)** KM survival curves of OS. **(E, G)** Survival curves of patients with GC. **(F, H)** Distribution of survival status based on risk score. **(I, J)** Heatmaps of gene expression for the prognostic model genes. **(K, M)** Comparison of ROC curves. **(L, N)** ROC curve using temporal information (time-dependent ROC curves).

Through univariate Cox regression analysis, we identified 29 genes from the initial 161 DEASRGs as potential prognostic factors for STAD patients (**Figure 2E**). We investigated CNV alterations in these 29 genes, revealing predominantly copy number gains. However, COL3A1, HMGB2, DNMT1, CASP2, EZH2, DCN, TP53, LMNB2, TTF2, LMNB1, TNFRSF10B and UHRF1 exhibited a greater frequency of CNV losses (**Figure 2F**). The chromosomal location of CNV alterations for these 29 genes is depicted in **Figure 2G**. Finally, LASSO regression analysis further reduced the gene set to 14 (**Figures 3A, B**).

After calculating risk scores, patients were categorized into high- and low-risk groups using the median risk score as a threshold. KM survival analysis demonstrated a statistically better OS for the individuals classified as low risk (**Figures 3C, D**). The risk score exhibited a negative correlation with patient survival, indicating that a higher risk score predicts lower overall survival and higher mortality (**Figures 3E, F**). Similar analyses were conducted on the validation cohort (**Figures 3G, H**). **Figures 3I, J** presents gene expression profiles of the prognostic model genes as heatmaps. Our model demonstrated superior prognostic accuracy for gastric cancer patients compared to traditional clinical indicators in TCGA cohort (**Figure 3K**). Time-dependent ROC curves with AUCs of 0.645, 0.673, and 0.721 at 1, 3, and 5 years further validated the model’s efficiency (**Figure 3L**). In the GSE66229 dataset, our model outperformed most clinical indicators (**Figure 3M**), with AUCs of 0.627, 0.648, and 0.633 at 1, 3, and 5 years (**Figure 3N**). After that, we employed GSEA method to investigate disparities in biological functionality between patients classified as high and low risk (**Supplementary Figure S1**). Interestingly, the findings indicated that the biological functions of the high-risk group were intricately linked to the composition and specific activities of the extracellular matrix, while the low-risk group was primarily enriched in cell cycle, certain activities related to DNA and mitochondria. Univariate and multivariate Cox analyses confirmed the risk score as a significantly independent predictor of gastric cancer in the TCGA-STAD and GSE66229 datasets (**Supplementary Figures S2A-D**). Next, we examined the association between risk scores and clinical factors. The findings suggested that the younger populations (<65) had considerably higher risk scores (**Supplementary Figure S2E**). In addition, the differences in risk scores among gender, T stage, N stage, M stage, and clinical stage were not statistically significant (**Supplementary Figures S2F-J**). Moreover, **Supplementary Figure S3** illustrated the difference in survival rates among high- and low-risk patients across several clinical subgroups.

### 3.3 Identification of three subtypes by consensus clustering

**Figure 4:**
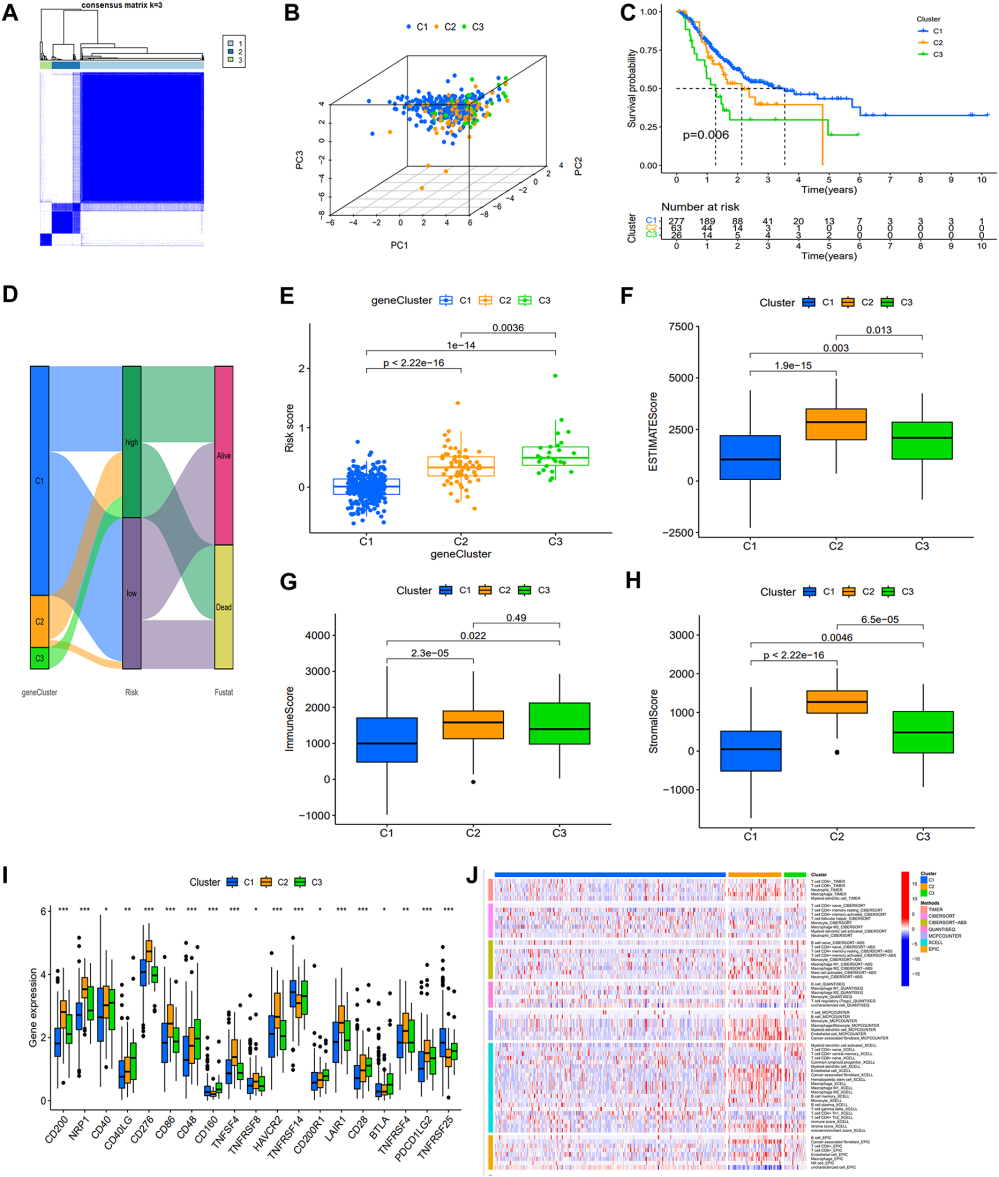
Association of the prognostic signature with gene clusters and immunological features. **(A)** The heat map display of consensus clustering is categorized into three cluster (C1 = 277; C2 = 63; C3 = 26). **(B)** PCA showing the perfect separation of C1, C2 and C3. **(C)** KM survival curves with three distinct clusters. **(D)** A Sankey diagram illustrating the link between gene clusters, risk group, and survival status. **(E)** Variations in risk score among the three gene subtypes. **(F-H)** ESTIMATE algorithm results for three gene clusters. **(I)** Expression of immune checkpoints related genes. **(J)** The heat map depicting variations in immune cell infiltration as determined using TIMER, CIERSORT, quanTIseq, MCPcounter, xCell, and EPIC algorithms.

Based on the expression of DEASRGs, we employed the Consensus Cluster algorithm to identify three distinct patient subtypes within the TCGA-STAD cohort, designated as clusters 1, 2, and 3 (**Figure 4A**). A PCA plot visualized the transcriptional differences among the three clusters in a three-dimensional space (**Figure 4B**). Survival analyses revealed a significant survival advantage for patients in cluster 1 (**Figure 4C**). A Sankey diagram illustrated patient transitions among gene clusters, risk groups, and survival status, demonstrating higher survival rates in cluster 1 and low-risk group (**Figure 4D**). The risk score in cluster 1 exhibited a statistically significant decrease compared to the other two clusters (**Figure 4E**). We subsequently assessed inter-cluster variations in the immunological microenvironment (**Figures 4F-H**). Notably, cluster 2 exhibited considerably higher expression levels of most ICs than the other clusters (**Figure 4I**), suggesting a suboptimal response to immunotherapy. A heatmap comparing immune cell infiltration patterns across clusters using algorithms from multiple platforms is presented in **Figure 4J**. C2 exhibited the highest overall immune cell infiltration, consistent with the findings from the ESTIMATE algorithm (**Figure 4G**). However, elevated expression of M2 macrophages, myeloid-derived suppressor cells, and tumor-associated fibroblasts was detected in C2 across multiple platforms, suggesting that this subgroup resides in an immunosuppressive microenvironment.

### 3.4 Establishing a predictive nomogram

**Figure 5:**
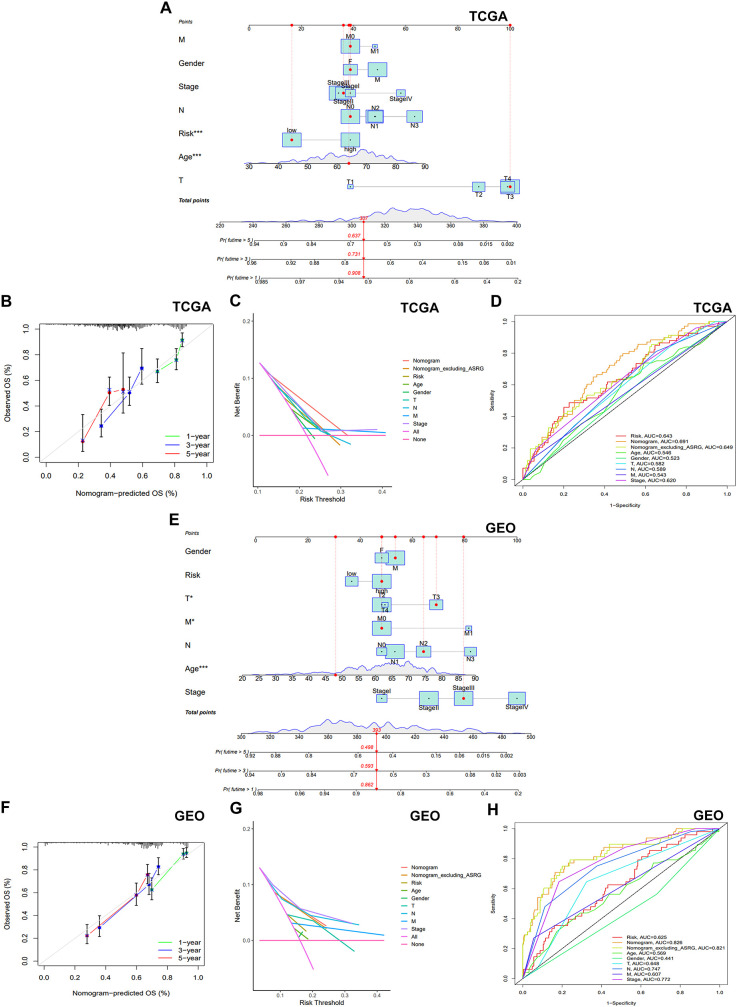
Establishment and validation of the nomogram. **(A, E)** A nomogram was established to forecast the 1-year, 3-year, and 5-year OS. **(B, F)** Calibration plots illustrating the agreement of predicted survival rates compared to the actual observed survival rates. **(C, G)** A DCA was carried out to compare the net benefits of the nomogram incorporating the prognostic signature, the nomogram excluding the prognostic signature, and other factors. **(D, H)** The AUC was employed to compare the predictive accuracy of the nomogram with other prognostic markers.

To enhance the clinical applicability and predictive accuracy of our signature, a nomogram was constructed incorporating risk score and other clinical indicators (**Figures 5A, E**). Calibration curves exhibited robust concordance between the expected and observed survival probability at 1, 3, and 5 years (**Figures 5B, F**), indicating high nomogram accuracy and reliability. DCA curve revealed that the nomogram exhibited larger net benefit compared to the nomogram without prognostic signature (nomogram excluding ASRG) and other factors (**Figures 5C, G**). ROC curve demonstrated superior predictive accuracy of the nomogram compared to other factors, such as nomogram excluding ASRG, risk score, gender, age, and TNM stage, with AUC values of 0.691 in the training set and 0.826 in the validation set (**Figures 5D, H**). The above results indicated that the incorporation of prognostic signature contributed to enhancing the superiority of the nomogram.

### 3.5 Relationship between ASRG signature and drug sensitivity

**Figure 6:**
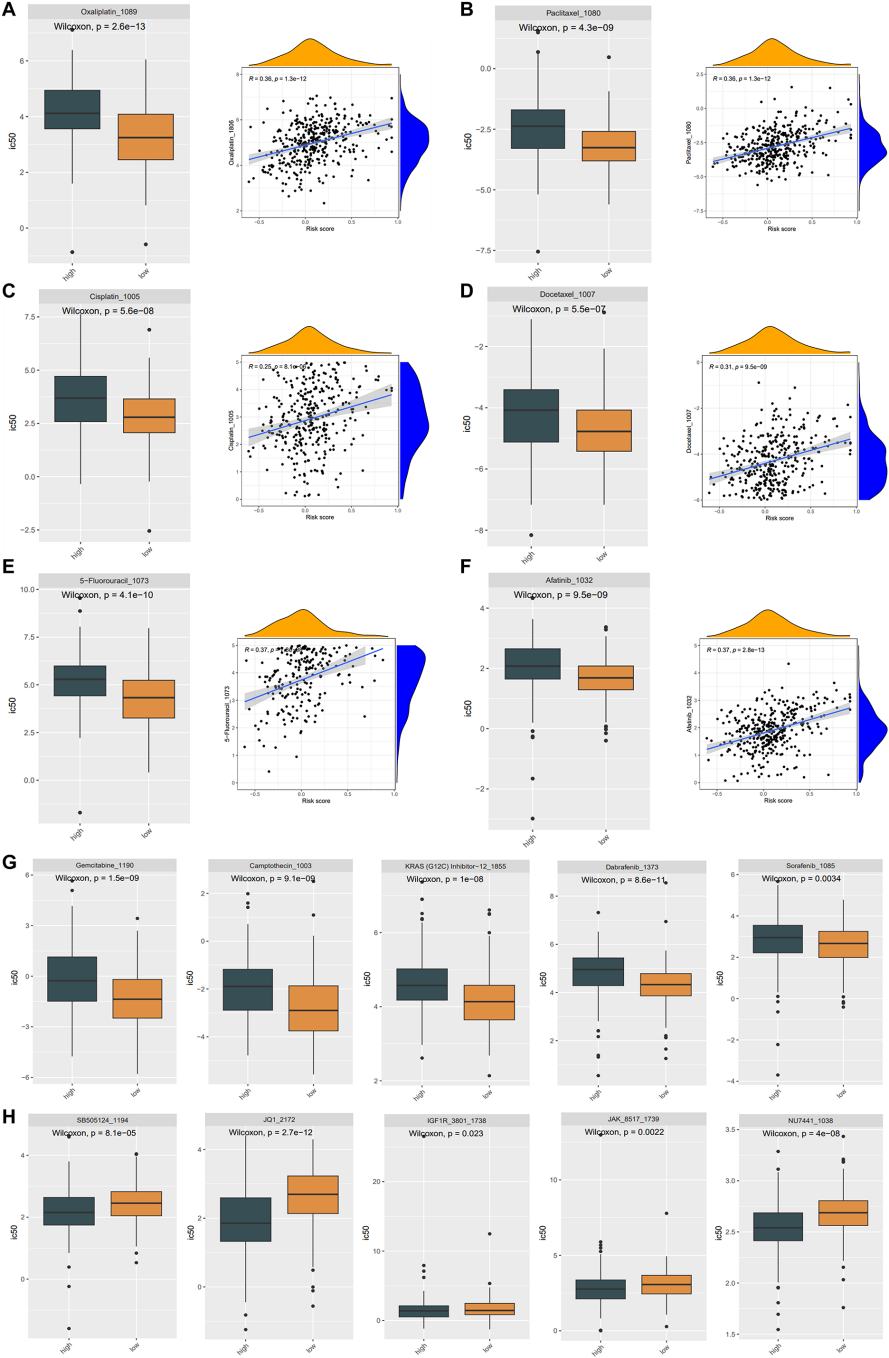
Drug sensitivity in the TCGA cohort. **(A-F)** The IC_50_ of Oxaliplatin **(A)**, Paclitaxel **(B)**, Cisplatin **(C)**, Docetaxel **(D)**, 5-Fluorouracil **(E)**, Afatinib **(F)** were considerably lower in the low-risk group, and there was a favorable correlation between the IC_50_ values of these drugs and the risk score. The difference in drug sensitivity showing the IC_50_ of Gemcitabine, Camptothecin, KRAS (G12C) Inhibitor, Dabrafenib, Sorafenib drugs were significantly higher in high-risk groups **(G)**, while the IC_50_ of SB505124, JQ1, IGF1R, JAK, NU7441 were significantly higher in low-risk groups **(H)**.

Resistance to therapeutic medications is a common challenge in cancer therapy, often leading to poor drug efficacy and worse clinical outcomes in gastric cancer. To enhance therapeutic benefits, we figured out whether the ASRG signature could accurately predict drug sensitivity in the training cohort. By utilizing the “oncoPredict” package, we estimated IC_50_ values for 198 drugs in all patients. The high-risk patients showed markedly elevated IC_50_ values for Oxaliplatin, Paclitaxel, Cisplatin, Docetaxel, 5-Fluorouracil, and Afatinib, which were positively correlated with risk scores. This suggested that individuals with lower risk scores might exhibit a more favorable response to therapies containing these medications (**Figures 6A-F**). Gemcitabine, Camptothecin, KRAS (G12C) Inhibitor, Dabrafenib, and Sorafenib also exhibited increased IC_50_ values in the high-risk group (**Figure 6G**). Conversely, SB505124, JQ1, IGF1R, JAK, and NU7441 had higher IC_50_ values in the low-risk patients, implying a poorer response to these drugs (**Figure 6H**).

### 3.6 Immunological features of the signature

**Figure 7:**
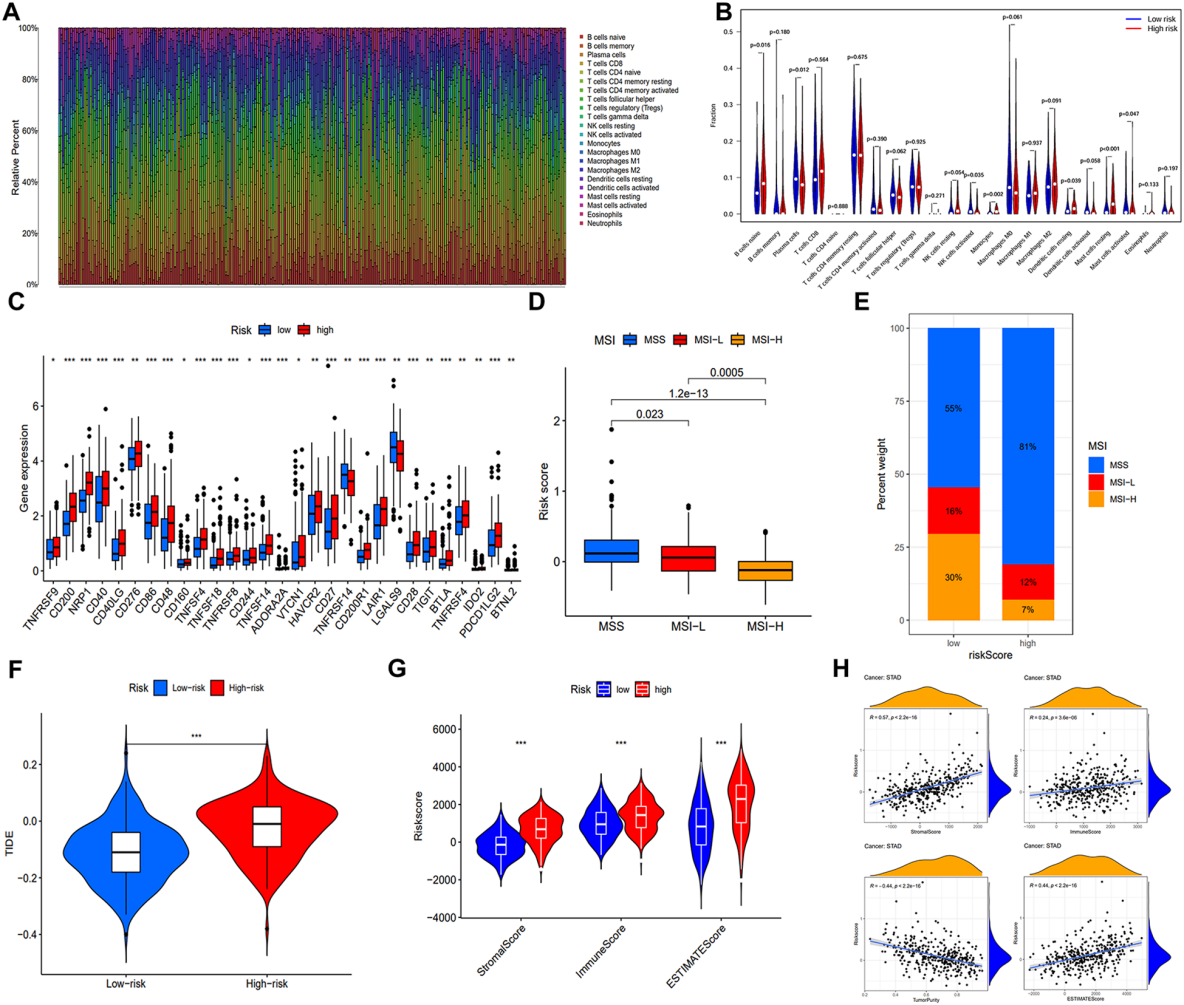
Immune microenvironment analysis and prediction for immunotherapy. **(A)** Heat map of immune cell distribution in the immune microenvironment of GC patients in training cohort. **(B)** Disparities in the allocation of various immune cells within the TME. **(C)** Differences of ICs expression. **(D)** The distribution of risk scores under three different microsatellite states. **(E)** The proportion of MSS, MSI-L, and MSI-H in different risk groups. **(F)** Comparative analysis of the TIDE score in both low- and high-risk populations. **(G)** ESTIMATE algorithm results for different risk groups. **(H)** Correlation analysis between four indicators of the ESTIMATE algorithm and the risk score. (*, **, ***, and ns represent p < 0.05, p < 0.01, p < 0.001, and “not statistically”, respectively.).

The TME, composed of diverse immune cells, cancer-associated fibroblasts (CAFs), endocrine cells, extracellular matrix (ECM) components, and other elements, significantly influences tumorigenesis. Disrupting the tumor immune tolerance feedback loop by targeting the TME is a promising strategy to enhance cancer therapy (16). To examine the correlation between our signature and immune infiltration, we employed the CIBERSORT algorithm to determine the composition of tumor-infiltrating immune cells in STAD (**Figure 7A**). Comparative analysis of immune cell distribution between high- and low-risk groups revealed significant differences. Plasma cells were notably reduced in the high-risk patients, whereas naïve B cells, activated NK cells monocytes, resting dendritic cells, and resting mast cells were increased (**Figure 7B**). Moreover, elevated expression of multiple ICs in high-risk patients suggested increased susceptibility to immune evasion (**Figure 7C**).

Subsequently, we examined the correlation between microsatellite status and risk score. **Figure 7D** indicated that patients with MSI-H, known for increased immunotherapy sensitivity, exhibited lower risk scores. As expected, the MSI-H prevalence was considerably higher in low-risk patients (30%) (**Figure 7E**). To predict immune system evasion, we performed TIDE analysis. As shown in **Figure 7F**, individuals at high risk expressed elevated TIDE scores, indicating a greater risk of immunological escape and reduced immunotherapy responsiveness. The ESTIMATE algorithm revealed significantly elevated stromal, immune, and estimate scores in the high-risk group, positively correlated with the risk score. Conversely, a negative correlation between tumor purity and the risk score was observed (**Figures 7G, H**).

### 3.7 Correlation of risk model with TMB

**Figure 8:**
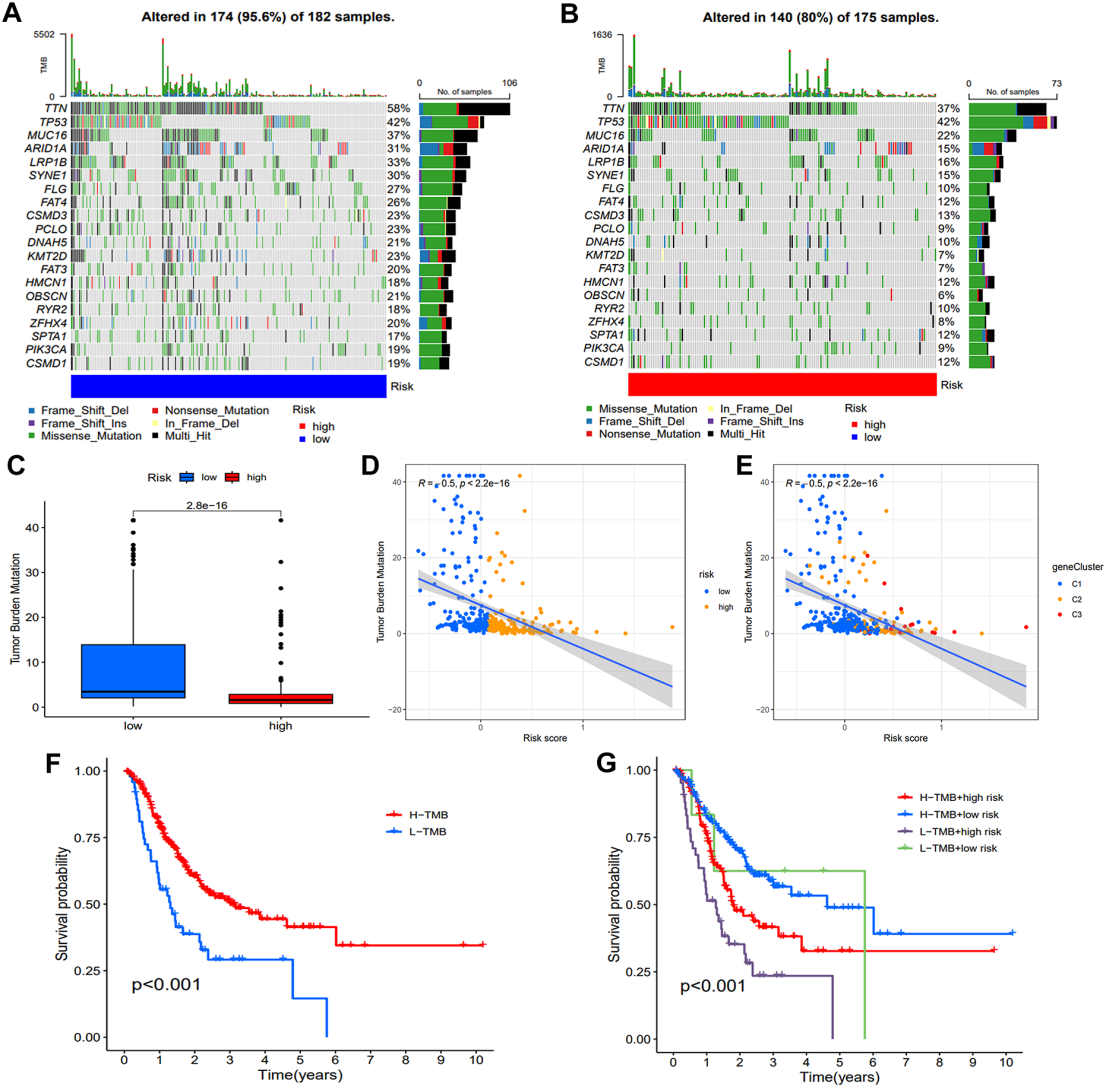
Assessment of TMB and genetic mutation profile. **(A, B)** The waterfall plot illustrated difference of somatic mutation characteristics. **(C)** The TMB difference between the two groups. **(D, E)** The correlation map demonstrates the associations between various risk groups and gene clusters with TMB. **(F)** Comparison of the survival probability. **(G)** An examination of survival rates was conducted on three groups of patients, combining their risk group and TMB group.

Human tumors exhibit varying levels of somatic mutations collectively termed TMB, which has been linked to immunotherapy efficacy (17, 18). To investigate the association between risk score and gene mutation, we analyzed simple nucleotide variation data from TCGA. **Figures 8A, B** present the top 20 genes with the highest frequency of mutations in two groups. TTN, TP53, MUC16, ARID1A, and LRP1B emerged as the five most prominent mutated genes. TMB analysis revealed an inverse relationship between TMB and risk score (**Figures 8C, D**). Spearman correlation analysis further differentiated clusters based on TMB and risk score (**Figure 8E**). Significantly, the high TMB had superior survival rates compared to the group with a low TMB (**Figure 8F**). An integrated survival analysis, including both TMB and risk groups (**Figure 8G**), demonstrated that GC patients with high TMB and low risk scores presented the most favorable outcome.

### 3.8 PPI network

To investigate the interaction of model genes, we constructed a PPI network. Next, the results of the STRING database were exported into Cytoscape for further analysis to obtain the hub gene (**Supplementary Figure S4A**). Then, by intersecting the top 7 hub genes determined by MCC, Stress, Degree and Closeness algorithms in cytohub plug-in, we identified 6 core genes (**Supplementary Figure S4B-F**). Moreover, the impact of IRAK1, SERPINE1, KIT, CXCL1, CD36, TXNIP on prognosis of STAD was analyzed by GEPIA online tools (**Supplementary Figure S5**). The findings indicated that lower expression levels of SERPINE1, KIT, CD36 and TXNIP were associated with longer OS, while differences in IRAK1 and CXCL1 expression levels did not have a statistically significant effect on prognosis.

### 3.9 Single-cell analysis of the model genes

We selected scRNA-seq data from GSE112302 dataset for further analysis of the model gene. To ensure the reliability of the single-cell data, we applied a filter to exclude genes expressed in fewer than three cells and cells expressing fewer than 50 genes (**Supplementary Figure S6A**). The correlation of sequencing depth with mitochondrial content and gene number was shown in the **Supplementary Figure S6B**. Subsequently, the data was standardized and the top 1,500 genes with significant intercellular coefficients of variation were extracted for further analysis (**Supplementary Figure S6C**). We then employed PCA analysis to reduce the dimensionality of the data (**Supplementary Figure S7A**). **Supplementary Figures S7B-C** illustrates the characteristic genes of the initial four principal components in the PCA analysis. We chose the initial 14 PCA components with a significance level of p<0.05 for subsequent analysis (**Supplementary Figure S7D**). The t-Distributed Stochastic Neighbor Embedding (tSNE) algorithm was applied to classify the cells into six distinct clusters, illustrating the global distribution of the single-cell transcriptomes (**Supplementary Figure S8A**). Each cluster represents a distinct cell population. **Supplementary Figure S8B** depicted, in the form of a heat map, the top 10 genes exhibiting the most substantial variances within each cluster. The distribution and expression of key model genes are visualized in **Supplementary Figures S8C, D**. Furthermore, **Supplementary Figure S8E** visualizes the expression of prognostic genes identified through the PPI network, including SERPINE1, KIT, CD36, and TXNIP, across the clusters: IRAK1 was significantly expressed in Clusters 4 and 5; SERPINE1 and KIT were not highly expressed in any of the clusters; CXCL1 showed high expression in Clusters 1 and 5; CD36 was predominantly expressed in Cluster 5; and TXNIP was most highly expressed in Clusters 0 and 2. Cell type annotation (**Supplementary Figure S8F**) reveals that the six clusters can be broadly classified into two major cell types: Clusters 0-4 primarily represent epithelial cells, while Cluster 5 is primarily composed of monocytes. Based on these findings, we conclude that IRAK1 and CXCL1 are expressed in both epithelial cells and monocytes, CD36 is predominantly expressed in monocytes, and TXNIP is mainly expressed in epithelial cells.

### 3.10 Knockdown of TXNIP inhibits the growth of gastric cancer cells

**Figure 9:**
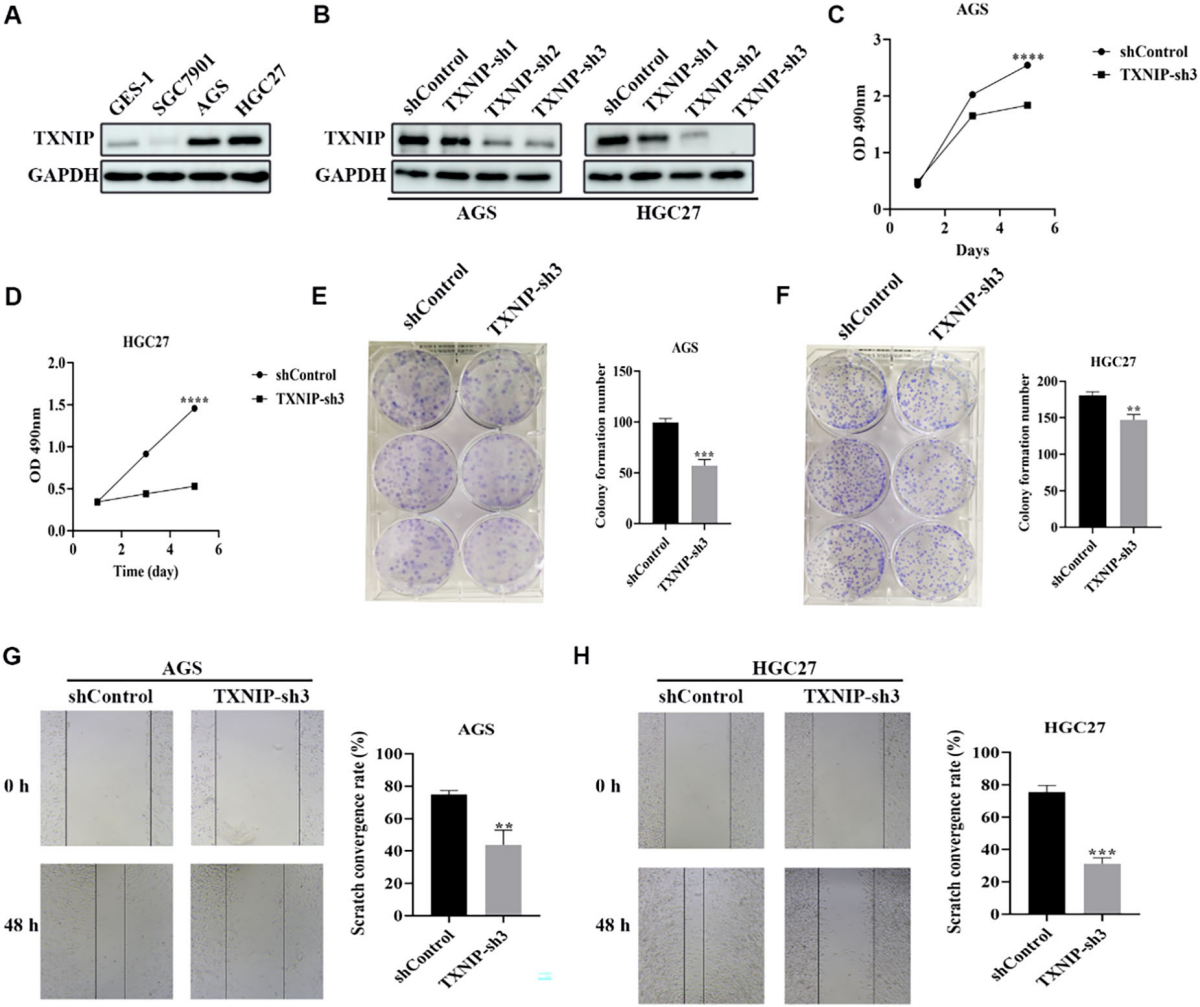
Knockdown of TXNIP inhibits the growth of gastric cancer cells. **(A)** Western blot revealed TXNIP expression levels in GES-1, SGC7901, AGS, HGC27 cell lines (n=3). **(B)** TXNIP protein expression was evaluated by western blot in AGS and HGC27 cells silenced by TXNIP-sh1, TXNIP-sh2, and TXNIP-sh3 (n=3). **(C, D)** Cell viability of AGS and HGC27 cell lines treated with lentivirus (shControl, TXNIP-sh3) was determined (n=3). **(E, F)** AGS and HGC27 cell lines cloning after lentivirus treatment (shControl, TXNIP-sh3). **(G, H)** Determination of migration ability of AGS and HGC27 cell lines treated with lentivirus (shControl, TXNIP-sh3) (n=3). Scale bar: 250 μm. The data were represented as mean ± standard deviation **p<0.01, ***p<0.001, and ****p<0.0001, with significant differences compared to the control group.

We initially examined TXNIP protein expression levels in gastric cancer cell lines SGC7901, AGS, and HGC27, as well as in normal human gastric mucosal epithelial cells GES-1. TXNIP protein expression was significantly higher in AGS and HGC27 cells compared to GES-1 cells (**Figure 9A**). To investigate the biological role of TXNIP in gastric cancer, we employed lentiviral transduction to knock down TXNIP gene expression in AGS and HGC27 cells. Based on knockdown efficiency, shRNA3 was selected for subsequent experiments (**Figure 9B**). MTT assays revealed that TXNIP knockdown significantly inhibited the growth of AGS and HGC27 cells, with a marked reduction in cell viability on days three and five (**Figures 9C, D**). Furthermore, colony formation and migration assays demonstrated that TXNIP knockdown suppressed the clonal formation and migratory capacity of AGS and HGC27 cells (**Figures 9E-H**). Overall, these results indicate that TXNIP exerts an oncogenic role in gastric cancer.

## 4 Discussion

Gastric cancer, originating from the epithelial cells of the gastric mucosa, is a globally prevalent and highly lethal malignant tumor (1, 19). While the morbidity and mortality rates of GC have declined in recent decades, largely attributed to advancements in multimodal treatments, China continues to bear 44% of the global disease burden, and overall patient survival remains a critical concern (20). Given the substantial heterogeneity of gastric cancer, personalized treatment is considered the optimal approach to reduce mortality and prolong survival. Advances in sequencing and bioinformatics technologies are empowering clinicians to refine patient assessments for personalized care.

While cancer and aging have traditionally been studied as distinct entities, a growing body of evidence underscores the intimate link between them, suggesting that cancer is an aging-related disease (21–24). Impaired macroautophagy and cellular senescence, both hallmarks of aging, exert context-dependent oncosuppressive and pro-tumorigenic influences (5). Furthermore, previous research has established the pivotal role of autophagy-senescence crosstalk in regulating tumor initiation and progression (25–29). By integrating autophagy- and senescence-related genes, we developed a novel prognostic signature that demonstrates exceptional predictive power and offers novel avenues for identifying potential therapeutic interventions in GC patients.

While autophagy has been investigated in GC using bioinformatics approaches (30–34), this study established a novel connection between autophagy and senescence to develop a robust prognostic model, further characterizing the TME, predicting immunotherapy efficacy, and assessing drug responsiveness in GC patients. Initially, common genes were identified among autophagy-related genes, senescence-related genes, and STAD-associated genes through intersection analysis. Subsequent differential expression analysis of these intersecting genes yielded 161 DEASRGs. Functional enrichment analyses revealed significant enrichment of these DEGs in cell cycle and carcinogenesis pathways. Univariate Cox regression identified 29 prognosis-associated genes, with frequent copy number variations confirming the critical involvement of ASRGs in GC lesions. LASSO regression selected 14 variables (PIM1, ITGB4, SPARC, CASP2, LMNB2, SERPINE1, TXNIP, UHRF1, IRAK1, KIT, CD36, CXCL1, ZFP36, MAP4K4) for inclusion in the final prognostic signature. KM curves revealed a statistically significant decrease in overall survival for individuals classified as high-risk. Time-dependent ROC curve validated the signature’s predictive performance, exhibiting high accuracy. Multivariate Cox regression confirmed the independent prognostic value of the derived risk scores. These findings suggest autophagy and senescence as potential therapeutic targets for GC, with the novel signature serving as a predictor of prognosis. To further explore ASRG-related modifications in GC, patients were classified into three distinct subtypes with significant prognostic differences based on gene expression profiles. This suggests three different ASRG-related modification modes in GC, each with unique clinical and immunological characteristics. Nomograms incorporating clinicopathological variables and the signature provided a comprehensive perspective on the predictive potential of ASRGs. External validation using the GSE66229 dataset confirmed the robustness of the prognostic risk model and nomogram.

We then conducted a comparative analysis of TME variations within risk subgroups. As a dynamic and complex ecosystem composed of various extracellular components and cell types, the crosstalk between cellular components and tumor cells is a critical factor in cancer pathogenesis and has emerged as a potential therapeutic target (35). Immune checkpoints (ICs) analysis indicated an immunosuppressive TME in the high-risk group. NK cells serve as an essential part in the innate immune response, capable of operating independently without prior sensitization. They can eliminate tumor cells through antibody-dependent cell-mediated cytotoxicity (ADCC) and trigger an adaptive immune response by releasing pro-inflammatory cytokines and chemokines (36).

Previous studies have demonstrated that a high abundance of NK cell infiltration within the TME was associated with favorable prognosis in certain malignancies (37). NK cells directly kill tumor cells. Additionally, NK cells can express death receptors, such as FasL, which bind to Fas on the tumor cell surface, triggering apoptosis (38). NK cells also secrete cytokines like IFN-γ and TNF-α. Recent studies have indicated that IFN-γ can upregulate MHC-I expression on the surface of tumor cells, thereby increasing their susceptibility to immune cell-mediated recognition (39). In contrast, TNF-α directly induces apoptosis in tumor cells. NKG2D is a stimulatory receptor located on the surface of NK cells. While NKG2D ligands are downregulated in normal tissues, their expression rapidly increases upon malignant transformation (40). Consequently, NKG2D is an ideal target for chimeric antigen receptor (CAR)-T cell therapy (41). Additionally, a research team has developed 70CAR-iNK cells, which express CD70-targeted CAR molecules (42). Dendritic cells (DCs) play a crucial role in the TME, serving as antigen-presenting cells that initiate specific immune responses. Beyond this, they also regulate the function of other immune cells. Studies have shown that IL-12 secreted by DCs can promote the differentiation of T cells into Th1 cells (43). Moreover, mature DCs can inhibit Treg activity by upregulating co-stimulatory molecules, thereby restoring the body’s anti-tumor immune response (44).

In the contemporary medical landscape, chemotherapy efficacy for GC has plateaued, while targeted therapies benefit only a small subset (10-12%) of the population. Immunotherapy, exemplified by programmed cell death protein 1 (PD-1) inhibitor antibodies, has demonstrated significant progress in GC treatment (45, 46). Previous research on immunotherapy response predictors has primarily focused on patients with elevated MSI, increased PD-L1 expression, higher tumor mutation burden, and Epstein-Barr virus positivity. However, identifying patients who benefit from immunotherapy may require additional clinical and molecular markers.

Microsatellites are DNA sequences composed of short, tandemly repeated units (typically 1 to 6 base pairs) with a high mutation rate. MSI arises from errors in DNA replication due to defective mismatch repair machinery, resulting in insertions or deletions within microsatellite sequences (47). MSI-H tumors exhibit increased immunogenicity across various tumor types, leading to an immune response from tumor-infiltrating lymphocytes (TILs). This heightened immunogenicity is responsible for the susceptibility of MSI-H tumors to immunotherapy.

Our signature identified a 4.3-fold higher proportion of MSI-H in the low-risk group, suggesting superior immunotherapy efficacy for the low-risk population. Immune checkpoint inhibitors (ICIs), a group of molecules expressed on immune cells that modulate immune activation, are central to immunotherapy (48). Analysis of ICs within the two risk subgroups revealed significantly increased ICIs expression in the high-risk populations. Consistent with these findings, the low-risk patients exhibited a considerably higher TMB. A higher TMB correlates with increased neoantigen presentation and enhanced T-cell recognition, leading to improved ICIs outcomes (49). Furthermore, TIDE scores corroborated these observations. The high-risk group demonstrated markedly elevated stromal scores. Excessive stromal components in the high-risk group might impair ICIs efficacy by impeding the infiltration of TILs and other immune cells into tumors (50, 51). Taken together, our novel signature provides a new perspective for accurately identifying individuals who may benefit from immunotherapy.

Four genes significantly associated with prognosis in GEPIA analysis were selected for further analysis. SERPINE1 promotes the proliferation and division of gastric cancer cells by upregulating positive cell cycle regulators, such as Cyclin D1 (52). Additionally, SERPINE1 can indirectly enhances the migratory and invasive abilities of gastric cancer cells by inhibiting plasminogen activators, like tPA and uPA (52). CD36 functions as a fatty acid transporter and plays a crucial role in metabolic reprogramming. By facilitating the uptake of fatty acids, CD36 supports the growth and drug resistance of gastric cancer cells (53). In gastric cancer, particularly in gastrointestinal stromal tumors (GISTs), mutations in the KIT gene are frequently observed. These mutations activate signaling pathways, including MAPK and PI3K/Akt, that promote cell survival, proliferation, migration, and invasion (54). As such, KIT inhibitors, are currently being investigated in clinical trials for their therapeutic potential in gastric cancer (55, 56).TXNIP functions primarily as a molecule that binds to TRX to regulate ROS and oxidative stress within cells. ROS are closely related to the initiation and development of autophagy and senescence. Increased expression of TXNIP can enhance the cytotoxicity of chemotherapy drugs by modulating ROS levels (57). Furthermore, overexpression of TXNIP leads to the upregulation of angiogenesis-related proteins and promotes an angiogenic phenotype (58). The NLRP3 inflammasome is involved in immune responses in various cancers, and numerous studies have highlighted the link between TXNIP and NLRP3 inflammasome activation (59). TXNIP is crucial for the maturation of NK cells and the function of DCs in the tumor microenvironment, thus influencing anti-tumor immunity (60–62). Initially, TXNIP was considered a potential tumor suppressor gene. Nevertheless, the findings obtained from diverse tumor studies utilizing varied methodologies exhibit paradox, suggesting that the role of TXNIP can be variable upon the specific tumor type and stage. These findings indicate that the involvement of TXNIP in cancer is intricate Some studies have demonstrated decreased TXNIP expression in several cancer types. Song et al. reported that TXNIP antisense cDNA transfection in melanoma cells reduced FasL and CD44 cytokine expression, confirming TXNIP’s role in promoting melanoma cell apoptosis and inhibiting tumor growth (63). In breast cancer, TXNIP knockdown increased Ki-67 expression (a marker of cell proliferation) and decreased p27 (a cell cycle regulatory protein), leading to enhanced breast cancer cell growth *in vitro* and *in vivo* (64–66). Furthermore, TXNIP mediates acetylation inhibitor-induced suppression of hepatocellular carcinoma by triggering potassium deprivation (67). The tumor-suppressive mechanism of TXNIP in lung cancer is likely attributed to its promotion of A2BR degradation and inhibition of cRaf/Erk signaling (68). In contrast, Elevated expression of TXNIP may also contribute to worse prognosis in some types of cancer.

For instance, in hepatocellular carcinoma (HCC) and renal clear cell carcinoma, the overexpression of TXNIP promotes angiogenesis and the spread of cancer cells (58, 69). Studies have also noted that lung cancer patients with high levels of TXNIP expression had reduced rates of progression-free survival (70). These studies demonstrate that the effects of TXNIP on tumors are characterized by tumor heterogeneity.

However, the precise role of TXNIP in GC remains poorly understood. TXNIP protein expression correlates with the prognosis of GC patients. A Pan-cancer analysis indicated a connection between TXNIP and an unfavorable outcome in gastric cancer (71). To elucidate TXNIP’s role in GC progression, this study downregulated TXNIP protein expression in gastric cancer cell lines, resulting in significant inhibition of cell viability, proliferation, and migration. Given previous findings on TXNIP’s involvement in ROS homeostasis, metabolic response, and immune function, TXNIP emerges as a promising therapeutic target for cancer treatment (71–74).

Despite these promising findings, our study has inherent limitations. Primarily, the research relied on publicly available databases, necessitating prospective, large-scale real-world investigations to validate the model’s generalizability. While we have experimentally confirmed key findings, additional exploration is necessary to clarify the underlying mechanisms governing the interplay between autophagy, senescence, and tumorigenesis. Additionally, the model’s complexity, involving many genes, hinders its practical application and necessitates optimization.

Conclusively, our study identified an entirely novel, fourteen-gene predictive signature associated with autophagy and senescence in GC patients, validated in an independent cohort. This prognostic model reliably and consistently predicts GC patient survival, providing a foundation for personalized treatment strategies. Additionally, our findings suggest that alterations in immune cell infiltration within the TME may underlie gastric cancer development. These results offer valuable insights for future research on GC prognosis and personalized therapy.

## Data Availability

The original contributions presented in the study are included in the article/**Supplementary Material**. Further inquiries can be directed to the corresponding authors.
