# Supplementary material for: A prognostic model based on autophagy-and senescence-related genes for gastric cancer: implications for immunotherapy and personalized treatment
Source: Front Oncol. 2025 Mar 20;15:1509771. doi: 10.3389/fonc.2025.1509771 (PMC11965130; doi:10.3389/fonc.2025.1509771)
Supplement: Supplementary file 1 [file DataSheet1.pdf]

id	labels
GSM3067368-GAS14-C-B1-sc1	Epithelial_cells
GSM3067368-GAS14-C-B1-sc10	Epithelial_cells
GSM3067368-GAS14-C-B1-sc11	Epithelial_cells
GSM3067368-GAS14-C-B1-sc12	Epithelial_cells
GSM3067368-GAS14-C-B1-sc13	Epithelial_cells
GSM3067368-GAS14-C-B1-sc14	Epithelial_cells
GSM3067368-GAS14-C-B1-sc15	Epithelial_cells
GSM3067368-GAS14-C-B1-sc16	Epithelial_cells
GSM3067368-GAS14-C-B1-sc17	Epithelial_cells
GSM3067368-GAS14-C-B1-sc18	Epithelial_cells
GSM3067368-GAS14-C-B1-sc19	Epithelial_cells
GSM3067368-GAS14-C-B1-sc2	Epithelial_cells
GSM3067368-GAS14-C-B1-sc20	Epithelial_cells
GSM3067368-GAS14-C-B1-sc21	Epithelial_cells
GSM3067368-GAS14-C-B1-sc22	Neuroepithelial_cell
GSM3067368-GAS14-C-B1-sc24	Epithelial_cells
GSM3067368-GAS14-C-B1-sc25	Epithelial_cells
GSM3067368-GAS14-C-B1-sc26	Epithelial_cells
GSM3067368-GAS14-C-B1-sc27	Epithelial_cells
GSM3067368-GAS14-C-B1-sc28	Epithelial_cells
GSM3067368-GAS14-C-B1-sc29	Epithelial_cells
GSM3067368-GAS14-C-B1-sc3	Monocyte
GSM3067368-GAS14-C-B1-sc30	Epithelial_cells
GSM3067368-GAS14-C-B1-sc31	Epithelial_cells
GSM3067368-GAS14-C-B1-sc32	Neurons
GSM3067368-GAS14-C-B1-sc33	Epithelial_cells
GSM3067368-GAS14-C-B1-sc35	Epithelial_cells
GSM3067368-GAS14-C-B1-sc36	Epithelial_cells
GSM3067368-GAS14-C-B1-sc37	Macrophage
GSM3067368-GAS14-C-B1-sc38	Epithelial_cells
GSM3067368-GAS14-C-B1-sc39	Epithelial_cells
GSM3067368-GAS14-C-B1-sc4	Epithelial_cells
GSM3067368-GAS14-C-B1-sc40	Epithelial_cells
GSM3067368-GAS14-C-B1-sc41	Epithelial_cells
GSM3067368-GAS14-C-B1-sc42	Epithelial_cells
GSM3067368-GAS14-C-B1-sc43	Epithelial_cells
GSM3067368-GAS14-C-B1-sc44	Epithelial_cells
GSM3067368-GAS14-C-B1-sc45	Epithelial_cells
GSM3067368-GAS14-C-B1-sc46	Epithelial_cells
GSM3067368-GAS14-C-B1-sc47	Epithelial_cells
GSM3067368-GAS14-C-B1-sc48	Epithelial_cells
GSM3067368-GAS14-C-B1-sc49	Epithelial_cells
GSM3067368-GAS14-C-B1-sc5	Epithelial_cells
GSM3067368-GAS14-C-B1-sc50	Epithelial_cells
GSM3067368-GAS14-C-B1-sc51	Epithelial_cells
GSM3067368-GAS14-C-B1-sc52	Epithelial_cells
GSM3067368-GAS14-C-B1-sc53	Epithelial_cells
GSM3067368-GAS14-C-B1-sc54	Epithelial_cells
GSM3067368-GAS14-C-B1-sc55	Epithelial_cells
GSM3067368-GAS14-C-B1-sc56	Epithelial_cells
GSM3067368-GAS14-C-B1-sc57	Epithelial_cells
GSM3067368-GAS14-C-B1-sc58	Epithelial_cells
GSM3067368-GAS14-C-B1-sc59	Epithelial_cells
GSM3067368-GAS14-C-B1-sc6	Epithelial_cells
GSM3067368-GAS14-C-B1-sc60	Epithelial_cells
GSM3067368-GAS14-C-B1-sc61	Epithelial_cells
GSM3067368-GAS14-C-B1-sc62	Epithelial_cells

GSM3067368-GAS14-C-B1-sc63	Fibroblasts
GSM3067368-GAS14-C-B1-sc64	Epithelial_cells
GSM3067368-GAS14-C-B1-sc65	Epithelial_cells
GSM3067368-GAS14-C-B1-sc66	Epithelial_cells
GSM3067368-GAS14-C-B1-sc67	Epithelial_cells
GSM3067368-GAS14-C-B1-sc68	Epithelial_cells
GSM3067368-GAS14-C-B1-sc69	Epithelial_cells
GSM3067368-GAS14-C-B1-sc7	Epithelial_cells
GSM3067368-GAS14-C-B1-sc70	Epithelial_cells
GSM3067368-GAS14-C-B1-sc71	Epithelial_cells
GSM3067368-GAS14-C-B1-sc72	Epithelial_cells
GSM3067368-GAS14-C-B1-sc73	Epithelial_cells
GSM3067368-GAS14-C-B1-sc74	Epithelial_cells
GSM3067368-GAS14-C-B1-sc75	Epithelial_cells
GSM3067368-GAS14-C-B1-sc76	Epithelial_cells
GSM3067368-GAS14-C-B1-sc77	Epithelial_cells
GSM3067368-GAS14-C-B1-sc78	Epithelial_cells
GSM3067368-GAS14-C-B1-sc79	Tissue_stem_cells
GSM3067368-GAS14-C-B1-sc8	Epithelial_cells
GSM3067368-GAS14-C-B1-sc80	Epithelial_cells
GSM3067368-GAS14-C-B1-sc81	Chondrocytes
GSM3067368-GAS14-C-B1-sc82	Epithelial_cells
GSM3067368-GAS14-C-B1-sc83	Epithelial_cells
GSM3067368-GAS14-C-B1-sc84	Monocyte
GSM3067368-GAS14-C-B1-sc85	Epithelial_cells
GSM3067368-GAS14-C-B1-sc86	Epithelial_cells
GSM3067368-GAS14-C-B1-sc87	Epithelial_cells
GSM3067368-GAS14-C-B1-sc88	Epithelial_cells
GSM3067368-GAS14-C-B1-sc89	Fibroblasts
GSM3067368-GAS14-C-B1-sc9	Epithelial_cells
GSM3067368-GAS14-C-B1-sc90	Epithelial_cells
GSM3067369-GAS14-C-B2-sc1	Epithelial_cells
GSM3067369-GAS14-C-B2-sc10	Epithelial_cells
GSM3067369-GAS14-C-B2-sc11	Epithelial_cells
GSM3067369-GAS14-C-B2-sc12	Epithelial_cells
GSM3067369-GAS14-C-B2-sc13	Epithelial_cells
GSM3067369-GAS14-C-B2-sc14	Epithelial_cells
GSM3067369-GAS14-C-B2-sc15	Epithelial_cells
GSM3067369-GAS14-C-B2-sc16	Epithelial_cells
GSM3067369-GAS14-C-B2-sc17	Epithelial_cells
GSM3067369-GAS14-C-B2-sc18	Epithelial_cells
GSM3067369-GAS14-C-B2-sc19	Epithelial_cells
GSM3067369-GAS14-C-B2-sc2	Epithelial_cells
GSM3067369-GAS14-C-B2-sc20	Epithelial_cells
GSM3067369-GAS14-C-B2-sc21	Epithelial_cells
GSM3067369-GAS14-C-B2-sc22	Epithelial_cells
GSM3067369-GAS14-C-B2-sc24	Epithelial_cells
GSM3067369-GAS14-C-B2-sc25	Epithelial_cells
GSM3067369-GAS14-C-B2-sc26	Epithelial_cells
GSM3067369-GAS14-C-B2-sc27	Epithelial_cells
GSM3067369-GAS14-C-B2-sc28	Epithelial_cells
GSM3067369-GAS14-C-B2-sc29	Epithelial_cells
GSM3067369-GAS14-C-B2-sc3	Epithelial_cells
GSM3067369-GAS14-C-B2-sc30	Epithelial_cells
GSM3067369-GAS14-C-B2-sc31	Epithelial_cells
GSM3067369-GAS14-C-B2-sc32	Epithelial_cells
GSM3067369-GAS14-C-B2-sc33	Epithelial_cells
GSM3067369-GAS14-C-B2-sc35	Epithelial_cells

GSM3067369-GAS14-C-B2-sc36	Epithelial_cells
GSM3067369-GAS14-C-B2-sc37	Epithelial_cells
GSM3067369-GAS14-C-B2-sc38	Epithelial_cells
GSM3067369-GAS14-C-B2-sc39	Epithelial_cells
GSM3067369-GAS14-C-B2-sc4	Epithelial_cells
GSM3067369-GAS14-C-B2-sc40	Epithelial_cells
GSM3067369-GAS14-C-B2-sc41	Epithelial_cells
GSM3067369-GAS14-C-B2-sc42	Epithelial_cells
GSM3067369-GAS14-C-B2-sc43	Epithelial_cells
GSM3067369-GAS14-C-B2-sc44	Epithelial_cells
GSM3067369-GAS14-C-B2-sc45	Epithelial_cells
GSM3067369-GAS14-C-B2-sc46	Epithelial_cells
GSM3067369-GAS14-C-B2-sc47	Epithelial_cells
GSM3067369-GAS14-C-B2-sc48	Epithelial_cells
GSM3067369-GAS14-C-B2-sc49	Epithelial_cells
GSM3067369-GAS14-C-B2-sc5	Epithelial_cells
GSM3067369-GAS14-C-B2-sc50	Epithelial_cells
GSM3067369-GAS14-C-B2-sc51	Epithelial_cells
GSM3067369-GAS14-C-B2-sc52	Epithelial_cells
GSM3067369-GAS14-C-B2-sc53	Epithelial_cells
GSM3067369-GAS14-C-B2-sc54	Epithelial_cells
GSM3067369-GAS14-C-B2-sc55	Epithelial_cells
GSM3067369-GAS14-C-B2-sc56	Epithelial_cells
GSM3067369-GAS14-C-B2-sc57	Epithelial_cells
GSM3067369-GAS14-C-B2-sc58	Epithelial_cells
GSM3067369-GAS14-C-B2-sc59	Epithelial_cells
GSM3067369-GAS14-C-B2-sc60	Epithelial_cells
GSM3067369-GAS14-C-B2-sc61	Epithelial_cells
GSM3067369-GAS14-C-B2-sc62	Epithelial_cells
GSM3067369-GAS14-C-B2-sc63	Epithelial_cells
GSM3067369-GAS14-C-B2-sc64	Epithelial_cells
GSM3067369-GAS14-C-B2-sc65	Epithelial_cells
GSM3067369-GAS14-C-B2-sc66	Epithelial_cells
GSM3067369-GAS14-C-B2-sc67	Epithelial_cells
GSM3067369-GAS14-C-B2-sc68	Epithelial_cells
GSM3067369-GAS14-C-B2-sc69	Epithelial_cells
GSM3067369-GAS14-C-B2-sc7	Epithelial_cells
GSM3067369-GAS14-C-B2-sc70	Epithelial_cells
GSM3067369-GAS14-C-B2-sc71	Epithelial_cells
GSM3067369-GAS14-C-B2-sc72	Epithelial_cells
GSM3067369-GAS14-C-B2-sc73	Epithelial_cells
GSM3067369-GAS14-C-B2-sc74	Epithelial_cells
GSM3067369-GAS14-C-B2-sc75	Epithelial_cells
GSM3067369-GAS14-C-B2-sc76	Epithelial_cells
GSM3067369-GAS14-C-B2-sc77	Epithelial_cells
GSM3067369-GAS14-C-B2-sc78	Epithelial_cells
GSM3067369-GAS14-C-B2-sc79	Epithelial_cells
GSM3067369-GAS14-C-B2-sc8	Epithelial_cells
GSM3067369-GAS14-C-B2-sc80	Epithelial_cells
GSM3067369-GAS14-C-B2-sc81	Epithelial_cells
GSM3067369-GAS14-C-B2-sc82	Epithelial_cells
GSM3067369-GAS14-C-B2-sc83	Epithelial_cells
GSM3067369-GAS14-C-B2-sc84	Epithelial_cells
GSM3067369-GAS14-C-B2-sc85	Epithelial_cells
GSM3067369-GAS14-C-B2-sc86	Epithelial_cells
GSM3067369-GAS14-C-B2-sc87	Epithelial_cells
GSM3067369-GAS14-C-B2-sc88	Epithelial_cells
GSM3067369-GAS14-C-B2-sc89	Epithelial_cells

GSM3067369-GAS14-C-B2-sc9	Epithelial_cells
GSM3067369-GAS14-C-B2-sc90	Epithelial_cells
GSM3067370-GAS14-C-B3-sc67	Epithelial_cells
GSM3067370-GAS14-C-B3-sc68	Epithelial_cells
GSM3067370-GAS14-C-B3-sc69	Epithelial_cells
GSM3067370-GAS14-C-B3-sc70	Epithelial_cells
GSM3067370-GAS14-C-B3-sc71	Epithelial_cells
GSM3067370-GAS14-C-B3-sc72	Epithelial_cells
GSM3067370-GAS14-C-B3-sc73	Epithelial_cells
GSM3067370-GAS14-C-B3-sc74	Epithelial_cells
GSM3067370-GAS14-C-B3-sc75	Epithelial_cells
GSM3067370-GAS14-C-B3-sc76	Epithelial_cells
GSM3067370-GAS14-C-B3-sc77	Epithelial_cells
GSM3067370-GAS14-C-B3-sc78	Epithelial_cells
GSM3067370-GAS14-C-B3-sc79	Epithelial_cells
GSM3067370-GAS14-C-B3-sc80	Epithelial_cells
GSM3067370-GAS14-C-B3-sc81	Epithelial_cells
GSM3067370-GAS14-C-B3-sc82	Epithelial_cells
GSM3067370-GAS14-C-B3-sc83	Epithelial_cells
GSM3067370-GAS14-C-B3-sc84	Epithelial_cells
GSM3067370-GAS14-C-B3-sc85	Epithelial_cells
GSM3067370-GAS14-C-B3-sc86	Epithelial_cells
GSM3067370-GAS14-C-B3-sc87	Epithelial_cells
GSM3067370-GAS14-C-B3-sc88	Epithelial_cells
GSM3067370-GAS14-C-B3-sc89	Epithelial_cells
GSM3067370-GAS14-C-B3-sc90	Epithelial_cells
GSM3067373-GAS15-C-B1-sc1	Epithelial_cells
GSM3067373-GAS15-C-B1-sc10	Epithelial_cells
GSM3067373-GAS15-C-B1-sc11	Epithelial_cells
GSM3067373-GAS15-C-B1-sc12	Epithelial_cells
GSM3067373-GAS15-C-B1-sc13	Epithelial_cells
GSM3067373-GAS15-C-B1-sc14	Epithelial_cells
GSM3067373-GAS15-C-B1-sc15	Macrophage
GSM3067373-GAS15-C-B1-sc16	Epithelial_cells
GSM3067373-GAS15-C-B1-sc17	Epithelial_cells
GSM3067373-GAS15-C-B1-sc18	Epithelial_cells
GSM3067373-GAS15-C-B1-sc19	Epithelial_cells
GSM3067373-GAS15-C-B1-sc2	Epithelial_cells
GSM3067373-GAS15-C-B1-sc20	Epithelial_cells
GSM3067373-GAS15-C-B1-sc21	Epithelial_cells
GSM3067373-GAS15-C-B1-sc22	Smooth_muscle_cells
GSM3067373-GAS15-C-B1-sc24	Monocyte
GSM3067373-GAS15-C-B1-sc25	Fibroblasts
GSM3067373-GAS15-C-B1-sc26	Epithelial_cells
GSM3067373-GAS15-C-B1-sc27	Epithelial_cells
GSM3067373-GAS15-C-B1-sc28	Epithelial_cells
GSM3067373-GAS15-C-B1-sc29	Epithelial_cells
GSM3067373-GAS15-C-B1-sc3	Epithelial_cells
GSM3067373-GAS15-C-B1-sc30	Smooth_muscle_cells
GSM3067373-GAS15-C-B1-sc31	B_cell
GSM3067373-GAS15-C-B1-sc32	Epithelial_cells
GSM3067373-GAS15-C-B1-sc33	Epithelial_cells
GSM3067373-GAS15-C-B1-sc35	Macrophage
GSM3067373-GAS15-C-B1-sc36	Epithelial_cells
GSM3067373-GAS15-C-B1-sc37	Epithelial_cells
GSM3067373-GAS15-C-B1-sc38	Myelocyte
GSM3067373-GAS15-C-B1-sc39	Macrophage
GSM3067373-GAS15-C-B1-sc4	Epithelial_cells

GSM3067373-GAS15-C-B1-sc40	Epithelial_cells
GSM3067373-GAS15-C-B1-sc41	Epithelial_cells
GSM3067373-GAS15-C-B1-sc42	Epithelial_cells
GSM3067373-GAS15-C-B1-sc43	Epithelial_cells
GSM3067373-GAS15-C-B1-sc44	Epithelial_cells
GSM3067373-GAS15-C-B1-sc45	Epithelial_cells
GSM3067373-GAS15-C-B1-sc46	Fibroblasts
GSM3067373-GAS15-C-B1-sc47	Fibroblasts
GSM3067373-GAS15-C-B1-sc48	Epithelial_cells
GSM3067373-GAS15-C-B1-sc49	Epithelial_cells
GSM3067373-GAS15-C-B1-sc5	Epithelial_cells
GSM3067373-GAS15-C-B1-sc50	Fibroblasts
GSM3067373-GAS15-C-B1-sc51	Fibroblasts
GSM3067373-GAS15-C-B1-sc52	Epithelial_cells
GSM3067373-GAS15-C-B1-sc53	Epithelial_cells
GSM3067373-GAS15-C-B1-sc54	Epithelial_cells
GSM3067373-GAS15-C-B1-sc55	Macrophage
GSM3067373-GAS15-C-B1-sc56	Fibroblasts
GSM3067373-GAS15-C-B1-sc57	Epithelial_cells
GSM3067373-GAS15-C-B1-sc58	Epithelial_cells
GSM3067373-GAS15-C-B1-sc59	Epithelial_cells
GSM3067373-GAS15-C-B1-sc6	Epithelial_cells
GSM3067373-GAS15-C-B1-sc60	Epithelial_cells
GSM3067373-GAS15-C-B1-sc61	Macrophage
GSM3067373-GAS15-C-B1-sc62	Epithelial_cells
GSM3067373-GAS15-C-B1-sc63	Epithelial_cells
GSM3067373-GAS15-C-B1-sc64	Epithelial_cells
GSM3067373-GAS15-C-B1-sc65	Epithelial_cells
GSM3067373-GAS15-C-B1-sc66	Epithelial_cells
GSM3067373-GAS15-C-B1-sc67	Epithelial_cells
GSM3067373-GAS15-C-B1-sc68	Fibroblasts
GSM3067373-GAS15-C-B1-sc69	Epithelial_cells
GSM3067373-GAS15-C-B1-sc7	Endothelial_cells
GSM3067373-GAS15-C-B1-sc70	Smooth_muscle_cells
GSM3067373-GAS15-C-B1-sc71	Epithelial_cells
GSM3067373-GAS15-C-B1-sc72	Epithelial_cells
GSM3067373-GAS15-C-B1-sc73	Epithelial_cells
GSM3067373-GAS15-C-B1-sc74	Epithelial_cells
GSM3067373-GAS15-C-B1-sc75	Epithelial_cells
GSM3067373-GAS15-C-B1-sc76	Epithelial_cells
GSM3067373-GAS15-C-B1-sc77	Epithelial_cells
GSM3067373-GAS15-C-B1-sc78	Epithelial_cells
GSM3067373-GAS15-C-B1-sc79	Epithelial_cells
GSM3067373-GAS15-C-B1-sc8	Tissue_stem_cells
GSM3067373-GAS15-C-B1-sc80	Epithelial_cells
GSM3067373-GAS15-C-B1-sc81	Epithelial_cells
GSM3067373-GAS15-C-B1-sc82	Epithelial_cells
GSM3067373-GAS15-C-B1-sc83	Epithelial_cells
GSM3067373-GAS15-C-B1-sc84	Epithelial_cells
GSM3067373-GAS15-C-B1-sc85	Epithelial_cells
GSM3067373-GAS15-C-B1-sc86	Epithelial_cells
GSM3067373-GAS15-C-B1-sc87	Monocyte
GSM3067373-GAS15-C-B1-sc88	Epithelial_cells
GSM3067373-GAS15-C-B1-sc89	Epithelial_cells
GSM3067373-GAS15-C-B1-sc9	Epithelial_cells
GSM3067373-GAS15-C-B1-sc90	Epithelial_cells
GSM3067374-GAS15-C-B2-sc1	Epithelial_cells
GSM3067374-GAS15-C-B2-sc10	Epithelial_cells

GSM3067374-GAS15-C-B2-sc11	Epithelial_cells
GSM3067374-GAS15-C-B2-sc12	Macrophage
GSM3067374-GAS15-C-B2-sc13	Epithelial_cells
GSM3067374-GAS15-C-B2-sc14	Epithelial_cells
GSM3067374-GAS15-C-B2-sc15	Epithelial_cells
GSM3067374-GAS15-C-B2-sc16	Epithelial_cells
GSM3067374-GAS15-C-B2-sc17	Monocyte
GSM3067374-GAS15-C-B2-sc18	Epithelial_cells
GSM3067374-GAS15-C-B2-sc19	Epithelial_cells
GSM3067374-GAS15-C-B2-sc2	Macrophage
GSM3067374-GAS15-C-B2-sc20	Epithelial_cells
GSM3067374-GAS15-C-B2-sc21	Epithelial_cells
GSM3067374-GAS15-C-B2-sc22	Epithelial_cells
GSM3067374-GAS15-C-B2-sc23	Pro-Myelocyte
GSM3067374-GAS15-C-B2-sc24	Epithelial_cells
GSM3067374-GAS15-C-B2-sc25	Epithelial_cells
GSM3067374-GAS15-C-B2-sc26	Epithelial_cells
GSM3067374-GAS15-C-B2-sc27	Epithelial_cells
GSM3067374-GAS15-C-B2-sc28	Epithelial_cells
GSM3067374-GAS15-C-B2-sc29	Macrophage
GSM3067374-GAS15-C-B2-sc3	Epithelial_cells
GSM3067374-GAS15-C-B2-sc30	Epithelial_cells
GSM3067374-GAS15-C-B2-sc31	Epithelial_cells
GSM3067374-GAS15-C-B2-sc32	Epithelial_cells
GSM3067374-GAS15-C-B2-sc33	Epithelial_cells
GSM3067374-GAS15-C-B2-sc35	Tissue_stem_cells
GSM3067374-GAS15-C-B2-sc36	Epithelial_cells
GSM3067374-GAS15-C-B2-sc37	Macrophage
GSM3067374-GAS15-C-B2-sc38	Epithelial_cells
GSM3067374-GAS15-C-B2-sc39	Epithelial_cells
GSM3067374-GAS15-C-B2-sc4	Epithelial_cells
GSM3067374-GAS15-C-B2-sc40	Epithelial_cells
GSM3067374-GAS15-C-B2-sc41	Epithelial_cells
GSM3067374-GAS15-C-B2-sc42	Epithelial_cells
GSM3067374-GAS15-C-B2-sc43	Epithelial_cells
GSM3067374-GAS15-C-B2-sc44	Epithelial_cells
GSM3067374-GAS15-C-B2-sc45	Macrophage
GSM3067374-GAS15-C-B2-sc46	Epithelial_cells
GSM3067374-GAS15-C-B2-sc47	Epithelial_cells
GSM3067374-GAS15-C-B2-sc48	Epithelial_cells
GSM3067374-GAS15-C-B2-sc49	Epithelial_cells
GSM3067374-GAS15-C-B2-sc5	Epithelial_cells
GSM3067374-GAS15-C-B2-sc50	Epithelial_cells
GSM3067374-GAS15-C-B2-sc51	Epithelial_cells
GSM3067374-GAS15-C-B2-sc52	Epithelial_cells
GSM3067374-GAS15-C-B2-sc53	Epithelial_cells
GSM3067374-GAS15-C-B2-sc54	Epithelial_cells
GSM3067374-GAS15-C-B2-sc55	Epithelial_cells
GSM3067374-GAS15-C-B2-sc56	Epithelial_cells
GSM3067374-GAS15-C-B2-sc57	Epithelial_cells
GSM3067374-GAS15-C-B2-sc58	Macrophage
GSM3067374-GAS15-C-B2-sc59	Epithelial_cells
GSM3067374-GAS15-C-B2-sc6	Tissue_stem_cells
GSM3067374-GAS15-C-B2-sc60	Epithelial_cells
GSM3067374-GAS15-C-B2-sc61	Epithelial_cells
GSM3067374-GAS15-C-B2-sc62	Epithelial_cells
GSM3067374-GAS15-C-B2-sc63	Macrophage
GSM3067374-GAS15-C-B2-sc64	Epithelial_cells

GSM3067374-GAS15-C-B2-sc65	Epithelial_cells
GSM3067374-GAS15-C-B2-sc66	Epithelial_cells
GSM3067374-GAS15-C-B2-sc67	Epithelial_cells
GSM3067374-GAS15-C-B2-sc68	Epithelial_cells
GSM3067374-GAS15-C-B2-sc69	Epithelial_cells
GSM3067374-GAS15-C-B2-sc7	Epithelial_cells
GSM3067374-GAS15-C-B2-sc70	Epithelial_cells
GSM3067374-GAS15-C-B2-sc71	Epithelial_cells
GSM3067374-GAS15-C-B2-sc72	Endothelial_cells
GSM3067374-GAS15-C-B2-sc73	Epithelial_cells
GSM3067374-GAS15-C-B2-sc74	Epithelial_cells
GSM3067374-GAS15-C-B2-sc75	Epithelial_cells
GSM3067374-GAS15-C-B2-sc76	Epithelial_cells
GSM3067374-GAS15-C-B2-sc77	Epithelial_cells
GSM3067374-GAS15-C-B2-sc78	Epithelial_cells
GSM3067374-GAS15-C-B2-sc79	Epithelial_cells
GSM3067374-GAS15-C-B2-sc8	Epithelial_cells
GSM3067374-GAS15-C-B2-sc80	Epithelial_cells
GSM3067374-GAS15-C-B2-sc81	Epithelial_cells
GSM3067374-GAS15-C-B2-sc82	Epithelial_cells
GSM3067374-GAS15-C-B2-sc83	Epithelial_cells
GSM3067374-GAS15-C-B2-sc84	Macrophage
GSM3067374-GAS15-C-B2-sc85	Epithelial_cells
GSM3067374-GAS15-C-B2-sc86	Epithelial_cells
GSM3067374-GAS15-C-B2-sc87	Epithelial_cells
GSM3067374-GAS15-C-B2-sc88	Epithelial_cells
GSM3067374-GAS15-C-B2-sc89	Macrophage
GSM3067374-GAS15-C-B2-sc9	Epithelial_cells
GSM3067374-GAS15-C-B2-sc90	Epithelial_cells
GSM3067375-GAS15-C-B3-sc67	Macrophage
GSM3067375-GAS15-C-B3-sc68	Epithelial_cells
GSM3067375-GAS15-C-B3-sc69	Tissue_stem_cells
GSM3067375-GAS15-C-B3-sc70	Epithelial_cells
GSM3067375-GAS15-C-B3-sc71	Epithelial_cells
GSM3067375-GAS15-C-B3-sc72	Epithelial_cells
GSM3067375-GAS15-C-B3-sc73	Epithelial_cells
GSM3067375-GAS15-C-B3-sc74	Epithelial_cells
GSM3067375-GAS15-C-B3-sc75	Epithelial_cells
GSM3067375-GAS15-C-B3-sc76	Endothelial_cells
GSM3067375-GAS15-C-B3-sc77	Epithelial_cells
GSM3067375-GAS15-C-B3-sc78	Epithelial_cells
GSM3067375-GAS15-C-B3-sc79	Epithelial_cells
GSM3067375-GAS15-C-B3-sc80	Smooth_muscle_cells
GSM3067375-GAS15-C-B3-sc81	Epithelial_cells
GSM3067375-GAS15-C-B3-sc82	Macrophage
GSM3067375-GAS15-C-B3-sc83	Tissue_stem_cells
GSM3067375-GAS15-C-B3-sc84	Epithelial_cells
GSM3067375-GAS15-C-B3-sc85	Epithelial_cells
GSM3067375-GAS15-C-B3-sc86	Epithelial_cells
GSM3067375-GAS15-C-B3-sc87	Macrophage
GSM3067375-GAS15-C-B3-sc88	Epithelial_cells
GSM3067375-GAS15-C-B3-sc89	Tissue_stem_cells
GSM3067375-GAS15-C-B3-sc90	Epithelial_cells
