# Supplementary material for: A prognostic model based on autophagy-and senescence-related genes for gastric cancer: implications for immunotherapy and personalized treatment
Source: Front Oncol. 2025 Mar 20;15:1509771. doi: 10.3389/fonc.2025.1509771 (PMC11965130; doi:10.3389/fonc.2025.1509771)
Supplement: Supplementary file 2 [file DataSheet2.pdf]

id	labels
0	Epithelial_cells
1	Epithelial_cells
2	Epithelial_cells
3	Epithelial_cells
4	Epithelial_cells
5	Monocyte
