# Supplementary material for: A prognostic model based on autophagy-and senescence-related genes for gastric cancer: implications for immunotherapy and personalized treatment
Source: Front Oncol. 2025 Mar 20;15:1509771. doi: 10.3389/fonc.2025.1509771 (PMC11965130; doi:10.3389/fonc.2025.1509771)
Supplement: Supplementary file 3 [file DataSheet3.pdf]

p_val	avg_log2F	pct.1	pct.2	p_val_adj	cluster	gene
4.8421193	-7.74656	0.016	0.826	8.61316190704553e-52	Epithelial_cells	SPI1
2.8381433	-6.47797	0.029	0.87	5.04848936848425e-46	Epithelial_cells	STAB1
4.2020350	-7.43905	0.058	0.957	7.47458001553445e-41	Epithelial_cells	CD4
1.4453063	-6.16341	0.021	0.739	2.57091086326806e-40	Epithelial_cells	WAS
4.5308119	-7.77942	0.034	0.826	8.05940837546452e-40	Epithelial_cells	HCK
6.3430011	-6.99963	0.013	0.652	1.12829303965285e-38	Epithelial_cells	ARHGAP9
1.3118593	-7.56359	0.064	0.957	2.33353549141394e-38	Epithelial_cells	IGSF6
1.0038903	-7.9513	0.011	0.609	1.78572013715704e-37	Epithelial_cells	SIGLEC9
1.9469720	-8.59631	0.021	0.696	3.46327380243695e-37	Epithelial_cells	SCIMP
2.9865074	-7.43606	0.021	0.696	5.31239951648416e-37	Epithelial_cells	LY86
3.5531981	-7.07441	0.016	0.652	6.32042895168389e-37	Epithelial_cells	LOC653786
4.6013163	-7.68318	0.072	0.957	8.18482159767548e-37	Epithelial_cells	LILRB4
8.4546918	-5.51626	0.045	0.826	1.50392058979775e-35	Epithelial_cells	PARVG
1.6611891	-7.71112	0.019	0.652	2.95492333551637e-35	Epithelial_cells	NCKAP1L
5.0863000	-5.2424	0.077	0.957	9.04751057536636e-35	Epithelial_cells	FYB
8.3199093	-8.15932	0.095	1	1.47994546813852e-34	Epithelial_cells	CD86
1.0524962	-7.19071	0.058	0.87	1.87218041439784e-34	Epithelial_cells	LAT2
1.4826176	-7.54244	0.04	0.783	2.63728032924486e-34	Epithelial_cells	LILRB2
1.0702184	-7.27067	0.021	0.652	1.90370457564885e-33	Epithelial_cells	PIK3R5
3.3323775	-7.54878	0.053	0.826	5.92763322447451e-33	Epithelial_cells	SLAMF8
7.3937508	-7.82422	0.09	0.957	1.31520040358467e-32	Epithelial_cells	PTPRC
2.0949809	-7.33611	0.066	0.87	3.72655206531053e-32	Epithelial_cells	CD300A
3.1160058	-6.7602	0.045	0.783	5.54275115076699e-32	Epithelial_cells	BCAT1
7.6880932	-6.26305	0.024	0.652	1.367558032116e-31	Epithelial_cells	CD72
2.7760090	-4.3015	0.058	0.826	4.93796490076588e-31	Epithelial_cells	NCF2
2.8318873	-6.67636	0.069	0.87	5.03736124904781e-31	Epithelial_cells	CELF2
3.8237709	-6.83873	0.082	0.913	6.80172369225547e-31	Epithelial_cells	MPEG1
6.6740358	-4.06523	0.04	0.739	1.18717749141166e-30	Epithelial_cells	DOCK10
1.6948109	-6.03105	0.085	0.913	3.01472976545431e-30	Epithelial_cells	FGL2
1.7549450	-7.35116	0.074	0.87	3.12169628899076e-30	Epithelial_cells	SLC37A2
2.6250130	-7.31492	0.021	0.609	4.6693732722462e-30	Epithelial_cells	RNASE2
3.5038547	-6.91099	0.021	0.609	6.2326568528348e-30	Epithelial_cells	GPR132
3.9329975	-7.56552	0.053	0.783	6.99601599369591e-30	Epithelial_cells	SLA
8.4783343	-7.87605	0.09	0.913	1.5081261176609e-29	Epithelial_cells	PLEK
1.5893496	-8.29094	0.066	0.826	2.82713511518224e-29	Epithelial_cells	PILRA
1.6337933	-7.19733	0.029	0.652	2.90619158912147e-29	Epithelial_cells	CCR1
2.1640139	-8.36639	0.056	0.783	3.84934795256337e-29	Epithelial_cells	FPR3
2.3690212	-6.60625	0.045	0.739	4.21401495526886e-29	Epithelial_cells	DOCK2
5.7706424	-5.58832	0.029	0.652	1.02648187971263e-28	Epithelial_cells	CD37
2.6474015	-6.85895	0.04	0.696	4.70919787414709e-28	Epithelial_cells	NCF4
3.6032086	-5.98937	0.058	0.783	6.40938754474914e-28	Epithelial_cells	EVI2B
1.3277545	-7.80268	0.027	0.609	2.36180981733499e-27	Epithelial_cells	SIGLEC1
1.4289429	-7.3919	0.103	0.913	2.54180364933891e-27	Epithelial_cells	LST1
2.2586175	-7.21482	0.042	0.696	4.0176288889543e-27	Epithelial_cells	MNDA
2.3657373	-6.87196	0.042	0.696	4.20817359681507e-27	Epithelial_cells	ADGRE2
4.0250793	-7.64378	0.003	0.391	7.15981121433951e-27	Epithelial_cells	LUCAT1
7.1754304	-4.64355	0.05	0.739	1.27636556235776e-26	Epithelial_cells	LRRC25
7.6711083	-6.97207	0.053	0.739	1.3645367460705e-26	Epithelial_cells	SLC8A1
8.2186381	-6.75662	0.021	0.565	1.46193135280931e-26	Epithelial_cells	CST7
8.5350982	-5.17512	0.061	0.783	1.51822327308297e-26	Epithelial_cells	EPB41L3
2.9678121	-7.52236	0.037	0.652	5.27914433686696e-26	Epithelial_cells	CD209
3.3451190	-6.74807	0.056	0.739	5.95029782304985e-26	Epithelial_cells	SIGLEC12
4.6700759	-8.70295	0.114	0.913	8.30713117270542e-26	Epithelial_cells	CD163
1.1728994	-5.64617	0.056	0.739	2.08635350655818e-25	Epithelial_cells	ZNF385A
1.9290301	-5.18209	0.13	0.957	3.43135885640109e-25	Epithelial_cells	ZEB2
2.0137181	-6.27517	0.082	0.826	3.5820019025486e-25	Epithelial_cells	IL10RA
2.1215909	-4.58341	0.058	0.739	3.77388595322836e-25	Epithelial_cells	C3AR1

2.2479867	-5.78171	0.082	0.826	3.99871887975816e-25	Epithelial_cells	MRC1
4.6670392	-5.80396	0.156	1	8.30172936946182e-25	Epithelial_cells	SLC2A3
7.0947469	-8.21168	0.122	0.913	1.26201358888188e-24	Epithelial_cells	MS4A4A
8.5304243	-3.91955	0.019	0.522	1.51739187744021e-24	Epithelial_cells	TFEC
9.7053041	-6.08939	0.032	0.609	1.72637949429507e-24	Epithelial_cells	GPR84
1.7525268	-8.41188	0.09	0.826	3.1173947552672e-24	Epithelial_cells	PLA2G7
2.7344993	-7.19317	0.005	0.391	4.86412742787866e-24	Epithelial_cells	MS4A14
1.4385090	-6.22959	0.034	0.609	2.55881996666241e-23	Epithelial_cells	HVCN1
2.0513408	-6.74798	0.003	0.348	3.64892510173093e-23	Epithelial_cells	TLR8
2.1261254	-7.17363	0.011	0.435	3.78195194005354e-23	Epithelial_cells	CD300LF
2.2436625	-5.17253	0.021	0.522	3.9910269645106e-23	Epithelial_cells	WDFY4
3.1592723	-6.28735	0.156	0.957	5.61971363316526e-23	Epithelial_cells	CSF1R
8.5646793	-4.63483	0.082	0.783	1.52348516730504e-22	Epithelial_cells	C1orf162
9.3682386	-4.98335	0.056	0.696	1.66642228896005e-22	Epithelial_cells	DOK3
1.0226725	-4.51911	0.016	0.478	1.81912994244639e-22	Epithelial_cells	LILRB1
1.0702923	-5.68333	0.037	0.609	1.90383595281311e-22	Epithelial_cells	ABI3
1.0934912	-6.52615	0.138	0.913	1.94510220164088e-22	Epithelial_cells	LAIR1
1.1899491	-6.57595	0.098	0.826	2.11668158501215e-22	Epithelial_cells	SAMSN1
1.9618526	-7.13624	0.196	1	3.48974355059852e-22	Epithelial_cells	MS4A7
2.0212563	-5.26043	0.098	0.826	3.59541083497962e-22	Epithelial_cells	MYO5A
2.1689598	-7.82827	0.143	0.913	3.85814576444336e-22	Epithelial_cells	SLC11A1
2.3119275	-6.67412	0.085	0.783	4.1124567981916e-22	Epithelial_cells	IFITM10
2.5536870	-5.45	0.135	0.913	4.54249843992616e-22	Epithelial_cells	STX11
3.7117176	-6.21477	0.024	0.522	6.60240334794029e-22	Epithelial_cells	CYTH4
4.9240559	-4.29342	0.093	0.826	8.75891067753544e-22	Epithelial_cells	LSP1
7.6788150	-6.80662	0.146	0.913	1.36590761720575e-21	Epithelial_cells	CYBB
1.0058167	-4.67631	0.05	0.652	1.78914690492731e-21	Epithelial_cells	NLRP3
1.0845029	-4.34634	0.024	0.522	1.9291137979089e-21	Epithelial_cells	IKZF1
1.3489157	-6.3418	0.019	0.478	2.39945128128402e-21	Epithelial_cells	NKG7
1.6414291	-4.24666	0.106	0.826	2.91977409909175e-21	Epithelial_cells	MPP1
1.7489224	-6.22674	0.106	0.826	3.11098333677806e-21	Epithelial_cells	HCLS1
2.1514917	-6.73878	0.013	0.435	3.82707344050908e-21	Epithelial_cells	CD80
9.6959308	-4.04548	0.13	0.87	1.72471218219136e-20	Epithelial_cells	CLEC7A
9.8582970	-4.69356	0.186	0.957	1.75359388284511e-20	Epithelial_cells	FCGR2A
1.1004992	-5.62668	0.09	0.783	1.95756798631266e-20	Epithelial_cells	FCGR1A
1.3419511	-7.68963	0.045	0.609	2.38706274526129e-20	Epithelial_cells	OTOA
1.3986728	-7.46952	0.045	0.609	2.48795925647676e-20	Epithelial_cells	LINC01272
1.4671917	-6.79902	0.095	0.783	2.60984068746943e-20	Epithelial_cells	GRASP
1.9822103	-4.5878	0.149	0.913	3.52595585946885e-20	Epithelial_cells	GMFG
2.3411041	-2.3044	0.135	0.87	4.16435614341745e-20	Epithelial_cells	LCP1
2.9420248	-6.49216	0.021	0.478	5.23327388017832e-20	Epithelial_cells	TNFSF8
3.3691027	-7.80741	0.069	0.696	5.99295990388156e-20	Epithelial_cells	ADAMDEC1
3.5754153	-4.70819	0.09	0.783	6.35994877840882e-20	Epithelial_cells	OLFML2B
3.8642559	-7.96148	0.199	0.957	6.87373853828961e-20	Epithelial_cells	MSR1
4.2357913	-4.02645	0.027	0.522	7.53462570407935e-20	Epithelial_cells	TGFB1
4.8500634	-8.24263	0.202	0.957	8.62729284831922e-20	Epithelial_cells	C5AR1
4.9001263	-7.27845	0.029	0.522	8.71634474681443e-20	Epithelial_cells	AQP9
5.9128240	-7.46076	0.029	0.522	1.05177314665619e-19	Epithelial_cells	IL7R
6.2915904	-6.4418	0.011	0.391	1.11914811098609e-19	Epithelial_cells	CD33
9.8536208	-6.68701	0.003	0.304	1.75276207244572e-19	Epithelial_cells	HK3
1.3438357	-5.86694	0.003	0.304	2.39041507879569e-19	Epithelial_cells	MYO1G
1.4287277	-6.0259	0.072	0.696	2.54142088810027e-19	Epithelial_cells	LCP2
1.6392011	-4.54708	0.141	0.87	2.91581092838121e-19	Epithelial_cells	HAVCR2
1.6897859	-3.83621	0.164	0.913	3.00579121602173e-19	Epithelial_cells	MAFB
2.4293054	-4.31862	0.188	0.957	4.32124861205188e-19	Epithelial_cells	BASP1
2.6771741	-7.98943	0.061	0.652	4.76215746482583e-19	Epithelial_cells	OSM
3.0319304	-7.27522	0.024	0.478	5.3931979462151e-19	Epithelial_cells	CD48
3.6905214	-4.2588	0.04	0.565	6.5646995828502e-19	Epithelial_cells	OLR1

4.5472750	-6.92902	0.032	0.522	8.08869294507337e-19	Epithelial_cells	CSF3R
6.1954015	-7.25248	0.18	0.913	1.10203803197545e-18	Epithelial_cells	BCL2A1
8.5196307	-3.25615	0.138	0.87	1.515471920774e-18	Epithelial_cells	PECAM1
1.1082811	-7.41683	0.22	0.957	1.97141049372739e-18	Epithelial_cells	ITGB2
1.1949656	-5.58213	0.212	0.957	2.12560483434313e-18	Epithelial_cells	ARL4C
1.2157824	-4.43012	0.069	0.696	2.16263391083979e-18	Epithelial_cells	NRP2
1.3588965	-6.28799	0.09	0.739	2.41720510366268e-18	Epithelial_cells	SLC16A10
1.5839841	-7.65209	0.053	0.609	2.81759102080353e-18	Epithelial_cells	FPR1
1.5919069	-5.11439	0.09	0.739	2.83168407581481e-18	Epithelial_cells	RNASE6
1.9799299	-7.99114	0.019	0.435	3.52189933810823e-18	Epithelial_cells	DOK2
2.0413033	-7.25709	0.042	0.565	3.63107040934338e-18	Epithelial_cells	PLCB2
2.0707033	-6.13902	0.008	0.348	3.68336720005865e-18	Epithelial_cells	SLC1A3
2.2779394	-4.60973	0.125	0.826	4.05199870197184e-18	Epithelial_cells	TLR2
2.3493300	-5.19117	0.156	0.87	4.17898829942363e-18	Epithelial_cells	CD53
2.3557170	-6.29083	0.008	0.348	4.19034941715198e-18	Epithelial_cells	OSCAR
2.3837974	-4.32346	0.17	0.913	4.24029896091768e-18	Epithelial_cells	PDE4B
2.8213471	-4.30493	0.175	0.913	5.01861222216266e-18	Epithelial_cells	PLXNC1
3.4607891	-7.41403	0.013	0.391	6.15605181716966e-18	Epithelial_cells	PRAM1
4.5910831	-5.01116	0.127	0.826	8.16661865686564e-18	Epithelial_cells	RUNX3
5.8662261	-5.87696	0.276	1	1.04348430666506e-17	Epithelial_cells	AIF1
6.7553184	-5.5915	0.034	0.522	1.20163605447151e-17	Epithelial_cells	CSF2RB
6.9109723	-4.55311	0.103	0.783	1.22932375487497e-17	Epithelial_cells	SLAMF7
1.1800592	-5.10138	0.034	0.522	2.09908937308123e-17	Epithelial_cells	LY9
1.6671360	-8.60975	0.172	0.87	2.96550165788319e-17	Epithelial_cells	FCGR3A
2.0623667	-4.93311	0.143	0.826	3.66853796362628e-17	Epithelial_cells	CD84
2.5424189	-5.45932	0.056	0.609	4.52245474267476e-17	Epithelial_cells	KLHL6
2.9344207	-7.88542	0.085	0.696	5.21974769234286e-17	Epithelial_cells	MMP19
2.9810615	-3.0056	0.141	0.826	5.30271235604125e-17	Epithelial_cells	MS4A6A
3.0145239	-6.81961	0.021	0.435	5.36223528020466e-17	Epithelial_cells	KMO
3.2630249	-7.08909	0.175	0.87	5.80426882293344e-17	Epithelial_cells	CECR1
3.3527651	-6.14238	0.22	0.957	5.96389870697365e-17	Epithelial_cells	PTGS2
3.8408865	-5.08521	0.111	0.783	6.8321690636355e-17	Epithelial_cells	ARHGAP30
3.9286248	-5.70137	0.236	0.957	6.98823783450023e-17	Epithelial_cells	SIRPA
3.9394103	-3.51858	0.127	0.826	7.00742317051714e-17	Epithelial_cells	PLXND1
4.3242087	-4.67363	0.101	0.739	7.69190258122981e-17	Epithelial_cells	MYO1F
4.3819551	-6.65118	0.029	0.478	7.79462188857596e-17	Epithelial_cells	ASGR1
4.3924901	-7.90906	0.005	0.304	7.81336151056623e-17	Epithelial_cells	TMEM52B
4.4632629	-3.70001	0.066	0.652	7.93925204817406e-17	Epithelial_cells	RCSD1
5.6474633	-7.10987	0.048	0.565	1.00457078570431e-16	Epithelial_cells	TREM1
6.9634442	-3.68138	0.021	0.435	1.23865745882302e-16	Epithelial_cells	TDRD6
1.1184524	-6.63633	0.016	0.391	1.98950313559904e-16	Epithelial_cells	ARHGAP25
1.4426303	-5.91136	0.106	0.739	2.56615083181466e-16	Epithelial_cells	TNFSF13B
1.5677413	-8.29462	0.011	0.348	2.78869838842731e-16	Epithelial_cells	RETN
2.1052021	-6.17003	0.011	0.348	3.74473352311882e-16	Epithelial_cells	HTRA4
2.4761683	-5.94959	0.04	0.522	4.40460830685694e-16	Epithelial_cells	IL4I1
3.0261333	-4.28695	0.204	0.913	5.38288606901066e-16	Epithelial_cells	SLC31A2
4.3629117	-7.42061	0.024	0.435	7.76074736031504e-16	Epithelial_cells	LILRA5
4.7760539	-5.80404	0.003	0.261	8.49564480322138e-16	Epithelial_cells	F13A1
4.7760539	-6.51955	0.003	0.261	8.49564480322138e-16	Epithelial_cells	LOC101928489
5.8739210	-7.47932	0.35	1	1.04485307902623e-15	Epithelial_cells	ITGAX
6.7265858	-8.63527	0.286	0.957	1.19652509558702e-15	Epithelial_cells	SDS
6.8531921	-5.49406	0.05	0.565	1.21904581119444e-15	Epithelial_cells	ITGAM
6.9869254	-5.48211	0.064	0.609	1.24283429172796e-15	Epithelial_cells	DSE
1.0819482	-5.18609	0.064	0.609	1.92456956578238e-15	Epithelial_cells	ICAM4
1.1265439	-7.11106	0.117	0.739	2.00389633139653e-15	Epithelial_cells	HCST
1.6547654	-5.12532	0.093	0.696	2.94349679204748e-15	Epithelial_cells	FAM49A
1.7494697	-6.57745	0.042	0.522	3.11195674398565e-15	Epithelial_cells	TTYH2
1.9096737	-7.00319	0.369	1	3.39692770923802e-15	Epithelial_cells	LAPTM5

2.5224967	-4.32545	0.223	0.913	4.48701713927971e-15	Epithelial_cells	GPSM3
3.4349664	-7.3258	0.297	0.957	6.11011824992164e-15	Epithelial_cells	G0S2
3.6816508	-5.22697	0.218	0.913	6.54892060676586e-15	Epithelial_cells	EMILIN2
3.9642559	-3.99353	0.064	0.609	7.05161851322375e-15	Epithelial_cells	CLEC11A
4.4290414	-6.12213	0.027	0.435	7.87837888169562e-15	Epithelial_cells	DPEP2
4.5054688	-5.75592	0.377	1	8.01432807729879e-15	Epithelial_cells	GPNMB
4.9853001	-4.48682	0.151	0.826	8.86785182362532e-15	Epithelial_cells	PREX1
5.5412553	-6.2891	0.045	0.522	9.85678505671822e-15	Epithelial_cells	MARCH1
5.5613517	-5.29486	0.034	0.478	9.89253253148885e-15	Epithelial_cells	TM6SF1
5.6856646	-3.79799	0.164	0.87	1.01136602008246e-14	Epithelial_cells	APOBR
6.7208002	-6.92786	0.393	1	1.1954959494753e-14	Epithelial_cells	TYROBP
7.1571250	-3.46971	0.286	1	1.27310939896964e-14	Epithelial_cells	MSN
9.8756958	-3.90126	0.329	1	1.75668877628208e-14	Epithelial_cells	PMP22
1.0457295	-4.66898	0.286	1	1.86014380205521e-14	Epithelial_cells	NR4A3
1.0528052	-4.956	0.013	0.348	1.8727300471447e-14	Epithelial_cells	LINC01094
1.3775629	-4.22229	0.133	0.783	2.4504088877863e-14	Epithelial_cells	DOCK4
1.4162034	-5.19453	0.236	0.913	2.51914273793344e-14	Epithelial_cells	SLCO2B1
1.5357527	-5.19072	0.013	0.348	2.73179705989805e-14	Epithelial_cells	GIMAP6
1.8317784	-7.39033	0.037	0.478	3.25836741829235e-14	Epithelial_cells	CLEC5A
1.9322359	-4.51834	0.117	0.739	3.43706137704465e-14	Epithelial_cells	CFD
2.6345998	-4.54265	0.22	0.87	4.6864262516338e-14	Epithelial_cells	HLA-DRB6
3.9685862	-6.10655	0.424	1	7.05932120806899e-14	Epithelial_cells	FCER1G
4.9258214	-4.10199	0.146	0.783	8.76205122467278e-14	Epithelial_cells	ST3GAL5
5.3085495	-4.15739	0.048	0.522	9.44284801090477e-14	Epithelial_cells	CLEC4A
5.9290029	-3.7498	0.088	0.652	1.05465104517643e-13	Epithelial_cells	PDPN
5.9444203	-5.36137	0.329	0.957	1.05739348621189e-13	Epithelial_cells	GPR183
7.1850719	-5.08814	0.149	0.783	1.27808059754294e-13	Epithelial_cells	PTAFR
7.4642244	-3.56824	0.125	0.739	1.3277362375004e-13	Epithelial_cells	LY96
9.1280228	-4.83732	0.424	1	1.62369269601718e-13	Epithelial_cells	TNFRSF1B
1.1025271	-6.76598	0.276	0.913	1.96117527416398e-13	Epithelial_cells	CD14
1.1619665	-5.72023	0.281	0.913	2.06690606147028e-13	Epithelial_cells	HLA-DMB
1.2080186	-8.42731	0.08	0.609	2.14882353316833e-13	Epithelial_cells	VSIG4
1.2235477	-3.58589	0.326	1	2.17644672171582e-13	Epithelial_cells	KCTD12
1.2392315	-4.76277	0.074	0.609	2.20434501520158e-13	Epithelial_cells	HSD17B14
1.4620036	-6.57816	0.005	0.261	2.60061205538689e-13	Epithelial_cells	SIRPB1
1.4767662	-4.79802	0.454	1	2.6268717628307e-13	Epithelial_cells	SRGN
1.5701244	-6.75486	0.016	0.348	2.79293737123593e-13	Epithelial_cells	GNGT2
2.0343753	-4.46	0.005	0.261	3.61874681020826e-13	Epithelial_cells	HLA-DQA2
2.2297104	-4.49458	0.202	0.87	3.96620889215482e-13	Epithelial_cells	KCNAB2
2.2323825	-5.77579	0.016	0.348	3.97096211215163e-13	Epithelial_cells	GIMAP4
3.5597651	-5.12369	0.162	0.783	6.33211033655735e-13	Epithelial_cells	ADAP2
3.7523813	-6.3464	0.204	0.826	6.6747360059414e-13	Epithelial_cells	TREM2
4.2188296	-4.54623	0.05	0.522	7.50445420916221e-13	Epithelial_cells	ARHGEF6
4.9716823	-6.27173	0.011	0.304	8.84362864799973e-13	Epithelial_cells	FCGR2C
5.2246256	-5.03974	0.032	0.435	9.2935641690984e-13	Epithelial_cells	ITGA4
5.8253614	-4.2367	0.451	1	1.03621530280493e-12	Epithelial_cells	TYMP
5.8803868	-5.01874	0.424	1	1.04600322011712e-12	Epithelial_cells	TNFAIP2
6.1517373	-4.33972	0.164	0.783	1.09427103585148e-12	Epithelial_cells	WIPF1
6.3790939	-5.0365	0.024	0.391	1.13471323921295e-12	Epithelial_cells	NFKBID
9.3568564	-6.31128	0.316	0.913	1.66439761806261e-12	Epithelial_cells	CD83
1.0953821	-4.33589	0.093	0.652	1.94846585618779e-12	Epithelial_cells	PYGL
1.2026120	-5.81502	0.101	0.652	2.13920639267521e-12	Epithelial_cells	TAGAP
1.2632965	-5.42534	0.207	0.826	2.24715189688807e-12	Epithelial_cells	NPL
1.5749612	-5.82418	0.324	0.913	2.80154098258626e-12	Epithelial_cells	C1QC
2.0347642	-4.39852	0.294	0.913	3.61943869951615e-12	Epithelial_cells	PIK3AP1
2.1920859	-5.07233	0.515	1	3.89928257654286e-12	Epithelial_cells	TNFAIP3
2.3900516	-4.27286	0.233	0.87	4.25142383557005e-12	Epithelial_cells	PPP1R18
2.5738614	-3.54685	0.257	0.913	4.57838482099188e-12	Epithelial_cells	SEMA4A

2.6187740	-6.87097	0.019	0.348	4.65827525159022e-12	Epithelial_cells	SIGLEC14
2.6777888	-5.1347	0.034	0.435	4.76325088506535e-12	Epithelial_cells	GBP5
3.4776132	-6.24332	0.027	0.391	6.18597852762018e-12	Epithelial_cells	ATP8B4
3.8283689	-4.34061	0.188	0.826	6.80990272692082e-12	Epithelial_cells	ALOX5
4.5826104	-5.10016	0.249	0.87	8.15154740960039e-12	Epithelial_cells	HLA-DQB1
5.0042774	-5.27698	0.154	0.739	8.90160866240599e-12	Epithelial_cells	CD52
5.5763587	-3.22274	0.212	0.87	9.91922685991608e-12	Epithelial_cells	CEP170
5.6755126	-4.00113	0.324	0.957	1.00956019790321e-11	Epithelial_cells	ARRB2
5.7855693	-4.37044	0.019	0.348	1.0291370798235e-11	Epithelial_cells	STARD8
6.5129367	-3.77332	0.072	0.565	1.15852119648894e-11	Epithelial_cells	VASH1
6.8417866	-4.44552	0.382	0.957	1.21701701658509e-11	Epithelial_cells	ZNF331
7.6011212	-4.5001	0.408	0.957	1.35208745319808e-11	Epithelial_cells	RGS1
8.1885642	-4.2987	0.509	1	1.45658180217207e-11	Epithelial_cells	ICAM1
8.5997590	-4.21915	0.602	1	1.52972513421943e-11	Epithelial_cells	HLA-DRA
8.9524417	-6.4514	0.013	0.304	1.59246033780425e-11	Epithelial_cells	LOC101927070
9.9190720	-6.0997	0.013	0.304	1.76440453170269e-11	Epithelial_cells	DCSTAMP
9.9190720	-6.25669	0.013	0.304	1.76440453170269e-11	Epithelial_cells	S100B
1.0872921	-5.50471	0.65	1	1.93407534441693e-11	Epithelial_cells	CTSL
1.1693843	-4.69119	0.833	1	2.08010096176771e-11	Epithelial_cells	IFI30
1.2415163	-4.17454	0.207	0.826	2.20840930150704e-11	Epithelial_cells	ICOSLG
1.3060623	-4.57639	0.915	1	2.32322373177063e-11	Epithelial_cells	CTSD
1.3478874	-5.72477	0.013	0.304	2.3976221332785e-11	Epithelial_cells	GGTA1P
1.3541652	-4.13724	0.902	1	2.40878921072348e-11	Epithelial_cells	CTSB
1.3901556	-3.48087	0.188	0.826	2.47280884558503e-11	Epithelial_cells	RASSF5
1.4887705	-3.61467	0.939	1	2.64822505211332e-11	Epithelial_cells	PSAP
1.5057836	-5.49419	0.008	0.261	2.67848802222811e-11	Epithelial_cells	TNFAIP8L2
1.8854167	-4.44409	0.061	0.522	3.35377935239049e-11	Epithelial_cells	MMP9
1.9431395	-7.65023	0.377	0.913	3.45645670873589e-11	Epithelial_cells	C1QB
1.9523211	-6.64916	0.029	0.391	3.47278892864425e-11	Epithelial_cells	SASH3
1.9553692	-3.80246	0.878	1	3.47821087535608e-11	Epithelial_cells	PLAUR
2.3846540	-7.24011	0.167	0.739	4.24182255897468e-11	Epithelial_cells	TNF
2.6591421	-6.75992	0.374	0.913	4.73008196959759e-11	Epithelial_cells	C1QA
2.9305895	-3.42485	0.149	0.739	5.21293267465876e-11	Epithelial_cells	DPYD
3.0778975	-5.77203	0.021	0.348	5.47496421249948e-11	Epithelial_cells	GPR65
3.8195477	-5.06887	0.029	0.391	6.79421150397551e-11	Epithelial_cells	MILR1
4.1465308	-3.2516	0.851	1	7.37584901132491e-11	Epithelial_cells	NFKBIA
4.3052997	-3.97733	0.064	0.522	7.6582671075911e-11	Epithelial_cells	CD36
4.3665416	-6.16538	0.021	0.348	7.76720432951797e-11	Epithelial_cells	HS3ST2
5.6986852	-2.76879	0.058	0.522	1.01368212873929e-10	Epithelial_cells	TRPV4
7.5007589	-8.48274	0.252	0.826	1.33423500693143e-10	Epithelial_cells	CCL4
7.7918552	-4.15446	0.268	0.87	1.38601521613208e-10	Epithelial_cells	CXCR4
9.5835941	-5.08514	0.064	0.522	1.70472972424345e-10	Epithelial_cells	AMPD3
1.4143796	-4.95262	0.3	0.87	2.51589858271683e-10	Epithelial_cells	ACP5
1.4639842	-5.34377	0.032	0.391	2.60413514737488e-10	Epithelial_cells	UBASH3B
1.5022936	-5.82549	0.512	0.957	2.67227998987522e-10	Epithelial_cells	LIPA
1.5699916	-3.30186	0.199	0.826	2.79270114937577e-10	Epithelial_cells	FAM20C
1.6874573	-2.32818	0.997	1	3.0016491396651e-10	Epithelial_cells	FTL
1.7173221	-4.33603	0.13	0.696	3.05477261882869e-10	Epithelial_cells	SLC23A2
1.7181320	-3.41191	0.578	1	3.05621333464316e-10	Epithelial_cells	VIM
1.8518438	-2.82945	0.26	0.913	3.29405980174951e-10	Epithelial_cells	FNBP1
1.8735223	-3.71819	0.324	0.913	3.33262159898001e-10	Epithelial_cells	QKI
1.9273289	-2.91196	0.775	1	3.42833274565457e-10	Epithelial_cells	LIMS1
2.1139516	-3.25633	0.78	1	3.76029719520019e-10	Epithelial_cells	CD74
2.2994320	-6.43083	0.024	0.348	4.09022965181821e-10	Epithelial_cells	ATP6V0D2
2.3310151	-2.85665	0.271	0.913	4.14640982544863e-10	Epithelial_cells	EMP3
2.3384611	-3.10634	0.385	1	4.15965475539034e-10	Epithelial_cells	PLXDC2
2.3811752	-3.66187	0.281	0.87	4.23563449663796e-10	Epithelial_cells	DOCK8
2.7112624	-5.63889	0.024	0.348	4.8227936790975e-10	Epithelial_cells	ADPRH

2.9754709	-4.01956	0.101	0.609	5.29276768900474e-10	Epithelial_cells	CMTM3
3.0274353	-3.10875	0.042	0.435	5.38520205679072e-10	Epithelial_cells	FLVCR2
3.3306180	-3.44557	0.581	1	5.92450338723911e-10	Epithelial_cells	METRNL
3.3735871	-4.48651	0.069	0.522	6.00093687490705e-10	Epithelial_cells	CHST11
3.4143804	-3.19318	0.472	0.957	6.07349988429005e-10	Epithelial_cells	RAB31
3.4490946	-3.78055	0.589	0.957	6.13524948236775e-10	Epithelial_cells	CD68
3.7630755	-3.41919	0.538	1	6.69375880237985e-10	Epithelial_cells	RNF130
4.2788443	-6.70577	0.045	0.435	7.61120826323854e-10	Epithelial_cells	LILRB3
4.3170658	-4.63853	0.207	0.783	7.67919669459063e-10	Epithelial_cells	SPHK1
4.4995394	-2.95235	0.599	1	8.00378084998474e-10	Epithelial_cells	COTL1
4.5465865	-2.55778	0.894	1	8.08746815259071e-10	Epithelial_cells	LITAF
4.8092619	-4.32941	0.024	0.348	8.55471510123449e-10	Epithelial_cells	FLI1
5.1649334	-4.50689	0.191	0.783	9.18738358237102e-10	Epithelial_cells	DDX3Y
6.4480951	-6.72402	0.448	0.913	1.14698716051095e-09	Epithelial_cells	IL1B
6.4686349	-3.37221	0.459	1	1.15064079082202e-09	Epithelial_cells	CCDC88A
7.0033953	-2.76874	0.735	1	1.24576396806635e-09	Epithelial_cells	TTYH3
7.2215418	-3.29297	0.18	0.783	1.28456785876784e-09	Epithelial_cells	CLEC2B
7.3351225	-2.77581	0.111	0.652	1.30477159890244e-09	Epithelial_cells	MEF2C
8.0113798	-2.95761	0.353	0.957	1.42506425546273e-09	Epithelial_cells	SLC16A3
8.2881675	-2.84983	0.52	1	1.47429924670374e-09	Epithelial_cells	LRP1
9.4062114	-3.31942	0.804	1	1.67317689042611e-09	Epithelial_cells	SOD2
1.0164211	-4.77545	0.034	0.391	1.80800986016895e-09	Epithelial_cells	SLC16A6
1.0337628	-2.21725	0.995	1	1.83885744122942e-09	Epithelial_cells	FTH1
1.0614259	-4.92603	0.106	0.609	1.88806444143416e-09	Epithelial_cells	RIN3
1.1301401	-3.45506	0.122	0.652	2.01029322843036e-09	Epithelial_cells	PLEKHO1
1.1989430	-4.67658	0.467	0.957	2.13267981608651e-09	Epithelial_cells	APOC1
1.3276154	-2.50673	0.775	1	2.36156229670238e-09	Epithelial_cells	GRB2
1.5861027	-4.05256	0.432	0.957	2.82135950217255e-09	Epithelial_cells	GK
1.5898272	-4.08574	0.257	0.826	2.82798463046862e-09	Epithelial_cells	SH2B3
1.6184828	-4.37196	0.446	0.913	2.87895726967175e-09	Epithelial_cells	GM2A
1.6272387	-4.08473	0.109	0.609	2.89453227521392e-09	Epithelial_cells	CD93
1.6608425	-2.80936	0.106	0.609	2.95430676311969e-09	Epithelial_cells	GAS7
1.7131034	-5.73021	0.019	0.304	3.04726839417954e-09	Epithelial_cells	CD300LB
1.7200036	-3.85512	0.785	0.957	3.05954256891907e-09	Epithelial_cells	ASAH1
2.0191593	-4.98952	0.308	0.87	3.59168069354752e-09	Epithelial_cells	THBD
2.4280160	-3.06432	0.645	1	4.31895497847162e-09	Epithelial_cells	CD81
2.8286624	-4.42068	0.419	0.913	5.03162468241166e-09	Epithelial_cells	ABL2
3.0174835	-4.70269	0.019	0.304	5.36749976774616e-09	Epithelial_cells	L3MBTL4-AS1
3.1199441	-5.18902	0.037	0.391	5.5497567078164e-09	Epithelial_cells	SFMBT2
3.4967325	-4.57761	0.613	1	6.21998777301992e-09	Epithelial_cells	CXCL8
3.8169047	-3.53044	0.7	0.957	6.78951022781478e-09	Epithelial_cells	PPT1
4.1666315	-2.98044	0.61	0.957	7.4116041748046e-09	Epithelial_cells	CREM
4.2570965	-4.30546	0.048	0.435	7.57252342527882e-09	Epithelial_cells	FCGR2B
4.3788472	-3.85895	0.631	0.957	7.78909349438559e-09	Epithelial_cells	TGFBI
4.6537827	-3.74548	0.111	0.609	8.27814879440703e-09	Epithelial_cells	PTGS1
4.7720418	-3.903	0.589	0.957	8.48850811718949e-09	Epithelial_cells	TIMP2
5.0523933	-4.1939	0.239	0.783	8.98719735634328e-09	Epithelial_cells	GPR137B
5.0700479	-9.5179	0.329	0.826	9.01860130996718e-09	Epithelial_cells	CCL3
5.2654694	-3.44374	0.286	0.826	9.36621712279366e-09	Epithelial_cells	PLAU
5.2926052	-3.38387	0.027	0.348	9.41448627692156e-09	Epithelial_cells	PHACTR1
5.6355026	-4.08444	0.09	0.565	1.00244320334551e-08	Epithelial_cells	OGFRL1
5.7716161	-4.17157	0.401	0.87	1.02665508144285e-08	Epithelial_cells	HLA-DPA1
6.3457696	-3.22984	0.796	1	1.1287855135216e-08	Epithelial_cells	SGK1
6.3968468	-4.45068	0.204	0.739	1.13787112158054e-08	Epithelial_cells	MERTK
7.1585805	-2.41869	0.549	0.957	1.27336831253811e-08	Epithelial_cells	HLA-DMA
7.3858395	-3.47035	0.629	1	1.31379314451775e-08	Epithelial_cells	NR4A2
7.7904969	-4.06147	0.037	0.391	1.38577359193057e-08	Epithelial_cells	SELPLG
7.8783854	-3.55094	0.061	0.478	1.40140719562992e-08	Epithelial_cells	MCTP1

7.9090429	-3.61571	0.082	0.522	1.40686055339661e-08	Epithelial_cells	SIGLEC10
7.9149440	-2.41537	0.883	1	1.40791024495123e-08	Epithelial_cells	NPC2
8.4261350	-4.81608	0.077	0.522	1.49884090806039e-08	Epithelial_cells	CHST15
9.5171168	-5.4465	0.029	0.348	1.69290475061929e-08	Epithelial_cells	P2RX7
9.5428772	-4.23195	0.117	0.609	1.69748699835734e-08	Epithelial_cells	TNFSF12
9.6792920	-3.2867	0.594	0.957	1.72175246140876e-08	Epithelial_cells	GNA13
9.9377551	-4.6039	0.141	0.652	1.76772789194458e-08	Epithelial_cells	EVI2A
1.0927255	-4.06856	0.395	0.87	1.94374022825918e-08	Epithelial_cells	SATB1
1.1063046	-4.00191	0.538	0.913	1.96789478028096e-08	Epithelial_cells	HLA-DPB1
1.1200315	-3.4976	0.133	0.652	1.99231205686853e-08	Epithelial_cells	GLIPR2
1.1722459	-6.66483	0.013	0.261	2.08519107647765e-08	Epithelial_cells	MEIKIN
1.2292521	-5.30435	0.013	0.261	2.18659376261421e-08	Epithelial_cells	CXorf21
1.2292521	-5.23909	0.013	0.261	2.18659376261421e-08	Epithelial_cells	RGS18
1.2345502	-2.10606	0.899	1	2.1960179537922e-08	Epithelial_cells	CTSS
1.2638676	-2.67478	0.907	0.957	2.24816784916036e-08	Epithelial_cells	GRN
1.2939071	-3.05295	0.602	0.957	2.30160207994004e-08	Epithelial_cells	HLA-DRB1
1.2971323	-2.22704	0.865	1	2.30733895443101e-08	Epithelial_cells	GLUL
1.3695080	-3.45745	0.162	0.696	2.43608089122134e-08	Epithelial_cells	CD109
1.4061756	-6.83571	0.021	0.304	2.50130523141684e-08	Epithelial_cells	LOC101927243
1.4646350	-6.11045	0.021	0.304	2.60529278744169e-08	Epithelial_cells	WDR17
1.4927371	-3.49201	0.668	0.957	2.65528076618949e-08	Epithelial_cells	SERPINB9
1.5254770	-6.95125	0.021	0.304	2.71351855352152e-08	Epithelial_cells	CLEC4E
1.5267556	-2.71605	0.846	1	2.71579291302996e-08	Epithelial_cells	NAMPT
1.5575656	-3.76278	0.064	0.478	2.77059781103971e-08	Epithelial_cells	RGL1
1.6900181	-4.81899	0.172	0.696	3.00620428580325e-08	Epithelial_cells	LPXN
2.3333119	-1.9752	0.615	1	4.15049528925248e-08	Epithelial_cells	LGALS1
2.5397892	-4.55498	0.769	0.957	4.51777708860115e-08	Epithelial_cells	LGMN
2.6438346	-2.88873	0.387	0.913	4.70285302713678e-08	Epithelial_cells	PDE4A
2.6765732	-3.05608	0.599	1	4.76108849804773e-08	Epithelial_cells	RAPGEF1
2.7610463	-8.17524	0.156	0.652	4.91134922845308e-08	Epithelial_cells	ALOX5AP
3.0326585	-4.60375	0.021	0.304	5.39449310961045e-08	Epithelial_cells	CMKLR1
3.1160866	-3.88409	0.141	0.652	5.54289498329985e-08	Epithelial_cells	TRPV2
3.4103888	-2.60269	0.886	1	6.06639960720339e-08	Epithelial_cells	IER3
3.5016977	-5.89125	0.13	0.609	6.22882004033651e-08	Epithelial_cells	HLA-DOA
3.7957225	-1.47334	0.95	1	6.75183130995842e-08	Epithelial_cells	OAZ1
3.8323934	-4.47709	0.398	0.87	6.81706154641437e-08	Epithelial_cells	DUSP2
3.8699646	-2.80702	0.78	1	6.88389320780366e-08	Epithelial_cells	ANXA5
3.8975535	-2.50436	0.82	1	6.93296819495114e-08	Epithelial_cells	GPX1
3.9391370	-2.8292	0.247	0.783	7.00693706828014e-08	Epithelial_cells	GAA
4.0541406	-3.27043	0.546	0.913	7.21150534181299e-08	Epithelial_cells	TPP1
4.1319164	-4.94674	0.289	0.783	7.34985303902529e-08	Epithelial_cells	GNB4
4.1780172	-4.19225	0.109	0.565	7.43185708215511e-08	Epithelial_cells	GPR34
4.2019869	-4.40832	0.276	0.783	7.47449439462996e-08	Epithelial_cells	NRP1
4.6440596	-3.72011	0.485	0.913	8.26085335982921e-08	Epithelial_cells	PTPRE
4.8853725	-5.70841	0.032	0.348	8.69010070583742e-08	Epithelial_cells	RAB3IL1
4.9560423	-3.99256	0.406	0.87	8.81580810829356e-08	Epithelial_cells	ARHGDIB
5.2798248	3.773128	0.971	1	9.39175239198784e-08	Epithelial_cells	KRT8
6.4921887	-4.04356	0.127	0.609	1.15483052651725e-07	Epithelial_cells	SNN
6.9023465	-3.42284	0.207	0.739	1.22778939866655e-07	Epithelial_cells	SAMHD1
7.0862997	-7.46111	0.045	0.391	1.26051100671047e-07	Epithelial_cells	FOLR2
7.9012377	3.612584	0.963	1	1.4054721721902e-07	Epithelial_cells	KRT18
9.3770337	-3.54139	0.462	0.913	1.66798676738315e-07	Epithelial_cells	PFKFB3
9.7851898	-3.05101	0.703	0.957	1.74058957625495e-07	Epithelial_cells	CTSC
1.0153413	-5.87748	0.483	0.957	1.80608920322674e-07	Epithelial_cells	SPP1
1.2552979	-6.16253	0.016	0.261	2.23292407947557e-07	Epithelial_cells	PRKCB
1.2902118	3.943603	0.963	1	2.29502884046584e-07	Epithelial_cells	ELF3
1.2904821	-2.38468	0.793	0.957	2.29550962943038e-07	Epithelial_cells	CAPG
1.3242554	3.717428	0.968	1	2.35558558373736e-07	Epithelial_cells	FXYD3

1.3290053	-3.77268	0.517	0.913	2.36403463857303e-07	Epithelial_cells	PLIN2
1.3667229	-3.83864	0.318	0.826	2.43112681627772e-07	Epithelial_cells	NFKBIE
1.3692238	-5.87247	0.016	0.261	2.43557537606516e-07	Epithelial_cells	TLR1
1.4619375	-7.89064	0.387	0.826	2.60049459332846e-07	Epithelial_cells	APOE
1.4709671	-3.87156	0.39	0.87	2.61655642102744e-07	Epithelial_cells	DAB2
1.4932576	-5.67166	0.016	0.261	2.65620673186359e-07	Epithelial_cells	LOC101927131
1.5233809	-2.75317	0.172	0.696	2.70979002623001e-07	Epithelial_cells	GAB2
1.5304919	-3.73076	0.485	0.87	2.72243916030072e-07	Epithelial_cells	SCPEP1
1.5593358	-4.83333	0.016	0.261	2.77374657475702e-07	Epithelial_cells	CD1D
1.5692258	-3.98376	0.024	0.304	2.79133898950563e-07	Epithelial_cells	C16orf54
1.5779122	-3.02773	0.698	0.957	2.80679033000027e-07	Epithelial_cells	GNAI2
1.7653978	4.03149	0.966	0.957	3.14028973980881e-07	Epithelial_cells	MUC1
2.1559960	-4.23425	0.045	0.391	3.83508583024647e-07	Epithelial_cells	RHOH
2.3708705	-3.41025	0.106	0.565	4.21730450637069e-07	Epithelial_cells	RASGRP3
2.4308328	-4.81407	0.093	0.522	4.32396542038357e-07	Epithelial_cells	BRE-AS1
2.5454267	-2.68714	0.419	0.957	4.52780513250833e-07	Epithelial_cells	CCL20
2.6481280	-3.08057	0.268	0.826	4.71049022338435e-07	Epithelial_cells	PLEKHG2
2.6717553	-2.57429	0.056	0.435	4.75251841749741e-07	Epithelial_cells	GLIPR1
2.7094350	-2.64226	0.666	0.957	4.81954310760798e-07	Epithelial_cells	GNS
2.7160861	-2.01522	0.095	0.565	4.83137405395224e-07	Epithelial_cells	SCARF1
2.7403875	-4.54827	0.058	0.435	4.87460132017292e-07	Epithelial_cells	IFFO1
3.1775610	-4.7242	0.048	0.391	5.65224568226584e-07	Epithelial_cells	VAV1
3.3771542	-2.4784	0.016	0.261	6.00728204591788e-07	Epithelial_cells	FLRT2
3.3940886	-3.98111	0.034	0.348	6.03740497743731e-07	Epithelial_cells	CACNA2D4
3.4328763	-2.06594	0.873	1	6.10640050037941e-07	Epithelial_cells	ATP6V0C
3.4548005	-3.02778	0.69	0.957	6.14539922248557e-07	Epithelial_cells	HEXA
3.4792980	3.718998	0.979	1	6.18897543596858e-07	Epithelial_cells	EPCAM
3.5155023	-2.0273	0.597	1	6.25337563236005e-07	Epithelial_cells	RNF13
3.7578980	2.768843	0.939	0.957	6.68454913384559e-07	Epithelial_cells	DSTN
3.9880721	4.808189	0.886	0.391	7.09398267726929e-07	Epithelial_cells	DSP
4.4547167	-3.58644	0.111	0.565	7.92405010369446e-07	Epithelial_cells	GYPC
4.4600177	-2.91082	0.727	0.913	7.9334795219923e-07	Epithelial_cells	MGAT1
4.6083470	-2.37922	0.088	0.522	8.19732766573215e-07	Epithelial_cells	FMNL3
4.6088447	-3.09004	0.26	0.783	8.19821309376966e-07	Epithelial_cells	KYNU
4.8570868	-3.28415	0.244	0.783	8.63978611153222e-07	Epithelial_cells	RASGEF1B
5.0537300	-2.62966	0.562	0.957	8.98957497938112e-07	Epithelial_cells	CXCL2
5.2998591	-3.57254	0.568	0.957	9.42738949723764e-07	Epithelial_cells	RGS2
5.5762958	-3.60706	0.074	0.478	9.91911500239432e-07	Epithelial_cells	ARHGAP15
5.7603440	-3.15801	0.475	0.913	1.02464999606849e-06	Epithelial_cells	NINJ1
6.0358065	4.779249	0.859	0.261	1.07364927289615e-06	Epithelial_cells	MYO6
6.1003544	-2.03938	0.96	1	1.0851310543074e-06	Epithelial_cells	SAT1
6.3495096	-6.8385	0.064	0.435	1.12945077780436e-06	Epithelial_cells	IL23A
6.4038565	-3.44075	0.257	0.783	1.13911800468691e-06	Epithelial_cells	NIN
6.9747993	-3.57304	0.281	0.783	1.24067729994842e-06	Epithelial_cells	SLC43A3
7.0382772	-5.13597	0.077	0.478	1.2519687528086e-06	Epithelial_cells	FGR
7.8333493	-3.17193	0.626	0.913	1.3933961799876e-06	Epithelial_cells	MFSD1
7.9958840	-4.40798	0.037	0.348	1.42230785972006e-06	Epithelial_cells	AOAH
8.3333073	-3.7287	0.249	0.739	1.48232871800012e-06	Epithelial_cells	TCN2
8.9104075	-2.80631	0.796	1	1.58498329020633e-06	Epithelial_cells	CEBPB
8.9446432	-3.67536	0.332	0.826	1.59107313445643e-06	Epithelial_cells	SDSL
9.1899517	-2.15516	0.817	1	1.63470862225882e-06	Epithelial_cells	ATP1B3
9.6274602	-3.04734	0.347	0.87	1.71253262267018e-06	Epithelial_cells	ARAP1
1.0205038	-2.75229	0.31	0.826	1.81527227899888e-06	Epithelial_cells	ENG
1.0218541	-2.4554	0.814	1	1.81767418921825e-06	Epithelial_cells	PNRC1
1.0330221	-2.3151	0.716	1	1.83753974972 72e-06	Epithelial_cells	PABPC4
1.1964743	-1.93812	0.881	1	2.1282885650877e-06	Epithelial_cells	CFLAR
1.3203049	-2.99775	0.682	0.913	2.34855846122292e-06	Epithelial_cells	CREG1
1.4095497	-3.38307	0.125	0.565	2.50730708881731e-06	Epithelial_cells	HMOX1

1.4357605	-1.63995	0.844	1	2.55393094776121e-06	Epithelial_cells	ATP6V1F
1.5848725	-1.56152	0.406	0.957	2.81917125246521e-06	Epithelial_cells	SEC14L1
1.6053206	3.299738	0.947	0.826	2.85554433391519e-06	Epithelial_cells	TPM1
1.6931942	-2.03593	0.764	1	3.01185389660503e-06	Epithelial_cells	FCGRT
1.7695328	-5.83369	0.019	0.261	3.14764500076326e-06	Epithelial_cells	LILRA3
1.8715051	-3.77776	0.196	0.696	3.32903339348232e-06	Epithelial_cells	CSGALNACT2
1.9039760	-3.79213	0.369	0.826	3.38679261181698e-06	Epithelial_cells	DMXL2
2.1512670	-2.58281	0.467	0.913	3.8266739129638e-06	Epithelial_cells	IGF2R
2.1547664	-3.1466	0.371	0.87	3.83289862469434e-06	Epithelial_cells	SOAT1
2.1563212	-4.68211	0.04	0.348	3.83566416526089e-06	Epithelial_cells	FAM78A
2.1701874	-2.50063	0.607	0.957	3.86032940034688e-06	Epithelial_cells	MYO9B
2.2944516	4.236991	0.926	0.826	4.08137053503987e-06	Epithelial_cells	KLF5
2.2951901	-1.72976	0.767	0.957	4.08268419012046e-06	Epithelial_cells	LAMP1
2.3621141	-5.6143	0.029	0.304	4.20172866660122e-06	Epithelial_cells	C10orf128
2.3621141	-5.26508	0.029	0.304	4.20172866660122e-06	Epithelial_cells	DPH1
2.4241723	-1.49284	0.019	0.261	4.3121177340662e-06	Epithelial_cells	COL8A2
2.5240463	3.976912	0.928	0.739	4.48977360070262e-06	Epithelial_cells	FHL2
2.5969673	-3.44467	0.08	0.478	4.61948548472804e-06	Epithelial_cells	BIN2
2.6627322	-4.35411	0.218	0.696	4.7364681861154e-06	Epithelial_cells	RAC2
2.8306562	3.261777	0.936	0.957	5.03517136753283e-06	Epithelial_cells	NQO1
2.9652436	3.332894	0.923	0.783	5.27457537804668e-06	Epithelial_cells	SMIM22
3.1353739	-2.69374	0.493	0.913	5.57720313147512e-06	Epithelial_cells	PRNP
3.2557990	-2.92684	0.202	0.696	5.79141532856746e-06	Epithelial_cells	NR3C1
3.7140292	-2.04485	0.488	0.957	6.60651514908108e-06	Epithelial_cells	AKR1B1
4.3604270	-7.85314	0.114	0.522	7.75632762909249e-06	Epithelial_cells	CCL18
4.8563332	-3.94821	0.366	0.783	8.6384454991418e-06	Epithelial_cells	S100A9
4.8612408	2.590844	0.966	1	8.64717521498033e-06	Epithelial_cells	IFI27
4.8914222	-1.79225	0.117	0.565	8.70086186300004e-06	Epithelial_cells	A2M
4.9343543	-2.3026	0.239	0.739	8.77722954664295e-06	Epithelial_cells	TMEM106A
4.9421742	-3.9093	0.286	0.783	8.79113957988381e-06	Epithelial_cells	CCRL2
4.9450692	-2.46263	0.178	0.652	8.7962891339349e-06	Epithelial_cells	GNA15
5.1132439	-3.92428	0.263	0.739	9.09543828215585e-06	Epithelial_cells	THEMIS2
5.4597825	-3.38547	0.393	0.826	9.71186117637643e-06	Epithelial_cells	RGS10
5.5774063	-2.78371	0.403	0.87	9.92109039760386e-06	Epithelial_cells	RHOG
5.9788119	-3.34753	0.565	0.87	1.06351106780689e-05	Epithelial_cells	AP1B1
6.0213437	-3.61888	0.066	0.435	1.07107661883729e-05	Epithelial_cells	FCMR
6.1718614	-2.07581	0.053	0.391	1.09785072055034e-05	Epithelial_cells	FAM26F
6.4116465	-2.84099	0.3	0.783	1.14050369515107e-05	Epithelial_cells	PI4K2A
6.5602227	-3.3928	0.401	0.826	1.16693241715247e-05	Epithelial_cells	ST3GAL1
6.9457354	3.528556	0.881	0.478	1.2355074246706e-05	Epithelial_cells	JUP
7.0028306	-4.09453	0.401	0.826	1.24566351030503e-05	Epithelial_cells	UCP2
7.1907258	-2.8072	0.61	0.913	1.27908632277062e-05	Epithelial_cells	LYN
7.1979321	-3.91986	0.069	0.435	1.28036816258493e-05	Epithelial_cells	DFNA5
7.5212780	-1.72707	0.029	0.304	1.33788494633702e-05	Epithelial_cells	TIAM1
7.5903350	-2.04645	0.151	0.609	1.35016879717471e-05	Epithelial_cells	MAF
7.6102660	-2.57761	0.66	0.957	1.35371411779746e-05	Epithelial_cells	REL
8.2256021	-3.36222	0.438	0.826	1.46317011301202e-05	Epithelial_cells	SLC7A7
8.2604907	1.396955	0.979	1	1.46937609333734e-05	Epithelial_cells	UBB
8.4049411	4.553936	0.91	0.783	1.49507092878343e-05	Epithelial_cells	TMC5
1.0104661	-1.7982	0.83	1	1.79741713915879e-05	Epithelial_cells	CTNNB1
1.0191851	2.066906	0.995	1	1.81292663189012e-05	Epithelial_cells	S100A6
1.0237404	-2.59223	0.191	0.652	1.8210294302204e-05	Epithelial_cells	ITGA5
1.0648067	4.951502	0.761	0.043	1.89407832518842e-05	Epithelial_cells	MAGI1
1.0872613	-3.11748	0.565	0.913	1.93402047709179e-05	Epithelial_cells	MICAL1
1.1744721	-2.87048	0.682	0.913	2.08915109897175e-05	Epithelial_cells	TFRC
1.1801210	-2.80986	0.393	0.826	2.09919931374242e-05	Epithelial_cells	SLC7A8
1.1888543	3.771052	0.878	0.522	2.11473413393526e-05	Epithelial_cells	STARD10
1.1904311	-3.61015	0.332	0.826	2.11753893256057e-05	Epithelial_cells	RILPL2

1.2408060	-3.00128	0.531	0.87	2.2071458674644e-05	Epithelial_cells	STX4
1.2890252	-3.74222	0.194	0.652	2.29291805260865e-05	Epithelial_cells	FMNL1
1.2953036	-2.48583	0.626	1	2.30408616413991e-05	Epithelial_cells	CXCL3
1.2972497	-3.40819	0.271	0.739	2.30754778993905e-05	Epithelial_cells	MFSD12
1.3434847	3.725864	0.905	0.652	2.38979059615718e-05	Epithelial_cells	EMP2
1.3550343	-4.39544	0.218	0.652	2.41033510919624e-05	Epithelial_cells	MALT1
1.3839740	-5.02462	0.045	0.348	2.46181305667847e-05	Epithelial_cells	IL18BP
1.5997678	-1.88636	0.748	0.957	2.84566705599138e-05	Epithelial_cells	SCARB2
1.7171276	-2.13643	0.809	0.957	3.05442659452076e-05	Epithelial_cells	BRI3
1.7497064	-2.14641	0.448	0.87	3.11237783220987e-05	Epithelial_cells	PRCP
1.8118806	-1.88358	0.817	0.957	3.22297327497682e-05	Epithelial_cells	GRINA
1.8536252	-4.07103	0.178	0.652	3.29722860385576e-05	Epithelial_cells	RNF166
1.9706776	-5.06995	0.117	0.522	3.50544138898293e-05	Epithelial_cells	SLC15A3
2.0281966	-2.32824	0.668	0.957	3.60775624417269e-05	Epithelial_cells	ATP13A3
2.0838772	3.785854	0.889	0.696	3.70680089170296e-05	Epithelial_cells	PRSS8
2.2394309	-3.10073	0.329	0.826	3.98349984628039e-05	Epithelial_cells	TK2
2.3338637	3.023265	0.971	1	4.1514768776313e-05	Epithelial_cells	S100P
2.3582033	-2.74709	0.43	0.87	4.19477214141e-05	Epithelial_cells	CPVL
2.3851911	3.456844	0.955	1	4.24277797419232e-05	Epithelial_cells	KRT19
2.4036802	-3.06986	0.446	0.826	4.27566643890563e-05	Epithelial_cells	ABCA1
2.4209117	-3.04206	0.623	0.913	4.30631781733203e-05	Epithelial_cells	IFNGR1
2.4599954	-4.95592	0.191	0.652	4.37583995927394e-05	Epithelial_cells	CTSZ
2.4702693	2.963052	0.968	1	4.39411513846826e-05	Epithelial_cells	TSPAN8
2.6087239	-1.9116	0.546	0.87	4.64039821175892e-05	Epithelial_cells	CORO1C
2.6107930	3.50437	0.928	0.87	4.64407865868513e-05	Epithelial_cells	MAL2
2.7099681	-3.44421	0.17	0.609	4.82049134525397e-05	Epithelial_cells	SDC2
2.7354326	-4.13426	0.228	0.696	4.86578759761898e-05	Epithelial_cells	ADAM8
2.9772896	3.418436	0.889	0.783	5.29600279012693e-05	Epithelial_cells	SLC44A4
3.0464547	-2.80694	0.491	0.913	5.41903375728626e-05	Epithelial_cells	EDEM1
3.1830576	-2.4627	0.69	0.957	5.66202302732467e-05	Epithelial_cells	BNIP3L
3.1993152	2.228893	0.947	0.957	5.69094191037763e-05	Epithelial_cells	SPINT2
3.2583521	3.724716	0.891	0.609	5.79595675496172e-05	Epithelial_cells	DSG2
3.3167978	-4.65343	0.024	0.261	5.89992000604163e-05	Epithelial_cells	PTPRO
3.3609306	-3.40509	0.247	0.696	5.97842352141739e-05	Epithelial_cells	RASSF4
3.4140864	-6.83111	0.048	0.348	6.07297693478836e-05	Epithelial_cells	IL1A
3.4265313	-1.54273	0.825	1	6.09511396966262e-05	Epithelial_cells	FXYD5
3.6721268	-3.53845	0.202	0.652	6.53197916766747e-05	Epithelial_cells	C1orf54
4.0287327	-3.31888	0.568	0.87	7.16630980331329e-05	Epithelial_cells	ATP6V1B2
4.1039029	-2.19195	0.634	0.957	7.30002257245209e-05	Epithelial_cells	KDM6B
4.1286940	3.730715	0.865	0.739	7.34412091304312e-05	Epithelial_cells	TSPAN13
4.2202795	4.00279	0.841	0.435	7.50703318536083e-05	Epithelial_cells	ERBB3
4.2806157	3.508181	0.844	0.261	7.61435932720928e-05	Epithelial_cells	ST6GALNAC1
4.5392953	3.554164	0.958	0.957	8.07449862641289e-05	Epithelial_cells	LGALS4
4.6796561	-3.95945	0.143	0.565	8.32417240632671e-05	Epithelial_cells	PLBD2
5.0827254	1.545495	0.987	1	9.0411519564622e-05	Epithelial_cells	RPL18A
5.1155310	-2.4348	0.618	0.913	9.09950669293357e-05	Epithelial_cells	ZYX
5.2963650	-4.76122	0.082	0.435	9.42117418163777e-05	Epithelial_cells	S100A8
5.4414307	3.108709	0.894	0.609	9.67921703297273e-05	Epithelial_cells	PERP
5.7032329	-3.43696	0.119	0.522	0.000101449	Epithelial_cells	PPM1M
5.8925007	4.735982	0.923	0.913	0.000104816	Epithelial_cells	TACSTD2
5.9000380	3.955257	0.801	0.261	0.00010495	Epithelial_cells	GNG12
6.6482578	-1.77822	0.586	0.957	0.000118259	Epithelial_cells	BST2
6.7038239	-1.2026	0.833	1	0.000119248	Epithelial_cells	RNF149
6.8390696	-2.45114	0.692	0.913	0.000121653	Epithelial_cells	SLC31A1
6.8698662	-3.19661	0.231	0.696	0.000122201	Epithelial_cells	SLC36A1
6.9328981	-2.88262	0.424	0.826	0.000123322	Epithelial_cells	PTPN6
7.7456652	-3.09545	0.393	0.826	0.00013778	Epithelial_cells	AGAP3
8.2706509	4.853808	0.833	0.391	0.000147118	Epithelial_cells	MLPH

8.3223981	3.1671	0.897	0.652	0.000148039	Epithelial_cells	RAB25
8.5755089	3.823675	0.894	0.913	0.000152541	Epithelial_cells	EFNA1
8.9800371	-2.13973	0.859	1	0.000159737	Epithelial_cells	BTG1
9.0382275	1.389067	0.979	1	0.000160772	Epithelial_cells	RPL18
9.2369173	2.78138	0.971	1	0.000164306	Epithelial_cells	HSPB1
9.5767061	3.395436	0.87	0.696	0.00017035	Epithelial_cells	CTTN
9.7805576	3.48011	0.82	0.348	0.000173977	Epithelial_cells	NET1
9.9522746	-2.17279	0.43	0.87	0.000177031	Epithelial_cells	IRF8
1.0360342	-3.95957	0.037	0.304	0.00018429	Epithelial_cells	HGF
1.0882136	3.157938	0.963	1	0.000193571	Epithelial_cells	GPX2
1.0883108	-1.06613	0.257	0.739	0.000193589	Epithelial_cells	SERPING1
1.0914614	-3.5929	0.064	0.391	0.000194149	Epithelial_cells	PTPN22
1.2097837	-4.04494	0.05	0.348	0.000215196	Epithelial_cells	RFTN1
1.2180839	4.007068	0.817	0.348	0.000216673	Epithelial_cells	SDC1
1.2374395	3.491694	0.825	0.391	0.000220116	Epithelial_cells	LLGL2
1.2451594	2.624805	0.923	0.913	0.000221489	Epithelial_cells	S100A16
1.3040998	-4.10083	0.05	0.348	0.000231973	Epithelial_cells	CELF6
1.3290292	-2.69712	0.082	0.435	0.000236408	Epithelial_cells	FXYD6
1.5061723	-2.92023	0.17	0.609	0.000267918	Epithelial_cells	FRMD4A
1.5127633	4.411552	0.92	0.913	0.00026909	Epithelial_cells	MUC13
1.6195091	-2.71101	0.154	0.565	0.000288078	Epithelial_cells	AXL
1.6672610	-2.82021	0.244	0.696	0.000296572	Epithelial_cells	SLC7A5
1.6967911	4.715118	0.865	0.522	0.000301825	Epithelial_cells	BCAS1
1.7484237	-3.68598	0.324	0.783	0.00031101	Epithelial_cells	VMO1
1.7999827	-1.38136	0.289	0.783	0.000320181	Epithelial_cells	RDX
1.8068556	2.131888	0.936	1	0.000321403	Epithelial_cells	RRBP1
1.8495772	3.470793	0.867	0.739	0.000329003	Epithelial_cells	PDLIM1
1.8764653	-4.29636	0.106	0.478	0.000333786	Epithelial_cells	GAPLINC
1.9209321	-1.65338	0.037	0.304	0.000341695	Epithelial_cells	CD38
2.0168825	-1.35795	0.905	1	0.000358763	Epithelial_cells	ATP6V0B
2.0406456	3.366438	0.897	0.957	0.00036299	Epithelial_cells	OCIAD2
2.0747674	2.008149	0.931	0.957	0.00036906	Epithelial_cells	KDELR2
2.0949235	-3.8559	0.143	0.565	0.000372645	Epithelial_cells	BCL11A
2.1075221	1.298949	0.987	1	0.000374886	Epithelial_cells	PTMA
2.1377424	-2.34311	0.623	0.87	0.000380262	Epithelial_cells	PEA15
2.4809040	-2.08704	0.398	0.87	0.000441303	Epithelial_cells	TBXAS1
2.5131935	-3.04897	0.257	0.696	0.000447047	Epithelial_cells	SSH1
2.5508205	-4.92858	0.09	0.435	0.00045374	Epithelial_cells	SYTL3
2.6038142	-1.81442	0.724	0.957	0.000463166	Epithelial_cells	RNH1
2.6098813	1.251263	0.992	1	0.000464246	Epithelial_cells	RPS27
2.6263844	4.048306	0.814	0.435	0.000467181	Epithelial_cells	EHF
2.8251327	-2.20184	0.103	0.478	0.000502535	Epithelial_cells	MRAS
2.8383615	2.476636	0.955	1	0.000504888	Epithelial_cells	CYSTM1
2.9562484	4.126704	0.944	0.913	0.000525857	Epithelial_cells	CLDN3
3.2131905	-5.85364	0.074	0.391	0.000571562	Epithelial_cells	ITGAL
3.3311740	-2.77374	0.183	0.609	0.000592549	Epithelial_cells	FERMT3
3.5178717	-3.29696	0.284	0.739	0.000625759	Epithelial_cells	ANPEP
3.6251630	-4.85995	0.09	0.435	0.000644844	Epithelial_cells	GAL3ST4
3.8006402	-2.79335	0.218	0.652	0.000676058	Epithelial_cells	CPNE2
3.8520247	3.502353	0.859	0.652	0.000685198	Epithelial_cells	FAM3B
3.9193837	3.413843	0.775	0.174	0.00069718	Epithelial_cells	CAPN5
3.9700031	-2.02983	0.655	0.957	0.000706184	Epithelial_cells	SMS
3.9849081	-3.95119	0.053	0.348	0.000708835	Epithelial_cells	BCL2
4.0537579	-1.70631	0.793	0.957	0.000721082	Epithelial_cells	ATP6AP2
4.0584630	3.843598	0.692	0.043	0.000721919	Epithelial_cells	PRR15
4.4232332	2.400947	0.958	1	0.000786805	Epithelial_cells	CD24
4.8862714	-2.89072	0.218	0.652	0.00086917	Epithelial_cells	PARVB
4.9304164	-2.98862	0.467	0.826	0.000877022	Epithelial_cells	ZFYVE16

5.3677682	-2.37217	0.284	0.739	0.000954819	Epithelial_cells	GAS6
5.3712847	-1.76861	0.817	1	0.000955444	Epithelial_cells	ARPC4
5.3869646	-3.91953	0.056	0.348	0.000958233	Epithelial_cells	ZFY
5.6415083	-1.82344	0.353	0.783	0.001003512	Epithelial_cells	FLNA
5.7240286	4.151141	0.796	0.304	0.00101819	Epithelial_cells	CGN
5.7474266	-2.12381	0.552	0.87	0.001022352	Epithelial_cells	SH3BGRL
6.1380690	-2.9116	0.255	0.696	0.00109184	Epithelial_cells	IGFLR1
6.3977708	-2.51337	0.536	0.87	0.001138035	Epithelial_cells	ELL2
6.5157443	3.204702	0.769	0.304	0.001159021	Epithelial_cells	AP1M2
6.5422870	-1.48612	0.748	1	0.001163742	Epithelial_cells	CTSH
6.5968511	-2.34431	0.106	0.478	0.001173448	Epithelial_cells	RASA3
6.7118467	-2.03672	0.496	0.87	0.001193903	Epithelial_cells	MAN2B1
7.0806709	-3.3505	0.34	0.739	0.00125951	Epithelial_cells	LHFPL2
7.0820883	-3.34411	0.708	0.87	0.001259762	Epithelial_cells	PLD3
7.3052615	-2.91566	0.17	0.565	0.00129946	Epithelial_cells	IL6
7.8425883	1.47518	0.995	1	0.00139504	Epithelial_cells	RPL7
7.9967166	2.882051	0.958	0.957	0.001422456	Epithelial_cells	CLDN4
8.2983816	4.182887	0.785	0.348	0.001476116	Epithelial_cells	MYH14
8.3901031	2.508098	0.928	1	0.001492432	Epithelial_cells	SPTBN1
8.6259523	-3.2046	0.141	0.522	0.001534384	Epithelial_cells	SYNE1
8.7762624	3.485882	0.841	0.522	0.001561122	Epithelial_cells	OPTN
9.5466628	-1.67074	0.78	1	0.00169816	Epithelial_cells	RNASET2
9.5653673	3.054903	0.825	0.478	0.001701488	Epithelial_cells	ICA1
9.9947199	-1.63164	0.846	1	0.001777861	Epithelial_cells	ATP6AP1
1.0170865	-3.28088	0.268	0.696	0.001809193	Epithelial_cells	SETDB2
1.0685759	4.13293	0.825	0.565	0.001900783	Epithelial_cells	LMO7
1.1048058	-2.93365	0.446	0.826	0.001965229	Epithelial_cells	PLEKHM2
1.1110557	-1.10633	0.987	1	0.001976346	Epithelial_cells	ACTB
1.1743259	3.864895	0.788	0.391	0.002088891	Epithelial_cells	TMPRSS4
1.1971067	-1.87994	0.806	1	0.002129414	Epithelial_cells	NFKBIZ
1.1971955	-1.79201	0.443	0.913	0.002129571	Epithelial_cells	CXCL1
1.1985894	-2.25093	0.233	0.652	0.002132051	Epithelial_cells	SEPT6
1.2492885	-2.64589	0.475	0.783	0.002222234	Epithelial_cells	ADRBK2
1.2737673	-2.00656	0.655	0.913	0.002265777	Epithelial_cells	USF2
1.2913540	3.168587	0.708	0.13	0.002297061	Epithelial_cells	PTK2
1.3253512	-1.38032	0.889	1	0.002357535	Epithelial_cells	S100A4
1.3409682	-1.77198	0.491	0.87	0.002385314	Epithelial_cells	TLN1
1.3424598	-3.76276	0.252	0.652	0.002387968	Epithelial_cells	PLEKHO2
1.3612891	-2.92289	0.459	0.826	0.002421461	Epithelial_cells	GLA
1.3859868	-1.77937	0.581	0.913	0.002465393	Epithelial_cells	PTMS
1.4081007	3.617944	0.928	0.826	0.00250473	Epithelial_cells	LSM8
1.4272966	-1.75341	0.846	1	0.002538875	Epithelial_cells	CHMP1B
1.4497302	3.11127	0.822	0.391	0.00257878	Epithelial_cells	ABLIM1
1.4561570	-2.88139	0.332	0.739	0.002590212	Epithelial_cells	USP36
1.4787349	4.676044	0.87	0.609	0.002630374	Epithelial_cells	MET
1.6068098	-2.86325	0.549	0.826	0.002858193	Epithelial_cells	NFKB1
1.6140262	-2.45026	0.52	0.826	0.00287103	Epithelial_cells	PGD
1.6247488	-1.8279	0.735	0.913	0.002890103	Epithelial_cells	TCIRG1
1.7115950	2.948443	0.873	0.565	0.003044585	Epithelial_cells	TPD52
1.7830072	3.130227	0.841	0.609	0.003171613	Epithelial_cells	CREB3L1
1.9284815	-2.94327	0.65	0.87	0.003430383	Epithelial_cells	SLC43A2
1.9485609	-3.53786	0.297	0.696	0.0034661	Epithelial_cells	STAT5A
1.9518205	-2.58294	0.666	0.957	0.003471898	Epithelial_cells	B4GALT1
1.9948370	-1.7993	0.817	0.957	0.003548416	Epithelial_cells	CD44
2.0048568	3.387009	0.836	0.652	0.003566239	Epithelial_cells	PTPRF
2.0831246	3.80236	0.703	0.13	0.003705462	Epithelial_cells	LAMC2
2.1497456	2.241584	0.912	0.87	0.003823968	Epithelial_cells	TOB1
2.2565814	-1.12633	0.918	1	0.004014007	Epithelial_cells	PPP1R15A

2.3769661	2.912404	0.796	0.391	0.004228147	Epithelial_cells	TJP1
2.4212293	3.697717	0.698	0.174	0.004306883	Epithelial_cells	ENAH
2.4389709	-1.65743	0.668	0.913	0.004338442	Epithelial_cells	DPP7
2.5353071	2.94157	0.854	0.739	0.004509804	Epithelial_cells	CDH1
2.6181230	2.240427	0.857	0.739	0.004657117	Epithelial_cells	MARCKSL1
2.6697027	-3.16291	0.393	0.739	0.004748867	Epithelial_cells	CPM
2.6741270	-3.39504	0.337	0.739	0.004756737	Epithelial_cells	MOB3A
2.7557401	2.948624	0.836	0.391	0.004901911	Epithelial_cells	BAIAP2L1
2.8394657	-1.033	0.926	1	0.005050842	Epithelial_cells	GABARAP
2.8601062	-1.6797	0.499	0.87	0.005087557	Epithelial_cells	RGCC
2.8884288	-3.28506	0.268	0.652	0.005137937	Epithelial_cells	SNAI1
2.8999568	-2.3195	0.146	0.522	0.005158443	Epithelial_cells	PDGFB
2.9028124	4.234966	0.801	0.435	0.005163523	Epithelial_cells	CYP3A5
2.9826579	-4.1753	0.178	0.565	0.005305552	Epithelial_cells	RELT
3.0113383	3.279436	0.759	0.304	0.005356569	Epithelial_cells	NR2F6
3.0994380	-2.58174	0.22	0.652	0.00551328	Epithelial_cells	IFI16
3.1405906	5.038517	0.775	0.304	0.005586483	Epithelial_cells	PLAC8
3.1410942	2.771809	0.891	0.739	0.005587378	Epithelial_cells	SPATS2L
3.1674496	2.529183	0.841	0.565	0.005634259	Epithelial_cells	TUBB4B
3.1720989	2.517598	0.814	0.348	0.00564253	Epithelial_cells	ASL
3.1990232	3.113422	0.812	0.522	0.005690422	Epithelial_cells	DDR1
3.2002921	1.378784	0.934	1	0.00569268	Epithelial_cells	EDF1
3.2031867	3.410799	0.79	0.478	0.005697829	Epithelial_cells	MPST
3.2354270	3.524738	0.772	0.348	0.005755178	Epithelial_cells	RASEF
3.2616495	-3.38966	0.228	0.609	0.005801822	Epithelial_cells	INPP5D
3.3042922	-2.31106	0.557	0.87	0.005877675	Epithelial_cells	ARHGAP18
3.3471276	4.211085	0.92	0.87	0.005953871	Epithelial_cells	TSPAN1
3.3789772	2.640236	0.493	0.913	0.006010525	Epithelial_cells	JCHAIN
3.4210369	-2.03274	0.284	0.696	0.006085341	Epithelial_cells	AKNA
3.5350387	3.120968	0.727	0.217	0.006288127	Epithelial_cells	B3GNT3
3.5908462	2.606212	0.854	0.696	0.006387397	Epithelial_cells	LIMA1
3.5997466	-4.9257	0.066	0.348	0.006403229	Epithelial_cells	NFAM1
3.6390593	-2.37737	0.35	0.739	0.006473159	Epithelial_cells	KIAA0930
3.6744762	-1.63875	0.782	0.957	0.006536158	Epithelial_cells	YWHAH
3.6914423	-1.89663	0.729	1	0.006566338	Epithelial_cells	BLOC1S6
3.8772948	3.157499	0.82	0.609	0.006896932	Epithelial_cells	KRT10
3.9265360	3.979974	0.653	0.087	0.006984522	Epithelial_cells	ARFGEF3
3.9288189	3.048913	0.769	0.304	0.006988583	Epithelial_cells	EPS8L3
3.9780510	-2.40447	0.408	0.783	0.007076157	Epithelial_cells	GGA1
4.0379695	3.933701	0.698	0.174	0.00718274	Epithelial_cells	GSDMB
4.1224837	-3.03178	0.149	0.522	0.007333074	Epithelial_cells	ITPR1
4.2340959	3.638228	0.761	0.348	0.00753161	Epithelial_cells	CXADR
4.4765968	1.603123	0.979	1	0.00796297	Epithelial_cells	GSTP1
4.5050456	-3.13001	0.401	0.739	0.008013575	Epithelial_cells	NAGA
5.0489157	-1.88604	0.679	0.913	0.008981011	Epithelial_cells	CYTH1
5.0737946	2.749895	0.687	0.13	0.009025266	Epithelial_cells	LRP5
5.1782454	4.836266	0.775	0.261	0.009211063	Epithelial_cells	COL16A1
5.3426637	-2.2718	0.533	0.87	0.00950353	Epithelial_cells	FNIP2
5.5805863	-3.3965	0.034	0.261	0.009926747	Epithelial_cells	ZMIZ1-AS1
5.7969493	-3.47751	0.119	0.478	0.010311614	Epithelial_cells	ARHGEF40
5.8495620	3.269548	0.719	0.217	0.010405201	Epithelial_cells	MFSD6
5.9374760	-2.96578	0.634	0.826	0.010561582	Epithelial_cells	WTAP
6.0540401	-3.08017	0.064	0.348	0.010768927	Epithelial_cells	CALHM2
6.1590317	-1.20068	0.992	1	0.010955686	Epithelial_cells	TMSB4X
6.2288824	3.998386	0.66	0.13	0.011079936	Epithelial_cells	POF1B
6.2430050	3.43549	0.859	0.783	0.011105057	Epithelial_cells	PLA2G10
6.3394121	-1.22314	0.836	1	0.011276546	Epithelial_cells	BHLHE40
6.3507521	-1.22032	0.769	0.957	0.011296718	Epithelial_cells	TALDO1

6.4000021	4.823329	0.753	0.348	0.011384324	Epithelial_cells	AGR3
6.5420935	2.849542	0.737	0.217	0.011637076	Epithelial_cells	TMEM54
6.6543366	2.923061	0.769	0.304	0.011836734	Epithelial_cells	FAM221A
6.7940717	2.385759	0.875	0.826	0.012085295	Epithelial_cells	TSTA3
6.9812028	-1.48491	0.377	0.826	0.012418164	Epithelial_cells	RPS4Y1
7.1065345	3.860133	0.74	0.304	0.012641104	Epithelial_cells	ITGA2
7.1188241	-4.06718	0.088	0.391	0.012662964	Epithelial_cells	TMC8
7.1207496	2.144476	0.865	0.739	0.012666389	Epithelial_cells	ARPC1A
7.3044726	-3.20776	0.353	0.739	0.012993196	Epithelial_cells	IL1RN
7.5476878	-3.6562	0.21	0.565	0.013425827	Epithelial_cells	CCDC69
7.6670940	-3.21248	0.451	0.739	0.013638227	Epithelial_cells	ALCAM
7.9908280	-3.18543	0.448	0.783	0.014214085	Epithelial_cells	CLN8
8.0874689	-3.88918	0.066	0.348	0.01438599	Epithelial_cells	MB21D2
8.0922951	2.737571	0.862	0.87	0.014394575	Epithelial_cells	ITGA6
8.5211448	1.293721	0.973	1	0.015157412	Epithelial_cells	RPL30
8.6413472	3.77841	0.772	0.478	0.015371228	Epithelial_cells	SOX9
8.6739046	3.001736	0.777	0.348	0.015429142	Epithelial_cells	ASAP2
8.7809005	-1.50459	0.586	0.913	0.015619466	Epithelial_cells	RB1
8.8849179	-1.99915	0.918	1	0.015804492	Epithelial_cells	TIMP1
9.0706145	3.627985	0.581	0	0.016134809	Epithelial_cells	DLG3
9.1593448	2.515417	0.814	0.478	0.016292643	Epithelial_cells	NOSIP
9.3036153	3.114503	0.806	0.522	0.016549271	Epithelial_cells	ABHD17C
9.4243543	-1.83526	0.788	0.913	0.016764041	Epithelial_cells	ATP6V0D1
9.4713813	3.440435	0.724	0.304	0.016847693	Epithelial_cells	RHPN2
9.5692680	2.271186	0.881	0.87	0.017021814	Epithelial_cells	SF3B6
9.8957595	-1.10229	0.849	0.913	0.017602577	Epithelial_cells	ARPC5
9.9880773	-2.22748	0.523	0.87	0.017766792	Epithelial_cells	STAT2
1.0145615	-2.92446	0.273	0.652	0.018047021	Epithelial_cells	FES
1.0242385	3.439032	0.788	0.435	0.018219155	Epithelial_cells	FUT3
1.0490827	-3.46676	0.037	0.261	0.018661084	Epithelial_cells	SEMA6B
1.0513853	2.953658	0.92	0.826	0.018702043	Epithelial_cells	TES
1.0518560	2.542494	0.82	0.565	0.018710415	Epithelial_cells	GALNT3
1.0726062	3.184839	0.801	0.609	0.019079521	Epithelial_cells	PPIC
1.0733874	-4.53389	0.13	0.478	0.019093417	Epithelial_cells	WDR7
1.0758195	-2.28295	0.435	0.783	0.019136678	Epithelial_cells	TET2
1.0897762	-3.41984	0.13	0.478	0.01938494	Epithelial_cells	SH3TC1
1.1205523	-1.03586	0.902	1	0.019932385	Epithelial_cells	CSTB
1.1593992	1.850905	0.897	0.913	0.020623393	Epithelial_cells	MRPS21
1.2011547	4.165397	0.854	0.739	0.021366141	Epithelial_cells	ID1
1.2380144	2.478483	0.79	0.478	0.022021801	Epithelial_cells	NDFIP2
1.2723964	-3.83069	0.053	0.304	0.022633387	Epithelial_cells	DYSF
1.3000517	-4.02496	0.053	0.304	0.023125321	Epithelial_cells	HIC1
1.3518026	1.430349	0.981	1	0.024045866	Epithelial_cells	CHCHD2
1.3579048	3.427805	0.623	0.087	0.024154412	Epithelial_cells	NOSTRIN
1.3614259	-1.59926	0.939	1	0.024217044	Epithelial_cells	DUSP1
1.3799126	-2.58428	0.255	0.652	0.024545886	Epithelial_cells	PRDM1
1.4022689	3.628827	0.859	0.565	0.02494356	Epithelial_cells	ASS1
1.4156838	1.26499	0.963	1	0.025182184	Epithelial_cells	NDUFA13
1.4447863	2.734477	0.703	0.261	0.02569986	Epithelial_cells	ZFAND1
1.4467842	-2.13974	0.557	0.913	0.025735398	Epithelial_cells	MAP2K1
1.4524686	3.008995	0.684	0.174	0.025836512	Epithelial_cells	IRF6
1.4594468	2.695002	0.817	0.478	0.025960641	Epithelial_cells	C1orf106
1.4634442	2.872455	0.729	0.304	0.026031747	Epithelial_cells	CHMP4C
1.5628292	-2.82083	0.069	0.348	0.027799607	Epithelial_cells	GNG2
1.5631189	-4.25052	0.141	0.478	0.027804759	Epithelial_cells	BMP2K
1.6314237	2.20755	0.897	0.957	0.029019765	Epithelial_cells	TMBIM1
1.6457209	2.873822	0.772	0.348	0.029274083	Epithelial_cells	MUC3A
1.6768931	3.489454	0.674	0.174	0.029828575	Epithelial_cells	SH2D4A

1.6836477	-1.3925	0.891	1	0.029948726	Epithelial_cells	MCL1
1.6884096	1.393913	0.958	1	0.030033431	Epithelial_cells	KRTCAP2
1.7253642	-3.05062	0.366	0.696	0.030690779	Epithelial_cells	NABP1
1.7343258	2.801118	0.743	0.304	0.030850187	Epithelial_cells	HDHD3
1.7604617	3.273557	0.735	0.304	0.031315094	Epithelial_cells	AKR1C3
1.7811951	-1.64939	0.653	0.87	0.031683899	Epithelial_cells	BID
1.7871804	3.275458	0.674	0.217	0.031790365	Epithelial_cells	HIP1R
1.7906615	2.130146	0.865	0.739	0.031852288	Epithelial_cells	PSMA4
1.8360793	2.861513	0.737	0.261	0.032660179	Epithelial_cells	SLC2A1
1.8382145	-3.06942	0.114	0.435	0.03269816	Epithelial_cells	ATG16L2
1.8878661	2.739243	0.769	0.304	0.033581363	Epithelial_cells	TNFSF10
1.8920976	-2.93236	0.244	0.609	0.033656633	Epithelial_cells	RGS19
1.9200243	1.64602	0.918	0.957	0.034153393	Epithelial_cells	NDUFS6
1.9642689	-1.97621	0.565	0.826	0.034940415	Epithelial_cells	FOXN3
2.0082762	3.57655	0.708	0.261	0.035723218	Epithelial_cells	MLLT4
2.0085824	-3.69182	0.257	0.609	0.035728664	Epithelial_cells	SGPP1
2.0179076	3.823624	0.817	0.522	0.035894542	Epithelial_cells	CEACAM1
2.0290817	-1.69578	0.769	0.957	0.036093305	Epithelial_cells	FABP5
2.0564147	-2.21605	0.499	0.783	0.036579505	Epithelial_cells	WARS
2.0722320	3.283042	0.727	0.304	0.036860864	Epithelial_cells	TST
2.0921028	2.095242	0.897	0.739	0.037214326	Epithelial_cells	LRP10
2.1309067	-2.3339	0.09	0.391	0.037904569	Epithelial_cells	ARMCX1
2.1883710	3.053237	0.629	0.13	0.038926744	Epithelial_cells	YAP1
2.2905037	-1.08759	0.905	1	0.04074348	Epithelial_cells	SDCBP
2.3072607	2.828353	0.862	0.913	0.041041554	Epithelial_cells	GMDS
2.3351157	-1.53779	0.684	0.87	0.04153704	Epithelial_cells	WNK1
2.3819587	4.518968	0.764	0.435	0.042370282	Epithelial_cells	GCNT3
2.4386541	2.726339	0.822	0.565	0.04337878	Epithelial_cells	PARD6B
2.4951836	3.258021	0.597	0.043	0.044384326	Epithelial_cells	EPN2
2.5476156	2.512452	0.743	0.217	0.045316987	Epithelial_cells	ERBB2
2.5922405	2.692461	0.859	0.783	0.046110775	Epithelial_cells	RAB11FIP1
2.5939186	-2.20935	0.472	0.783	0.046140625	Epithelial_cells	GALM
2.6006724	2.789541	0.782	0.435	0.046260762	Epithelial_cells	LAPTM4B
2.7640708	3.067838	0.801	0.652	0.049167292	Epithelial_cells	PRR15L
2.7790206	-2.51372	0.369	0.739	0.04943322	Epithelial_cells	SWAP70
4.8421193	7.746557	0.826	0.016	8.61316190704553e-52	Monocyte	SPI1
2.8381433	6.477973	0.87	0.029	5.04848936848425e-46	Monocyte	STAB1
4.2020350	7.439053	0.957	0.058	7.47458001553445e-41	Monocyte	CD4
1.4453063	6.163412	0.739	0.021	2.57091086326806e-40	Monocyte	WAS
4.5308119	7.779425	0.826	0.034	8.05940837546452e-40	Monocyte	HCK
6.3430011	6.999631	0.652	0.013	1.12829303965285e-38	Monocyte	ARHGAP9
1.3118593	7.563588	0.957	0.064	2.33353549141394e-38	Monocyte	IGSF6
1.0038903	7.951298	0.609	0.011	1.78572013715704e-37	Monocyte	SIGLEC9
1.9469720	8.596314	0.696	0.021	3.46327380243695e-37	Monocyte	SCIMP
2.9865074	7.436055	0.696	0.021	5.31239951648416e-37	Monocyte	LY86
3.5531981	7.07441	0.652	0.016	6.32042895168389e-37	Monocyte	LOC653786
4.6013163	7.683176	0.957	0.072	8.18482159767548e-37	Monocyte	LILRB4
8.4546918	5.516263	0.826	0.045	1.50392058979775e-35	Monocyte	PARVG
1.6611891	7.711117	0.652	0.019	2.95492333551637e-35	Monocyte	NCKAP1L
5.0863000	5.242397	0.957	0.077	9.04751057536636e-35	Monocyte	FYB
8.3199093	8.159318	1	0.095	1.47994546813852e-34	Monocyte	CD86
1.0524962	7.190712	0.87	0.058	1.87218041439784e-34	Monocyte	LAT2
1.4826176	7.542443	0.783	0.04	2.63728032924486e-34	Monocyte	LILRB2
1.0702184	7.270674	0.652	0.021	1.90370457564885e-33	Monocyte	PIK3R5
3.3323775	7.548781	0.826	0.053	5.92763322447451e-33	Monocyte	SLAMF8
7.3937508	7.82422	0.957	0.09	1.31520040358467e-32	Monocyte	PTPRC
2.0949809	7.336106	0.87	0.066	3.72655206531053e-32	Monocyte	CD300A
3.1160058	6.760201	0.783	0.045	5.54275115076699e-32	Monocyte	BCAT1

7.6880932	6.263053	0.652	0.024	1.367558032116e-31	Monocyte	CD72
2.7760090	4.301504	0.826	0.058	4.93796490076588e-31	Monocyte	NCF2
2.8318873	6.676356	0.87	0.069	5.03736124904781e-31	Monocyte	CELF2
3.8237709	6.838729	0.913	0.082	6.80172369225547e-31	Monocyte	MPEG1
6.6740358	4.065226	0.739	0.04	1.18717749141166e-30	Monocyte	DOCK10
1.6948109	6.031053	0.913	0.085	3.01472976545431e-30	Monocyte	FGL2
1.7549450	7.351163	0.87	0.074	3.12169628899076e-30	Monocyte	SLC37A2
2.6250130	7.314916	0.609	0.021	4.6693732722462e-30	Monocyte	RNASE2
3.5038547	6.910988	0.609	0.021	6.2326568528348e-30	Monocyte	GPR132
3.9329975	7.565517	0.783	0.053	6.99601599369591e-30	Monocyte	SLA
8.4783343	7.876047	0.913	0.09	1.5081261176609e-29	Monocyte	PLEK
1.5893496	8.290938	0.826	0.066	2.82713511518224e-29	Monocyte	PILRA
1.6337933	7.197326	0.652	0.029	2.90619158912147e-29	Monocyte	CCR1
2.1640139	8.366387	0.783	0.056	3.84934795256337e-29	Monocyte	FPR3
2.3690212	6.606252	0.739	0.045	4.21401495526886e-29	Monocyte	DOCK2
5.7706424	5.588324	0.652	0.029	1.02648187971263e-28	Monocyte	CD37
2.6474015	6.858946	0.696	0.04	4.70919787414709e-28	Monocyte	NCF4
3.6032086	5.989372	0.783	0.058	6.40938754474914e-28	Monocyte	EVI2B
1.3277545	7.802677	0.609	0.027	2.36180981733499e-27	Monocyte	SIGLEC1
1.4289429	7.391899	0.913	0.103	2.54180364933891e-27	Monocyte	LST1
2.2586175	7.214819	0.696	0.042	4.0176288889543e-27	Monocyte	MNDA
2.3657373	6.871962	0.696	0.042	4.20817359681507e-27	Monocyte	ADGRE2
4.0250793	7.643777	0.391	0.003	7.15981121433951e-27	Monocyte	LUCAT1
7.1754304	4.643553	0.739	0.05	1.27636556235776e-26	Monocyte	LRRC25
7.6711083	6.972068	0.739	0.053	1.3645367460705e-26	Monocyte	SLC8A1
8.2186381	6.756621	0.565	0.021	1.46193135280931e-26	Monocyte	CST7
8.5350982	5.175121	0.783	0.061	1.51822327308297e-26	Monocyte	EPB41L3
2.9678121	7.52236	0.652	0.037	5.27914433686696e-26	Monocyte	CD209
3.3451190	6.748075	0.739	0.056	5.95029782304985e-26	Monocyte	SIGLEC12
4.6700759	8.702953	0.913	0.114	8.30713117270542e-26	Monocyte	CD163
1.1728994	5.646174	0.739	0.056	2.08635350655818e-25	Monocyte	ZNF385A
1.9290301	5.182091	0.957	0.13	3.43135885640109e-25	Monocyte	ZEB2
2.0137181	6.275166	0.826	0.082	3.5820019025486e-25	Monocyte	IL10RA
2.1215909	4.583408	0.739	0.058	3.77388595322836e-25	Monocyte	C3AR1
2.2479867	5.781711	0.826	0.082	3.99871887975816e-25	Monocyte	MRC1
4.6670392	5.803963	1	0.156	8.30172936946182e-25	Monocyte	SLC2A3
7.0947469	8.211684	0.913	0.122	1.26201358888188e-24	Monocyte	MS4A4A
8.5304243	3.919545	0.522	0.019	1.51739187744021e-24	Monocyte	TFEC
9.7053041	6.089388	0.609	0.032	1.72637949429507e-24	Monocyte	GPR84
1.7525268	8.411875	0.826	0.09	3.1173947552672e-24	Monocyte	PLA2G7
2.7344993	7.193169	0.391	0.005	4.86412742787866e-24	Monocyte	MS4A14
1.4385090	6.229593	0.609	0.034	2.55881996666241e-23	Monocyte	HVCN1
2.0513408	6.747981	0.348	0.003	3.64892510173093e-23	Monocyte	TLR8
2.1261254	7.173627	0.435	0.011	3.78195194005354e-23	Monocyte	CD300LF
2.2436625	5.172527	0.522	0.021	3.9910269645106e-23	Monocyte	WDFY4
3.1592723	6.28735	0.957	0.156	5.61971363316526e-23	Monocyte	CSF1R
8.5646793	4.634827	0.783	0.082	1.52348516730504e-22	Monocyte	C1orf162
9.3682386	4.983354	0.696	0.056	1.66642228896005e-22	Monocyte	DOK3
1.0226725	4.519105	0.478	0.016	1.81912994244639e-22	Monocyte	LILRB1
1.0702923	5.683331	0.609	0.037	1.90383595281311e-22	Monocyte	ABI3
1.0934912	6.526149	0.913	0.138	1.94510220164088e-22	Monocyte	LAIR1
1.1899491	6.575951	0.826	0.098	2.11668158501215e-22	Monocyte	SAMSN1
1.9618526	7.136238	1	0.196	3.48974355059852e-22	Monocyte	MS4A7
2.0212563	5.26043	0.826	0.098	3.59541083497962e-22	Monocyte	MYO5A
2.1689598	7.828266	0.913	0.143	3.85814576444336e-22	Monocyte	SLC11A1
2.3119275	6.674121	0.783	0.085	4.1124567981916e-22	Monocyte	IFITM10
2.5536870	5.450004	0.913	0.135	4.54249843992616e-22	Monocyte	STX11
3.7117176	6.21477	0.522	0.024	6.60240334794029e-22	Monocyte	CYTH4

4.9240559	4.29342	0.826	0.093	8.75891067753544e-22	Monocyte	LSP1
7.6788150	6.806624	0.913	0.146	1.36590761720575e-21	Monocyte	CYBB
1.0058167	4.676312	0.652	0.05	1.78914690492731e-21	Monocyte	NLRP3
1.0845029	4.346335	0.522	0.024	1.9291137979089e-21	Monocyte	IKZF1
1.3489157	6.341801	0.478	0.019	2.39945128128402e-21	Monocyte	NKG7
1.6414291	4.24666	0.826	0.106	2.91977409909175e-21	Monocyte	MPP1
1.7489224	6.226735	0.826	0.106	3.11098333677806e-21	Monocyte	HCLS1
2.1514917	6.738776	0.435	0.013	3.82707344050908e-21	Monocyte	CD80
9.6959308	4.04548	0.87	0.13	1.72471218219136e-20	Monocyte	CLEC7A
9.8582970	4.693556	0.957	0.186	1.75359388284511e-20	Monocyte	FCGR2A
1.1004992	5.626681	0.783	0.09	1.95756798631266e-20	Monocyte	FCGR1A
1.3419511	7.689629	0.609	0.045	2.38706274526129e-20	Monocyte	OTOA
1.3986728	7.469519	0.609	0.045	2.48795925647676e-20	Monocyte	LINC01272
1.4671917	6.799015	0.783	0.095	2.60984068746943e-20	Monocyte	GRASP
1.9822103	4.587802	0.913	0.149	3.52595585946885e-20	Monocyte	GMFG
2.3411041	2.304403	0.87	0.135	4.16435614341745e-20	Monocyte	LCP1
2.9420248	6.492155	0.478	0.021	5.23327388017832e-20	Monocyte	TNFSF8
3.3691027	7.807408	0.696	0.069	5.99295990388156e-20	Monocyte	ADAMDEC1
3.5754153	4.708186	0.783	0.09	6.35994877840882e-20	Monocyte	OLFML2B
3.8642559	7.961482	0.957	0.199	6.87373853828961e-20	Monocyte	MSR1
4.2357913	4.026452	0.522	0.027	7.53462570407935e-20	Monocyte	TGFB1
4.8500634	8.242635	0.957	0.202	8.62729284831922e-20	Monocyte	C5AR1
4.9001263	7.278455	0.522	0.029	8.71634474681443e-20	Monocyte	AQP9
5.9128240	7.460756	0.522	0.029	1.05177314665619e-19	Monocyte	IL7R
6.2915904	6.441799	0.391	0.011	1.11914811098609e-19	Monocyte	CD33
9.8536208	6.687012	0.304	0.003	1.75276207244572e-19	Monocyte	HK3
1.3438357	5.866937	0.304	0.003	2.39041507879569e-19	Monocyte	MYO1G
1.4287277	6.025895	0.696	0.072	2.54142088810027e-19	Monocyte	LCP2
1.6392011	4.547081	0.87	0.141	2.91581092838121e-19	Monocyte	HAVCR2
1.6897859	3.836208	0.913	0.164	3.00579121602173e-19	Monocyte	MAFB
2.4293054	4.318616	0.957	0.188	4.32124861205188e-19	Monocyte	BASP1
2.6771741	7.989427	0.652	0.061	4.76215746482583e-19	Monocyte	OSM
3.0319304	7.27522	0.478	0.024	5.3931979462151e-19	Monocyte	CD48
3.6905214	4.258801	0.565	0.04	6.5646995828502e-19	Monocyte	OLR1
4.5472750	6.929024	0.522	0.032	8.08869294507337e-19	Monocyte	CSF3R
6.1954015	7.252479	0.913	0.18	1.10203803197545e-18	Monocyte	BCL2A1
8.5196307	3.256149	0.87	0.138	1.515471920774e-18	Monocyte	PECAM1
1.1082811	7.416831	0.957	0.22	1.97141049372739e-18	Monocyte	ITGB2
1.1949656	5.582129	0.957	0.212	2.12560483434313e-18	Monocyte	ARL4C
1.2157824	4.430121	0.696	0.069	2.16263391083979e-18	Monocyte	NRP2
1.3588965	6.28799	0.739	0.09	2.41720510366268e-18	Monocyte	SLC16A10
1.5839841	7.652091	0.609	0.053	2.81759102080353e-18	Monocyte	FPR1
1.5919069	5.114388	0.739	0.09	2.83168407581481e-18	Monocyte	RNASE6
1.9799299	7.991144	0.435	0.019	3.52189933810823e-18	Monocyte	DOK2
2.0413033	7.257091	0.565	0.042	3.63107040934338e-18	Monocyte	PLCB2
2.0707033	6.139023	0.348	0.008	3.68336720005865e-18	Monocyte	SLC1A3
2.2779394	4.60973	0.826	0.125	4.05199870197184e-18	Monocyte	TLR2
2.3493300	5.191167	0.87	0.156	4.17898829942363e-18	Monocyte	CD53
2.3557170	6.290827	0.348	0.008	4.19034941715198e-18	Monocyte	OSCAR
2.3837974	4.323464	0.913	0.17	4.24029896091768e-18	Monocyte	PDE4B
2.8213471	4.304928	0.913	0.175	5.01861222216266e-18	Monocyte	PLXNC1
3.4607891	7.414027	0.391	0.013	6.15605181716966e-18	Monocyte	PRAM1
4.5910831	5.011162	0.826	0.127	8.16661865686564e-18	Monocyte	RUNX3
5.8662261	5.876957	1	0.276	1.04348430666506e-17	Monocyte	AIF1
6.7553184	5.591501	0.522	0.034	1.20163605447151e-17	Monocyte	CSF2RB
6.9109723	4.55311	0.783	0.103	1.22932375487497e-17	Monocyte	SLAMF7
1.1800592	5.101377	0.522	0.034	2.09908937308123e-17	Monocyte	LY9
1.6671360	8.609754	0.87	0.172	2.96550165788319e-17	Monocyte	FCGR3A

2.0623667	4.93311	0.826	0.143	3.66853796362628e-17	Monocyte	CD84
2.5424189	5.45932	0.609	0.056	4.52245474267476e-17	Monocyte	KLHL6
2.9344207	7.885422	0.696	0.085	5.21974769234286e-17	Monocyte	MMP19
2.9810615	3.005604	0.826	0.141	5.30271235604125e-17	Monocyte	MS4A6A
3.0145239	6.819615	0.435	0.021	5.36223528020466e-17	Monocyte	KMO
3.2630249	7.089092	0.87	0.175	5.80426882293344e-17	Monocyte	CECR1
3.3527651	6.142377	0.957	0.22	5.96389870697365e-17	Monocyte	PTGS2
3.8408865	5.085206	0.783	0.111	6.8321690636355e-17	Monocyte	ARHGAP30
3.9286248	5.701375	0.957	0.236	6.98823783450023e-17	Monocyte	SIRPA
3.9394103	3.518584	0.826	0.127	7.00742317051714e-17	Monocyte	PLXND1
4.3242087	4.673629	0.739	0.101	7.69190258122981e-17	Monocyte	MYO1F
4.3819551	6.651183	0.478	0.029	7.79462188857596e-17	Monocyte	ASGR1
4.3924901	7.909059	0.304	0.005	7.81336151056623e-17	Monocyte	TMEM52B
4.4632629	3.700006	0.652	0.066	7.93925204817406e-17	Monocyte	RCSD1
5.6474633	7.109873	0.565	0.048	1.00457078570431e-16	Monocyte	TREM1
6.9634442	3.681378	0.435	0.021	1.23865745882302e-16	Monocyte	TDRD6
1.1184524	6.636328	0.391	0.016	1.98950313559904e-16	Monocyte	ARHGAP25
1.4426303	5.911357	0.739	0.106	2.56615083181466e-16	Monocyte	TNFSF13B
1.5677413	8.294624	0.348	0.011	2.78869838842731e-16	Monocyte	RETN
2.1052021	6.170025	0.348	0.011	3.74473352311882e-16	Monocyte	HTRA4
2.4761683	5.949586	0.522	0.04	4.40460830685694e-16	Monocyte	IL4I1
3.0261333	4.286952	0.913	0.204	5.38288606901066e-16	Monocyte	SLC31A2
4.3629117	7.420609	0.435	0.024	7.76074736031504e-16	Monocyte	LILRA5
4.7760539	5.804038	0.261	0.003	8.49564480322138e-16	Monocyte	F13A1
4.7760539	6.519549	0.261	0.003	8.49564480322138e-16	Monocyte	LOC101928489
5.8739210	7.479323	1	0.35	1.04485307902623e-15	Monocyte	ITGAX
6.7265858	8.635266	0.957	0.286	1.19652509558702e-15	Monocyte	SDS
6.8531921	5.494065	0.565	0.05	1.21904581119444e-15	Monocyte	ITGAM
6.9869254	5.482106	0.609	0.064	1.24283429172796e-15	Monocyte	DSE
1.0819482	5.186093	0.609	0.064	1.92456956578238e-15	Monocyte	ICAM4
1.1265439	7.111063	0.739	0.117	2.00389633139653e-15	Monocyte	HCST
1.6547654	5.125324	0.696	0.093	2.94349679204748e-15	Monocyte	FAM49A
1.7494697	6.577455	0.522	0.042	3.11195674398565e-15	Monocyte	TTYH2
1.9096737	7.003189	1	0.369	3.39692770923802e-15	Monocyte	LAPTM5
2.5224967	4.325447	0.913	0.223	4.48701713927971e-15	Monocyte	GPSM3
3.4349664	7.325804	0.957	0.297	6.11011824992164e-15	Monocyte	G0S2
3.6816508	5.226974	0.913	0.218	6.54892060676586e-15	Monocyte	EMILIN2
3.9642559	3.993528	0.609	0.064	7.05161851322375e-15	Monocyte	CLEC11A
4.4290414	6.122131	0.435	0.027	7.87837888169562e-15	Monocyte	DPEP2
4.5054688	5.75592	1	0.377	8.01432807729879e-15	Monocyte	GPNMB
4.9853001	4.486816	0.826	0.151	8.86785182362532e-15	Monocyte	PREX1
5.5412553	6.2891	0.522	0.045	9.85678505671822e-15	Monocyte	MARCH1
5.5613517	5.294863	0.478	0.034	9.89253253148885e-15	Monocyte	TM6SF1
5.6856646	3.797988	0.87	0.164	1.01136602008246e-14	Monocyte	APOBR
6.7208002	6.927865	1	0.393	1.1954959494753e-14	Monocyte	TYROBP
7.1571250	3.469715	1	0.286	1.27310939896964e-14	Monocyte	MSN
9.8756958	3.901261	1	0.329	1.75668877628208e-14	Monocyte	PMP22
1.0457295	4.668975	1	0.286	1.86014380205521e-14	Monocyte	NR4A3
1.0528052	4.956	0.348	0.013	1.8727300471447e-14	Monocyte	LINC01094
1.3775629	4.222289	0.783	0.133	2.4504088877863e-14	Monocyte	DOCK4
1.4162034	5.194535	0.913	0.236	2.51914273793344e-14	Monocyte	SLCO2B1
1.5357527	5.190725	0.348	0.013	2.73179705989805e-14	Monocyte	GIMAP6
1.8317784	7.390325	0.478	0.037	3.25836741829235e-14	Monocyte	CLEC5A
1.9322359	4.518339	0.739	0.117	3.43706137704465e-14	Monocyte	CFD
2.6345998	4.542647	0.87	0.22	4.6864262516338e-14	Monocyte	HLA-DRB6
3.9685862	6.106548	1	0.424	7.05932120806899e-14	Monocyte	FCER1G
4.9258214	4.101987	0.783	0.146	8.76205122467278e-14	Monocyte	ST3GAL5
5.3085495	4.157391	0.522	0.048	9.44284801090477e-14	Monocyte	CLEC4A

5.9290029	3.749796	0.652	0.088	1.05465104517643e-13	Monocyte	PDPN
5.9444203	5.361369	0.957	0.329	1.05739348621189e-13	Monocyte	GPR183
7.1850719	5.088144	0.783	0.149	1.27808059754294e-13	Monocyte	PTAFR
7.4642244	3.56824	0.739	0.125	1.3277362375004e-13	Monocyte	LY96
9.1280228	4.837322	1	0.424	1.62369269601718e-13	Monocyte	TNFRSF1B
1.1025271	6.765979	0.913	0.276	1.96117527416398e-13	Monocyte	CD14
1.1619665	5.720233	0.913	0.281	2.06690606147028e-13	Monocyte	HLA-DMB
1.2080186	8.427314	0.609	0.08	2.14882353316833e-13	Monocyte	VSIG4
1.2235477	3.585885	1	0.326	2.17644672171582e-13	Monocyte	KCTD12
1.2392315	4.762768	0.609	0.074	2.20434501520158e-13	Monocyte	HSD17B14
1.4620036	6.578163	0.261	0.005	2.60061205538689e-13	Monocyte	SIRPB1
1.4767662	4.798023	1	0.454	2.6268717628307e-13	Monocyte	SRGN
1.5701244	6.754857	0.348	0.016	2.79293737123593e-13	Monocyte	GNGT2
2.0343753	4.459999	0.261	0.005	3.61874681020826e-13	Monocyte	HLA-DQA2
2.2297104	4.494578	0.87	0.202	3.96620889215482e-13	Monocyte	KCNAB2
2.2323825	5.775785	0.348	0.016	3.97096211215163e-13	Monocyte	GIMAP4
3.5597651	5.123686	0.783	0.162	6.33211033655735e-13	Monocyte	ADAP2
3.7523813	6.346401	0.826	0.204	6.6747360059414e-13	Monocyte	TREM2
4.2188296	4.546233	0.522	0.05	7.50445420916221e-13	Monocyte	ARHGEF6
4.9716823	6.271732	0.304	0.011	8.84362864799973e-13	Monocyte	FCGR2C
5.2246256	5.039741	0.435	0.032	9.2935641690984e-13	Monocyte	ITGA4
5.8253614	4.236697	1	0.451	1.03621530280493e-12	Monocyte	TYMP
5.8803868	5.01874	1	0.424	1.04600322011712e-12	Monocyte	TNFAIP2
6.1517373	4.339719	0.783	0.164	1.09427103585148e-12	Monocyte	WIPF1
6.3790939	5.036505	0.391	0.024	1.13471323921295e-12	Monocyte	NFKBID
9.3568564	6.311284	0.913	0.316	1.66439761806261e-12	Monocyte	CD83
1.0953821	4.335893	0.652	0.093	1.94846585618779e-12	Monocyte	PYGL
1.2026120	5.815016	0.652	0.101	2.13920639267521e-12	Monocyte	TAGAP
1.2632965	5.425345	0.826	0.207	2.24715189688807e-12	Monocyte	NPL
1.5749612	5.824184	0.913	0.324	2.80154098258626e-12	Monocyte	C1QC
2.0347642	4.398522	0.913	0.294	3.61943869951615e-12	Monocyte	PIK3AP1
2.1920859	5.07233	1	0.515	3.89928257654286e-12	Monocyte	TNFAIP3
2.3900516	4.272857	0.87	0.233	4.25142383557005e-12	Monocyte	PPP1R18
2.5738614	3.546852	0.913	0.257	4.57838482099188e-12	Monocyte	SEMA4A
2.6187740	6.870967	0.348	0.019	4.65827525159022e-12	Monocyte	SIGLEC14
2.6777888	5.134704	0.435	0.034	4.76325088506535e-12	Monocyte	GBP5
3.4776132	6.243322	0.391	0.027	6.18597852762018e-12	Monocyte	ATP8B4
3.8283689	4.340609	0.826	0.188	6.80990272692082e-12	Monocyte	ALOX5
4.5826104	5.100156	0.87	0.249	8.15154740960039e-12	Monocyte	HLA-DQB1
5.0042774	5.276978	0.739	0.154	8.90160866240599e-12	Monocyte	CD52
5.5763587	3.222736	0.87	0.212	9.91922685991608e-12	Monocyte	CEP170
5.6755126	4.001132	0.957	0.324	1.00956019790321e-11	Monocyte	ARRB2
5.7855693	4.370439	0.348	0.019	1.0291370798235e-11	Monocyte	STARD8
6.5129367	3.773316	0.565	0.072	1.15852119648894e-11	Monocyte	VASH1
6.8417866	4.445522	0.957	0.382	1.21701701658509e-11	Monocyte	ZNF331
7.6011212	4.500103	0.957	0.408	1.35208745319808e-11	Monocyte	RGS1
8.1885642	4.298703	1	0.509	1.45658180217207e-11	Monocyte	ICAM1
8.5997590	4.219146	1	0.602	1.52972513421943e-11	Monocyte	HLA-DRA
8.9524417	6.451401	0.304	0.013	1.59246033780425e-11	Monocyte	LOC101927070
9.9190720	6.099705	0.304	0.013	1.76440453170269e-11	Monocyte	DCSTAMP
9.9190720	6.25669	0.304	0.013	1.76440453170269e-11	Monocyte	S100B
1.0872921	5.504712	1	0.65	1.93407534441693e-11	Monocyte	CTSL
1.1693843	4.69119	1	0.833	2.08010096176771e-11	Monocyte	IFI30
1.2415163	4.174541	0.826	0.207	2.20840930150704e-11	Monocyte	ICOSLG
1.3060623	4.57639	1	0.915	2.32322373177063e-11	Monocyte	CTSD
1.3478874	5.724767	0.304	0.013	2.3976221332785e-11	Monocyte	GGTA1P
1.3541652	4.137241	1	0.902	2.40878921072348e-11	Monocyte	CTSB
1.3901556	3.480868	0.826	0.188	2.47280884558503e-11	Monocyte	RASSF5

1.4887705	3.614666	1	0.939	2.64822505211332e-11	Monocyte	PSAP
1.5057836	5.49419	0.261	0.008	2.67848802222811e-11	Monocyte	TNFAIP8L2
1.8854167	4.444086	0.522	0.061	3.35377935239049e-11	Monocyte	MMP9
1.9431395	7.650227	0.913	0.377	3.45645670873589e-11	Monocyte	C1QB
1.9523211	6.649162	0.391	0.029	3.47278892864425e-11	Monocyte	SASH3
1.9553692	3.802464	1	0.878	3.47821087535608e-11	Monocyte	PLAUR
2.3846540	7.24011	0.739	0.167	4.24182255897468e-11	Monocyte	TNF
2.6591421	6.759923	0.913	0.374	4.73008196959759e-11	Monocyte	C1QA
2.9305895	3.424848	0.739	0.149	5.21293267465876e-11	Monocyte	DPYD
3.0778975	5.772034	0.348	0.021	5.47496421249948e-11	Monocyte	GPR65
3.8195477	5.068866	0.391	0.029	6.79421150397551e-11	Monocyte	MILR1
4.1465308	3.251604	1	0.851	7.37584901132491e-11	Monocyte	NFKBIA
4.3052997	3.977329	0.522	0.064	7.6582671075911e-11	Monocyte	CD36
4.3665416	6.165379	0.348	0.021	7.76720432951797e-11	Monocyte	HS3ST2
5.6986852	2.768794	0.522	0.058	1.01368212873929e-10	Monocyte	TRPV4
7.5007589	8.482741	0.826	0.252	1.33423500693143e-10	Monocyte	CCL4
7.7918552	4.154461	0.87	0.268	1.38601521613208e-10	Monocyte	CXCR4
9.5835941	5.085142	0.522	0.064	1.70472972424345e-10	Monocyte	AMPD3
1.4143796	4.952623	0.87	0.3	2.51589858271683e-10	Monocyte	ACP5
1.4639842	5.343771	0.391	0.032	2.60413514737488e-10	Monocyte	UBASH3B
1.5022936	5.825488	0.957	0.512	2.67227998987522e-10	Monocyte	LIPA
1.5699916	3.301865	0.826	0.199	2.79270114937577e-10	Monocyte	FAM20C
1.6874573	2.328183	1	0.997	3.0016491396651e-10	Monocyte	FTL
1.7173221	4.336026	0.696	0.13	3.05477261882869e-10	Monocyte	SLC23A2
1.7181320	3.411913	1	0.578	3.05621333464316e-10	Monocyte	VIM
1.8518438	2.829448	0.913	0.26	3.29405980174951e-10	Monocyte	FNBP1
1.8735223	3.71819	0.913	0.324	3.33262159898001e-10	Monocyte	QKI
1.9273289	2.911962	1	0.775	3.42833274565457e-10	Monocyte	LIMS1
2.1139516	3.256334	1	0.78	3.76029719520019e-10	Monocyte	CD74
2.2994320	6.430832	0.348	0.024	4.09022965181821e-10	Monocyte	ATP6V0D2
2.3310151	2.856646	0.913	0.271	4.14640982544863e-10	Monocyte	EMP3
2.3384611	3.106338	1	0.385	4.15965475539034e-10	Monocyte	PLXDC2
2.3811752	3.661872	0.87	0.281	4.23563449663796e-10	Monocyte	DOCK8
2.7112624	5.63889	0.348	0.024	4.8227936790975e-10	Monocyte	ADPRH
2.9754709	4.019559	0.609	0.101	5.29276768900474e-10	Monocyte	CMTM3
3.0274353	3.108749	0.435	0.042	5.38520205679072e-10	Monocyte	FLVCR2
3.3306180	3.445572	1	0.581	5.92450338723911e-10	Monocyte	METRNL
3.3735871	4.486511	0.522	0.069	6.00093687490705e-10	Monocyte	CHST11
3.4143804	3.193184	0.957	0.472	6.07349988429005e-10	Monocyte	RAB31
3.4490946	3.780552	0.957	0.589	6.13524948236775e-10	Monocyte	CD68
3.7630755	3.419194	1	0.538	6.69375880237985e-10	Monocyte	RNF130
4.2788443	6.705769	0.435	0.045	7.61120826323854e-10	Monocyte	LILRB3
4.3170658	4.638531	0.783	0.207	7.67919669459063e-10	Monocyte	SPHK1
4.4995394	2.952347	1	0.599	8.00378084998474e-10	Monocyte	COTL1
4.5465865	2.557783	1	0.894	8.08746815259071e-10	Monocyte	LITAF
4.8092619	4.32941	0.348	0.024	8.55471510123449e-10	Monocyte	FLI1
5.1649334	4.506888	0.783	0.191	9.18738358237102e-10	Monocyte	DDX3Y
6.4480951	6.724019	0.913	0.448	1.14698716051095e-09	Monocyte	IL1B
6.4686349	3.372206	1	0.459	1.15064079082202e-09	Monocyte	CCDC88A
7.0033953	2.768737	1	0.735	1.24576396806635e-09	Monocyte	TTYH3
7.2215418	3.292969	0.783	0.18	1.28456785876784e-09	Monocyte	CLEC2B
7.3351225	2.775805	0.652	0.111	1.30477159890244e-09	Monocyte	MEF2C
8.0113798	2.957611	0.957	0.353	1.42506425546273e-09	Monocyte	SLC16A3
8.2881675	2.849829	1	0.52	1.47429924670374e-09	Monocyte	LRP1
9.4062114	3.319425	1	0.804	1.67317689042611e-09	Monocyte	SOD2
1.0164211	4.775445	0.391	0.034	1.80800986016895e-09	Monocyte	SLC16A6
1.0337628	2.217247	1	0.995	1.83885744122942e-09	Monocyte	FTH1
1.0614259	4.926026	0.609	0.106	1.88806444143416e-09	Monocyte	RIN3

1.1301401	3.455061	0.652	0.122	2.01029322843036e-09	Monocyte	PLEKHO1
1.1989430	4.676577	0.957	0.467	2.13267981608651e-09	Monocyte	APOC1
1.3276154	2.506728	1	0.775	2.36156229670238e-09	Monocyte	GRB2
1.5861027	4.052563	0.957	0.432	2.82135950217255e-09	Monocyte	GK
1.5898272	4.085738	0.826	0.257	2.82798463046862e-09	Monocyte	SH2B3
1.6184828	4.371957	0.913	0.446	2.87895726967175e-09	Monocyte	GM2A
1.6272387	4.084728	0.609	0.109	2.89453227521392e-09	Monocyte	CD93
1.6608425	2.809364	0.609	0.106	2.95430676311969e-09	Monocyte	GAS7
1.7131034	5.730207	0.304	0.019	3.04726839417954e-09	Monocyte	CD300LB
1.7200036	3.855117	0.957	0.785	3.05954256891907e-09	Monocyte	ASAH1
2.0191593	4.989516	0.87	0.308	3.59168069354752e-09	Monocyte	THBD
2.4280160	3.064321	1	0.645	4.31895497847162e-09	Monocyte	CD81
2.8286624	4.420679	0.913	0.419	5.03162468241166e-09	Monocyte	ABL2
3.0174835	4.702693	0.304	0.019	5.36749976774616e-09	Monocyte	L3MBTL4-AS1
3.1199441	5.189018	0.391	0.037	5.5497567078164e-09	Monocyte	SFMBT2
3.4967325	4.577606	1	0.613	6.21998777301992e-09	Monocyte	CXCL8
3.8169047	3.530438	0.957	0.7	6.78951022781478e-09	Monocyte	PPT1
4.1666315	2.980444	0.957	0.61	7.4116041748046e-09	Monocyte	CREM
4.2570965	4.305459	0.435	0.048	7.57252342527882e-09	Monocyte	FCGR2B
4.3788472	3.858951	0.957	0.631	7.78909349438559e-09	Monocyte	TGFBI
4.6537827	3.745481	0.609	0.111	8.27814879440703e-09	Monocyte	PTGS1
4.7720418	3.903	0.957	0.589	8.48850811718949e-09	Monocyte	TIMP2
5.0523933	4.193898	0.783	0.239	8.98719735634328e-09	Monocyte	GPR137B
5.0700479	9.517898	0.826	0.329	9.01860130996718e-09	Monocyte	CCL3
5.2654694	3.44374	0.826	0.286	9.36621712279366e-09	Monocyte	PLAU
5.2926052	3.383868	0.348	0.027	9.41448627692156e-09	Monocyte	PHACTR1
5.6355026	4.084439	0.565	0.09	1.00244320334551e-08	Monocyte	OGFRL1
5.7716161	4.171572	0.87	0.401	1.02665508144285e-08	Monocyte	HLA-DPA1
6.3457696	3.22984	1	0.796	1.1287855135216e-08	Monocyte	SGK1
6.3968468	4.450679	0.739	0.204	1.13787112158054e-08	Monocyte	MERTK
7.1585805	2.41869	0.957	0.549	1.27336831253811e-08	Monocyte	HLA-DMA
7.3858395	3.470351	1	0.629	1.31379314451775e-08	Monocyte	NR4A2
7.7904969	4.061465	0.391	0.037	1.38577359193057e-08	Monocyte	SELPLG
7.8783854	3.550945	0.478	0.061	1.40140719562992e-08	Monocyte	MCTP1
7.9090429	3.615705	0.522	0.082	1.40686055339661e-08	Monocyte	SIGLEC10
7.9149440	2.415368	1	0.883	1.40791024495123e-08	Monocyte	NPC2
8.4261350	4.81608	0.522	0.077	1.49884090806039e-08	Monocyte	CHST15
9.5171168	5.446501	0.348	0.029	1.69290475061929e-08	Monocyte	P2RX7
9.5428772	4.231947	0.609	0.117	1.69748699835734e-08	Monocyte	TNFSF12
9.6792920	3.286705	0.957	0.594	1.72175246140876e-08	Monocyte	GNA13
9.9377551	4.603899	0.652	0.141	1.76772789194458e-08	Monocyte	EVI2A
1.0927255	4.068562	0.87	0.395	1.94374022825918e-08	Monocyte	SATB1
1.1063046	4.001912	0.913	0.538	1.96789478028096e-08	Monocyte	HLA-DPB1
1.1200315	3.497603	0.652	0.133	1.99231205686853e-08	Monocyte	GLIPR2
1.1722459	6.664829	0.261	0.013	2.08519107647765e-08	Monocyte	MEIKIN
1.2292521	5.304352	0.261	0.013	2.18659376261421e-08	Monocyte	CXorf21
1.2292521	5.239086	0.261	0.013	2.18659376261421e-08	Monocyte	RGS18
1.2345502	2.106056	1	0.899	2.1960179537922e-08	Monocyte	CTSS
1.2638676	2.674783	0.957	0.907	2.24816784916036e-08	Monocyte	GRN
1.2939071	3.052953	0.957	0.602	2.30160207994004e-08	Monocyte	HLA-DRB1
1.2971323	2.227037	1	0.865	2.30733895443101e-08	Monocyte	GLUL
1.3695080	3.457455	0.696	0.162	2.43608089122134e-08	Monocyte	CD109
1.4061756	6.83571	0.304	0.021	2.50130523141684e-08	Monocyte	LOC101927243
1.4646350	6.110454	0.304	0.021	2.60529278744169e-08	Monocyte	WDR17
1.4927371	3.49201	0.957	0.668	2.65528076618949e-08	Monocyte	SERPINB9
1.5254770	6.951251	0.304	0.021	2.71351855352152e-08	Monocyte	CLEC4E
1.5267556	2.716051	1	0.846	2.71579291302996e-08	Monocyte	NAMPT
1.5575656	3.762775	0.478	0.064	2.77059781103971e-08	Monocyte	RGL1

1.6900181	4.818988	0.696	0.172	3.00620428580325e-08	Monocyte	LPXN
2.3333119	1.975199	1	0.615	4.15049528925248e-08	Monocyte	LGALS1
2.5397892	4.554984	0.957	0.769	4.51777708860115e-08	Monocyte	LGMN
2.6438346	2.888727	0.913	0.387	4.70285302713678e-08	Monocyte	PDE4A
2.6765732	3.056077	1	0.599	4.76108849804773e-08	Monocyte	RAPGEF1
2.7610463	8.175238	0.652	0.156	4.91134922845308e-08	Monocyte	ALOX5AP
3.0326585	4.603754	0.304	0.021	5.39449310961045e-08	Monocyte	CMKLR1
3.1160866	3.884091	0.652	0.141	5.54289498329985e-08	Monocyte	TRPV2
3.4103888	2.602688	1	0.886	6.06639960720339e-08	Monocyte	IER3
3.5016977	5.891245	0.609	0.13	6.22882004033651e-08	Monocyte	HLA-DOA
3.7957225	1.473339	1	0.95	6.75183130995842e-08	Monocyte	OAZ1
3.8323934	4.477094	0.87	0.398	6.81706154641437e-08	Monocyte	DUSP2
3.8699646	2.807019	1	0.78	6.88389320780366e-08	Monocyte	ANXA5
3.8975535	2.504363	1	0.82	6.93296819495114e-08	Monocyte	GPX1
3.9391370	2.829202	0.783	0.247	7.00693706828014e-08	Monocyte	GAA
4.0541406	3.270426	0.913	0.546	7.21150534181299e-08	Monocyte	TPP1
4.1319164	4.94674	0.783	0.289	7.34985303902529e-08	Monocyte	GNB4
4.1780172	4.192249	0.565	0.109	7.43185708215511e-08	Monocyte	GPR34
4.2019869	4.408321	0.783	0.276	7.47449439462996e-08	Monocyte	NRP1
4.6440596	3.720114	0.913	0.485	8.26085335982921e-08	Monocyte	PTPRE
4.8853725	5.708411	0.348	0.032	8.69010070583742e-08	Monocyte	RAB3IL1
4.9560423	3.992556	0.87	0.406	8.81580810829356e-08	Monocyte	ARHGDIB
5.2798248	-3.77313	1	0.971	9.39175239198784e-08	Monocyte	KRT8
6.4921887	4.043558	0.609	0.127	1.15483052651725e-07	Monocyte	SNN
6.9023465	3.422844	0.739	0.207	1.22778939866655e-07	Monocyte	SAMHD1
7.0862997	7.461107	0.391	0.045	1.26051100671047e-07	Monocyte	FOLR2
7.9012377	-3.61258	1	0.963	1.4054721721902e-07	Monocyte	KRT18
9.3770337	3.541394	0.913	0.462	1.66798676738315e-07	Monocyte	PFKFB3
9.7851898	3.051009	0.957	0.703	1.74058957625495e-07	Monocyte	CTSC
1.0153413	5.877478	0.957	0.483	1.80608920322674e-07	Monocyte	SPP1
1.2552979	6.162529	0.261	0.016	2.23292407947557e-07	Monocyte	PRKCB
1.2902118	-3.9436	1	0.963	2.29502884046584e-07	Monocyte	ELF3
1.2904821	2.38468	0.957	0.793	2.29550962943038e-07	Monocyte	CAPG
1.3242554	-3.71743	1	0.968	2.35558558373736e-07	Monocyte	FXYD3
1.3290053	3.772682	0.913	0.517	2.36403463857303e-07	Monocyte	PLIN2
1.3667229	3.838642	0.826	0.318	2.43112681627772e-07	Monocyte	NFKBIE
1.3692238	5.872471	0.261	0.016	2.43557537606516e-07	Monocyte	TLR1
1.4619375	7.890637	0.826	0.387	2.60049459332846e-07	Monocyte	APOE
1.4709671	3.871558	0.87	0.39	2.61655642102744e-07	Monocyte	DAB2
1.4932576	5.671658	0.261	0.016	2.65620673186359e-07	Monocyte	LOC101927131
1.5233809	2.753167	0.696	0.172	2.70979002623001e-07	Monocyte	GAB2
1.5304919	3.730762	0.87	0.485	2.72243916030072e-07	Monocyte	SCPEP1
1.5593358	4.833335	0.261	0.016	2.77374657475702e-07	Monocyte	CD1D
1.5692258	3.983762	0.304	0.024	2.79133898950563e-07	Monocyte	C16orf54
1.5779122	3.027726	0.957	0.698	2.80679033000027e-07	Monocyte	GNAI2
1.7653978	-4.03149	0.957	0.966	3.14028973980881e-07	Monocyte	MUC1
2.1559960	4.234253	0.391	0.045	3.83508583024647e-07	Monocyte	RHOH
2.3708705	3.410247	0.565	0.106	4.21730450637069e-07	Monocyte	RASGRP3
2.4308328	4.814074	0.522	0.093	4.32396542038357e-07	Monocyte	BRE-AS1
2.5454267	2.687138	0.957	0.419	4.52780513250833e-07	Monocyte	CCL20
2.6481280	3.080574	0.826	0.268	4.71049022338435e-07	Monocyte	PLEKHG2
2.6717553	2.574288	0.435	0.056	4.75251841749741e-07	Monocyte	GLIPR1
2.7094350	2.64226	0.957	0.666	4.81954310760798e-07	Monocyte	GNS
2.7160861	2.015222	0.565	0.095	4.83137405395224e-07	Monocyte	SCARF1
2.7403875	4.548274	0.435	0.058	4.87460132017292e-07	Monocyte	IFFO1
3.1775610	4.724196	0.391	0.048	5.65224568226584e-07	Monocyte	VAV1
3.3771542	2.478398	0.261	0.016	6.00728204591788e-07	Monocyte	FLRT2
3.3940886	3.98111	0.348	0.034	6.03740497743731e-07	Monocyte	CACNA2D4

3.4328763	2.065942	1	0.873	6.10640050037941e-07	Monocyte	ATP6V0C
3.4548005	3.027777	0.957	0.69	6.14539922248557e-07	Monocyte	HEXA
3.4792980	-3.719	1	0.979	6.18897543596858e-07	Monocyte	EPCAM
3.5155023	2.027298	1	0.597	6.25337563236005e-07	Monocyte	RNF13
3.7578980	-2.76884	0.957	0.939	6.68454913384559e-07	Monocyte	DSTN
3.9880721	-4.80819	0.391	0.886	7.09398267726929e-07	Monocyte	DSP
4.4547167	3.586438	0.565	0.111	7.92405010369446e-07	Monocyte	GYPC
4.4600177	2.910816	0.913	0.727	7.9334795219923e-07	Monocyte	MGAT1
4.6083470	2.379223	0.522	0.088	8.19732766573215e-07	Monocyte	FMNL3
4.6088447	3.090045	0.783	0.26	8.19821309376966e-07	Monocyte	KYNU
4.8570868	3.284151	0.783	0.244	8.63978611153222e-07	Monocyte	RASGEF1B
5.0537300	2.629658	0.957	0.562	8.98957497938112e-07	Monocyte	CXCL2
5.2998591	3.572545	0.957	0.568	9.42738949723764e-07	Monocyte	RGS2
5.5762958	3.607062	0.478	0.074	9.91911500239432e-07	Monocyte	ARHGAP15
5.7603440	3.158015	0.913	0.475	1.02464999606849e-06	Monocyte	NINJ1
6.0358065	-4.77925	0.261	0.859	1.07364927289615e-06	Monocyte	MYO6
6.1003544	2.039376	1	0.96	1.0851310543074e-06	Monocyte	SAT1
6.3495096	6.838502	0.435	0.064	1.12945077780436e-06	Monocyte	IL23A
6.4038565	3.440755	0.783	0.257	1.13911800468691e-06	Monocyte	NIN
6.9747993	3.57304	0.783	0.281	1.24067729994842e-06	Monocyte	SLC43A3
7.0382772	5.135965	0.478	0.077	1.2519687528086e-06	Monocyte	FGR
7.8333493	3.171933	0.913	0.626	1.3933961799876e-06	Monocyte	MFSD1
7.9958840	4.407985	0.348	0.037	1.42230785972006e-06	Monocyte	AOAH
8.3333073	3.728704	0.739	0.249	1.48232871800012e-06	Monocyte	TCN2
8.9104075	2.806306	1	0.796	1.58498329020633e-06	Monocyte	CEBPB
8.9446432	3.675363	0.826	0.332	1.59107313445643e-06	Monocyte	SDSL
9.1899517	2.155163	1	0.817	1.63470862225882e-06	Monocyte	ATP1B3
9.6274602	3.04734	0.87	0.347	1.71253262267018e-06	Monocyte	ARAP1
1.0205038	2.752287	0.826	0.31	1.81527227899888e-06	Monocyte	ENG
1.0218541	2.455404	1	0.814	1.81767418921825e-06	Monocyte	PNRC1
1.0330221	2.315096	1	0.716	1.8375397497272e-06	Monocyte	PABPC4
1.1964743	1.93812	1	0.881	2.1282885650877e-06	Monocyte	CFLAR
1.3203049	2.997755	0.913	0.682	2.34855846122292e-06	Monocyte	CREG1
1.4095497	3.38307	0.565	0.125	2.50730708881731e-06	Monocyte	HMOX1
1.4357605	1.639949	1	0.844	2.55393094776121e-06	Monocyte	ATP6V1F
1.5848725	1.561525	0.957	0.406	2.81917125246521e-06	Monocyte	SEC14L1
1.6053206	-3.29974	0.826	0.947	2.85554433391519e-06	Monocyte	TPM1
1.6931942	2.035933	1	0.764	3.01185389660503e-06	Monocyte	FCGRT
1.7695328	5.833686	0.261	0.019	3.14764500076326e-06	Monocyte	LILRA3
1.8715051	3.777755	0.696	0.196	3.32903339348232e-06	Monocyte	CSGALNACT2
1.9039760	3.792129	0.826	0.369	3.38679261181698e-06	Monocyte	DMXL2
2.1512670	2.582813	0.913	0.467	3.8266739129638e-06	Monocyte	IGF2R
2.1547664	3.146597	0.87	0.371	3.83289862469434e-06	Monocyte	SOAT1
2.1563212	4.682111	0.348	0.04	3.83566416526089e-06	Monocyte	FAM78A
2.1701874	2.500632	0.957	0.607	3.86032940034688e-06	Monocyte	MYO9B
2.2944516	-4.23699	0.826	0.926	4.08137053503987e-06	Monocyte	KLF5
2.2951901	1.729756	0.957	0.767	4.08268419012046e-06	Monocyte	LAMP1
2.3621141	5.614303	0.304	0.029	4.20172866660122e-06	Monocyte	C10orf128
2.3621141	5.265078	0.304	0.029	4.20172866660122e-06	Monocyte	DPH1
2.4241723	1.49284	0.261	0.019	4.3121177340662e-06	Monocyte	COL8A2
2.5240463	-3.97691	0.739	0.928	4.48977360070262e-06	Monocyte	FHL2
2.5969673	3.444675	0.478	0.08	4.61948548472804e-06	Monocyte	BIN2
2.6627322	4.354109	0.696	0.218	4.7364681861154e-06	Monocyte	RAC2
2.8306562	-3.26178	0.957	0.936	5.03517136753283e-06	Monocyte	NQO1
2.9652436	-3.33289	0.783	0.923	5.27457537804668e-06	Monocyte	SMIM22
3.1353739	2.69374	0.913	0.493	5.57720313147512e-06	Monocyte	PRNP
3.2557990	2.926844	0.696	0.202	5.79141532856746e-06	Monocyte	NR3C1
3.7140292	2.044846	0.957	0.488	6.60651514908108e-06	Monocyte	AKR1B1

4.3604270	7.853136	0.522	0.114	7.75632762909249e-06	Monocyte	CCL18
4.8563332	3.948209	0.783	0.366	8.6384454991418e-06	Monocyte	S100A9
4.8612408	-2.59084	1	0.966	8.64717521498033e-06	Monocyte	IFI27
4.8914222	1.792254	0.565	0.117	8.70086186300004e-06	Monocyte	A2M
4.9343543	2.302602	0.739	0.239	8.77722954664295e-06	Monocyte	TMEM106A
4.9421742	3.909304	0.783	0.286	8.79113957988381e-06	Monocyte	CCRL2
4.9450692	2.462634	0.652	0.178	8.7962891339349e-06	Monocyte	GNA15
5.1132439	3.924281	0.739	0.263	9.09543828215585e-06	Monocyte	THEMIS2
5.4597825	3.385474	0.826	0.393	9.71186117637643e-06	Monocyte	RGS10
5.5774063	2.783713	0.87	0.403	9.92109039760386e-06	Monocyte	RHOG
5.9788119	3.347527	0.87	0.565	1.06351106780689e-05	Monocyte	AP1B1
6.0213437	3.618875	0.435	0.066	1.07107661883729e-05	Monocyte	FCMR
6.1718614	2.075809	0.391	0.053	1.09785072055034e-05	Monocyte	FAM26F
6.4116465	2.840985	0.783	0.3	1.14050369515107e-05	Monocyte	PI4K2A
6.5602227	3.392801	0.826	0.401	1.16693241715247e-05	Monocyte	ST3GAL1
6.9457354	-3.52856	0.478	0.881	1.2355074246706e-05	Monocyte	JUP
7.0028306	4.094533	0.826	0.401	1.24566351030503e-05	Monocyte	UCP2
7.1907258	2.807201	0.913	0.61	1.27908632277062e-05	Monocyte	LYN
7.1979321	3.919856	0.435	0.069	1.28036816258493e-05	Monocyte	DFNA5
7.5212780	1.727075	0.304	0.029	1.33788494633702e-05	Monocyte	TIAM1
7.5903350	2.046452	0.609	0.151	1.35016879717471e-05	Monocyte	MAF
7.6102660	2.577608	0.957	0.66	1.35371411779746e-05	Monocyte	REL
8.2256021	3.362218	0.826	0.438	1.46317011301202e-05	Monocyte	SLC7A7
8.2604907	-1.39695	1	0.979	1.46937609333734e-05	Monocyte	UBB
8.4049411	-4.55394	0.783	0.91	1.49507092878343e-05	Monocyte	TMC5
1.0104661	1.798203	1	0.83	1.79741713915879e-05	Monocyte	CTNNB1
1.0191851	-2.06691	1	0.995	1.81292663189012e-05	Monocyte	S100A6
1.0237404	2.592233	0.652	0.191	1.8210294302204e-05	Monocyte	ITGA5
1.0648067	-4.9515	0.043	0.761	1.89407832518842e-05	Monocyte	MAGI1
1.0872613	3.11748	0.913	0.565	1.93402047709179e-05	Monocyte	MICAL1
1.1744721	2.870479	0.913	0.682	2.08915109897175e-05	Monocyte	TFRC
1.1801210	2.809858	0.826	0.393	2.09919931374242e-05	Monocyte	SLC7A8
1.1888543	-3.77105	0.522	0.878	2.11473413393526e-05	Monocyte	STARD10
1.1904311	3.61015	0.826	0.332	2.11753893256057e-05	Monocyte	RILPL2
1.2408060	3.001277	0.87	0.531	2.2071458674644e-05	Monocyte	STX4
1.2890252	3.742221	0.652	0.194	2.29291805260865e-05	Monocyte	FMNL1
1.2953036	2.485827	1	0.626	2.30408616413991e-05	Monocyte	CXCL3
1.2972497	3.40819	0.739	0.271	2.30754778993905e-05	Monocyte	MFSD12
1.3434847	-3.72586	0.652	0.905	2.38979059615718e-05	Monocyte	EMP2
1.3550343	4.395439	0.652	0.218	2.41033510919624e-05	Monocyte	MALT1
1.3839740	5.024621	0.348	0.045	2.46181305667847e-05	Monocyte	IL18BP
1.5997678	1.886357	0.957	0.748	2.84566705599138e-05	Monocyte	SCARB2
1.7171276	2.13643	0.957	0.809	3.05442659452076e-05	Monocyte	BRI3
1.7497064	2.146414	0.87	0.448	3.11237783220987e-05	Monocyte	PRCP
1.8118806	1.883577	0.957	0.817	3.22297327497682e-05	Monocyte	GRINA
1.8536252	4.071029	0.652	0.178	3.29722860385576e-05	Monocyte	RNF166
1.9706776	5.069948	0.522	0.117	3.50544138898293e-05	Monocyte	SLC15A3
2.0281966	2.328243	0.957	0.668	3.60775624417269e-05	Monocyte	ATP13A3
2.0838772	-3.78585	0.696	0.889	3.70680089170296e-05	Monocyte	PRSS8
2.2394309	3.100729	0.826	0.329	3.98349984628039e-05	Monocyte	TK2
2.3338637	-3.02326	1	0.971	4.1514768776313e-05	Monocyte	S100P
2.3582033	2.747086	0.87	0.43	4.19477214141e-05	Monocyte	CPVL
2.3851911	-3.45684	1	0.955	4.24277797419232e-05	Monocyte	KRT19
2.4036802	3.06986	0.826	0.446	4.27566643890563e-05	Monocyte	ABCA1
2.4209117	3.04206	0.913	0.623	4.30631781733203e-05	Monocyte	IFNGR1
2.4599954	4.95592	0.652	0.191	4.37583995927394e-05	Monocyte	CTSZ
2.4702693	-2.96305	1	0.968	4.39411513846826e-05	Monocyte	TSPAN8
2.6087239	1.911595	0.87	0.546	4.64039821175892e-05	Monocyte	CORO1C

2.6107930	-3.50437	0.87	0.928	4.64407865868513e-05	Monocyte	MAL2
2.7099681	3.444208	0.609	0.17	4.82049134525397e-05	Monocyte	SDC2
2.7354326	4.134262	0.696	0.228	4.86578759761898e-05	Monocyte	ADAM8
2.9772896	-3.41844	0.783	0.889	5.29600279012693e-05	Monocyte	SLC44A4
3.0464547	2.80694	0.913	0.491	5.41903375728626e-05	Monocyte	EDEM1
3.1830576	2.462697	0.957	0.69	5.66202302732467e-05	Monocyte	BNIP3L
3.1993152	-2.22889	0.957	0.947	5.69094191037763e-05	Monocyte	SPINT2
3.2583521	-3.72472	0.609	0.891	5.79595675496172e-05	Monocyte	DSG2
3.3167978	4.653426	0.261	0.024	5.89992000604163e-05	Monocyte	PTPRO
3.3609306	3.405091	0.696	0.247	5.97842352141739e-05	Monocyte	RASSF4
3.4140864	6.831107	0.348	0.048	6.07297693478836e-05	Monocyte	IL1A
3.4265313	1.542732	1	0.825	6.09511396966262e-05	Monocyte	FXYD5
3.6721268	3.538446	0.652	0.202	6.53197916766747e-05	Monocyte	C1orf54
4.0287327	3.318875	0.87	0.568	7.16630980331329e-05	Monocyte	ATP6V1B2
4.1039029	2.19195	0.957	0.634	7.30002257245209e-05	Monocyte	KDM6B
4.1286940	-3.73072	0.739	0.865	7.34412091304312e-05	Monocyte	TSPAN13
4.2202795	-4.00279	0.435	0.841	7.50703318536083e-05	Monocyte	ERBB3
4.2806157	-3.50818	0.261	0.844	7.61435932720928e-05	Monocyte	ST6GALNAC1
4.5392953	-3.55416	0.957	0.958	8.07449862641289e-05	Monocyte	LGALS4
4.6796561	3.959447	0.565	0.143	8.32417240632671e-05	Monocyte	PLBD2
5.0827254	-1.54549	1	0.987	9.0411519564622e-05	Monocyte	RPL18A
5.1155310	2.4348	0.913	0.618	9.09950669293357e-05	Monocyte	ZYX
5.2963650	4.761222	0.435	0.082	9.42117418163777e-05	Monocyte	S100A8
5.4414307	-3.10871	0.609	0.894	9.67921703297273e-05	Monocyte	PERP
5.7032329	3.436963	0.522	0.119	0.000101449	Monocyte	PPM1M
5.8925007	-4.73598	0.913	0.923	0.000104816	Monocyte	TACSTD2
5.9000380	-3.95526	0.261	0.801	0.00010495	Monocyte	GNG12
6.6482578	1.778218	0.957	0.586	0.000118259	Monocyte	BST2
6.7038239	1.202597	1	0.833	0.000119248	Monocyte	RNF149
6.8390696	2.45114	0.913	0.692	0.000121653	Monocyte	SLC31A1
6.8698662	3.196609	0.696	0.231	0.000122201	Monocyte	SLC36A1
6.9328981	2.882621	0.826	0.424	0.000123322	Monocyte	PTPN6
7.7456652	3.095454	0.826	0.393	0.00013778	Monocyte	AGAP3
8.2706509	-4.85381	0.391	0.833	0.000147118	Monocyte	MLPH
8.3223981	-3.1671	0.652	0.897	0.000148039	Monocyte	RAB25
8.5755089	-3.82367	0.913	0.894	0.000152541	Monocyte	EFNA1
8.9800371	2.139732	1	0.859	0.000159737	Monocyte	BTG1
9.0382275	-1.38907	1	0.979	0.000160772	Monocyte	RPL18
9.2369173	-2.78138	1	0.971	0.000164306	Monocyte	HSPB1
9.5767061	-3.39544	0.696	0.87	0.00017035	Monocyte	CTTN
9.7805576	-3.48011	0.348	0.82	0.000173977	Monocyte	NET1
9.9522746	2.172792	0.87	0.43	0.000177031	Monocyte	IRF8
1.0360342	3.95957	0.304	0.037	0.00018429	Monocyte	HGF
1.0882136	-3.15794	1	0.963	0.000193571	Monocyte	GPX2
1.0883108	1.066131	0.739	0.257	0.000193589	Monocyte	SERPING1
1.0914614	3.592896	0.391	0.064	0.000194149	Monocyte	PTPN22
1.2097837	4.044941	0.348	0.05	0.000215196	Monocyte	RFTN1
1.2180839	-4.00707	0.348	0.817	0.000216673	Monocyte	SDC1
1.2374395	-3.49169	0.391	0.825	0.000220116	Monocyte	LLGL2
1.2451594	-2.6248	0.913	0.923	0.000221489	Monocyte	S100A16
1.3040998	4.100833	0.348	0.05	0.000231973	Monocyte	CELF6
1.3290292	2.697118	0.435	0.082	0.000236408	Monocyte	FXYD6
1.5061723	2.920229	0.609	0.17	0.000267918	Monocyte	FRMD4A
1.5127633	-4.41155	0.913	0.92	0.00026909	Monocyte	MUC13
1.6195091	2.711011	0.565	0.154	0.000288078	Monocyte	AXL
1.6672610	2.820211	0.696	0.244	0.000296572	Monocyte	SLC7A5
1.6967911	-4.71512	0.522	0.865	0.000301825	Monocyte	BCAS1
1.7484237	3.685976	0.783	0.324	0.00031101	Monocyte	VMO1

1.7999827	1.381363	0.783	0.289	0.000320181	Monocyte	RDX
1.8068556	-2.13189	1	0.936	0.000321403	Monocyte	RRBP1
1.8495772	-3.47079	0.739	0.867	0.000329003	Monocyte	PDLIM1
1.8764653	4.296358	0.478	0.106	0.000333786	Monocyte	GAPLINC
1.9209321	1.653377	0.304	0.037	0.000341695	Monocyte	CD38
2.0168825	1.357951	1	0.905	0.000358763	Monocyte	ATP6V0B
2.0406456	-3.36644	0.957	0.897	0.00036299	Monocyte	OCIAD2
2.0747674	-2.00815	0.957	0.931	0.00036906	Monocyte	KDELR2
2.0949235	3.855901	0.565	0.143	0.000372645	Monocyte	BCL11A
2.1075221	-1.29895	1	0.987	0.000374886	Monocyte	PTMA
2.1377424	2.343112	0.87	0.623	0.000380262	Monocyte	PEA15
2.4809040	2.08704	0.87	0.398	0.000441303	Monocyte	TBXAS1
2.5131935	3.048971	0.696	0.257	0.000447047	Monocyte	SSH1
2.5508205	4.928584	0.435	0.09	0.00045374	Monocyte	SYTL3
2.6038142	1.814419	0.957	0.724	0.000463166	Monocyte	RNH1
2.6098813	-1.25126	1	0.992	0.000464246	Monocyte	RPS27
2.6263844	-4.04831	0.435	0.814	0.000467181	Monocyte	EHF
2.8251327	2.201838	0.478	0.103	0.000502535	Monocyte	MRAS
2.8383615	-2.47664	1	0.955	0.000504888	Monocyte	CYSTM1
2.9562484	-4.1267	0.913	0.944	0.000525857	Monocyte	CLDN3
3.2131905	5.853644	0.391	0.074	0.000571562	Monocyte	ITGAL
3.3311740	2.773738	0.609	0.183	0.000592549	Monocyte	FERMT3
3.5178717	3.296961	0.739	0.284	0.000625759	Monocyte	ANPEP
3.6251630	4.859946	0.435	0.09	0.000644844	Monocyte	GAL3ST4
3.8006402	2.793348	0.652	0.218	0.000676058	Monocyte	CPNE2
3.8520247	-3.50235	0.652	0.859	0.000685198	Monocyte	FAM3B
3.9193837	-3.41384	0.174	0.775	0.00069718	Monocyte	CAPN5
3.9700031	2.029825	0.957	0.655	0.000706184	Monocyte	SMS
3.9849081	3.951191	0.348	0.053	0.000708835	Monocyte	BCL2
4.0537579	1.70631	0.957	0.793	0.000721082	Monocyte	ATP6AP2
4.0584630	-3.8436	0.043	0.692	0.000721919	Monocyte	PRR15
4.4232332	-2.40095	1	0.958	0.000786805	Monocyte	CD24
4.8862714	2.890724	0.652	0.218	0.00086917	Monocyte	PARVB
4.9304164	2.988623	0.826	0.467	0.000877022	Monocyte	ZFYVE16
5.3677682	2.372166	0.739	0.284	0.000954819	Monocyte	GAS6
5.3712847	1.768611	1	0.817	0.000955444	Monocyte	ARPC4
5.3869646	3.919529	0.348	0.056	0.000958233	Monocyte	ZFY
5.6415083	1.823445	0.783	0.353	0.001003512	Monocyte	FLNA
5.7240286	-4.15114	0.304	0.796	0.00101819	Monocyte	CGN
5.7474266	2.123814	0.87	0.552	0.001022352	Monocyte	SH3BGRL
6.1380690	2.911604	0.696	0.255	0.00109184	Monocyte	IGFLR1
6.3977708	2.51337	0.87	0.536	0.001138035	Monocyte	ELL2
6.5157443	-3.2047	0.304	0.769	0.001159021	Monocyte	AP1M2
6.5422870	1.486122	1	0.748	0.001163742	Monocyte	CTSH
6.5968511	2.34431	0.478	0.106	0.001173448	Monocyte	RASA3
6.7118467	2.036722	0.87	0.496	0.001193903	Monocyte	MAN2B1
7.0806709	3.350504	0.739	0.34	0.00125951	Monocyte	LHFPL2
7.0820883	3.344112	0.87	0.708	0.001259762	Monocyte	PLD3
7.3052615	2.915658	0.565	0.17	0.00129946	Monocyte	IL6
7.8425883	-1.47518	1	0.995	0.00139504	Monocyte	RPL7
7.9967166	-2.88205	0.957	0.958	0.001422456	Monocyte	CLDN4
8.2983816	-4.18289	0.348	0.785	0.001476116	Monocyte	MYH14
8.3901031	-2.5081	1	0.928	0.001492432	Monocyte	SPTBN1
8.6259523	3.204595	0.522	0.141	0.001534384	Monocyte	SYNE1
8.7762624	-3.48588	0.522	0.841	0.001561122	Monocyte	OPTN
9.5466628	1.670743	1	0.78	0.00169816	Monocyte	RNASET2
9.5653673	-3.0549	0.478	0.825	0.001701488	Monocyte	ICA1
9.9947199	1.631637	1	0.846	0.001777861	Monocyte	ATP6AP1

1.0170865	3.28088	0.696	0.268	0.001809193	Monocyte	SETDB2
1.0685759	-4.13293	0.565	0.825	0.001900783	Monocyte	LMO7
1.1048058	2.933649	0.826	0.446	0.001965229	Monocyte	PLEKHM2
1.1110557	1.10633	1	0.987	0.001976346	Monocyte	ACTB
1.1743259	-3.86489	0.391	0.788	0.002088891	Monocyte	TMPRSS4
1.1971067	1.879941	1	0.806	0.002129414	Monocyte	NFKBIZ
1.1971955	1.792013	0.913	0.443	0.002129571	Monocyte	CXCL1
1.1985894	2.250925	0.652	0.233	0.002132051	Monocyte	SEPT6
1.2492885	2.645892	0.783	0.475	0.002222234	Monocyte	ADRBK2
1.2737673	2.006562	0.913	0.655	0.002265777	Monocyte	USF2
1.2913540	-3.16859	0.13	0.708	0.002297061	Monocyte	PTK2
1.3253512	1.380321	1	0.889	0.002357535	Monocyte	S100A4
1.3409682	1.771975	0.87	0.491	0.002385314	Monocyte	TLN1
1.3424598	3.762758	0.652	0.252	0.002387968	Monocyte	PLEKHO2
1.3612891	2.922892	0.826	0.459	0.002421461	Monocyte	GLA
1.3859868	1.779367	0.913	0.581	0.002465393	Monocyte	PTMS
1.4081007	-3.61794	0.826	0.928	0.00250473	Monocyte	LSM8
1.4272966	1.753407	1	0.846	0.002538875	Monocyte	CHMP1B
1.4497302	-3.11127	0.391	0.822	0.00257878	Monocyte	ABLIM1
1.4561570	2.88139	0.739	0.332	0.002590212	Monocyte	USP36
1.4787349	-4.67604	0.609	0.87	0.002630374	Monocyte	MET
1.6068098	2.863246	0.826	0.549	0.002858193	Monocyte	NFKB1
1.6140262	2.450258	0.826	0.52	0.00287103	Monocyte	PGD
1.6247488	1.827904	0.913	0.735	0.002890103	Monocyte	TCIRG1
1.7115950	-2.94844	0.565	0.873	0.003044585	Monocyte	TPD52
1.7830072	-3.13023	0.609	0.841	0.003171613	Monocyte	CREB3L1
1.9284815	2.943269	0.87	0.65	0.003430383	Monocyte	SLC43A2
1.9485609	3.537862	0.696	0.297	0.0034661	Monocyte	STAT5A
1.9518205	2.582942	0.957	0.666	0.003471898	Monocyte	B4GALT1
1.9948370	1.7993	0.957	0.817	0.003548416	Monocyte	CD44
2.0048568	-3.38701	0.652	0.836	0.003566239	Monocyte	PTPRF
2.0831246	-3.80236	0.13	0.703	0.003705462	Monocyte	LAMC2
2.1497456	-2.24158	0.87	0.912	0.003823968	Monocyte	TOB1
2.2565814	1.12633	1	0.918	0.004014007	Monocyte	PPP1R15A
2.3769661	-2.9124	0.391	0.796	0.004228147	Monocyte	TJP1
2.4212293	-3.69772	0.174	0.698	0.004306883	Monocyte	ENAH
2.4389709	1.657428	0.913	0.668	0.004338442	Monocyte	DPP7
2.5353071	-2.94157	0.739	0.854	0.004509804	Monocyte	CDH1
2.6181230	-2.24043	0.739	0.857	0.004657117	Monocyte	MARCKSL1
2.6697027	3.162911	0.739	0.393	0.004748867	Monocyte	CPM
2.6741270	3.395042	0.739	0.337	0.004756737	Monocyte	MOB3A
2.7557401	-2.94862	0.391	0.836	0.004901911	Monocyte	BAIAP2L1
2.8394657	1.032999	1	0.926	0.005050842	Monocyte	GABARAP
2.8601062	1.679703	0.87	0.499	0.005087557	Monocyte	RGCC
2.8884288	3.285061	0.652	0.268	0.005137937	Monocyte	SNAI1
2.8999568	2.3195	0.522	0.146	0.005158443	Monocyte	PDGFB
2.9028124	-4.23497	0.435	0.801	0.005163523	Monocyte	CYP3A5
2.9826579	4.175299	0.565	0.178	0.005305552	Monocyte	RELT
3.0113383	-3.27944	0.304	0.759	0.005356569	Monocyte	NR2F6
3.0994380	2.581738	0.652	0.22	0.00551328	Monocyte	IFI16
3.1405906	-5.03852	0.304	0.775	0.005586483	Monocyte	PLAC8
3.1410942	-2.77181	0.739	0.891	0.005587378	Monocyte	SPATS2L
3.1674496	-2.52918	0.565	0.841	0.005634259	Monocyte	TUBB4B
3.1720989	-2.5176	0.348	0.814	0.00564253	Monocyte	ASL
3.1990232	-3.11342	0.522	0.812	0.005690422	Monocyte	DDR1
3.2002921	-1.37878	1	0.934	0.00569268	Monocyte	EDF1
3.2031867	-3.4108	0.478	0.79	0.005697829	Monocyte	MPST
3.2354270	-3.52474	0.348	0.772	0.005755178	Monocyte	RASEF

3.2616495	3.389664	0.609	0.228	0.005801822	Monocyte	INPP5D
3.3042922	2.31106	0.87	0.557	0.005877675	Monocyte	ARHGAP18
3.3471276	-4.21108	0.87	0.92	0.005953871	Monocyte	TSPAN1
3.3789772	-2.64024	0.913	0.493	0.006010525	Monocyte	JCHAIN
3.4210369	2.032739	0.696	0.284	0.006085341	Monocyte	AKNA
3.5350387	-3.12097	0.217	0.727	0.006288127	Monocyte	B3GNT3
3.5908462	-2.60621	0.696	0.854	0.006387397	Monocyte	LIMA1
3.5997466	4.925703	0.348	0.066	0.006403229	Monocyte	NFAM1
3.6390593	2.377369	0.739	0.35	0.006473159	Monocyte	KIAA0930
3.6744762	1.638752	0.957	0.782	0.006536158	Monocyte	YWHAH
3.6914423	1.89663	1	0.729	0.006566338	Monocyte	BLOC1S6
3.8772948	-3.1575	0.609	0.82	0.006896932	Monocyte	KRT10
3.9265360	-3.97997	0.087	0.653	0.006984522	Monocyte	ARFGEF3
3.9288189	-3.04891	0.304	0.769	0.006988583	Monocyte	EPS8L3
3.9780510	2.404471	0.783	0.408	0.007076157	Monocyte	GGA1
4.0379695	-3.9337	0.174	0.698	0.00718274	Monocyte	GSDMB
4.1224837	3.031783	0.522	0.149	0.007333074	Monocyte	ITPR1
4.2340959	-3.63823	0.348	0.761	0.00753161	Monocyte	CXADR
4.4765968	-1.60312	1	0.979	0.00796297	Monocyte	GSTP1
4.5050456	3.130012	0.739	0.401	0.008013575	Monocyte	NAGA
5.0489157	1.886037	0.913	0.679	0.008981011	Monocyte	CYTH1
5.0737946	-2.74989	0.13	0.687	0.009025266	Monocyte	LRP5
5.1782454	-4.83627	0.261	0.775	0.009211063	Monocyte	COL16A1
5.3426637	2.271802	0.87	0.533	0.00950353	Monocyte	FNIP2
5.5805863	3.3965	0.261	0.034	0.009926747	Monocyte	ZMIZ1-AS1
5.7969493	3.477507	0.478	0.119	0.010311614	Monocyte	ARHGEF40
5.8495620	-3.26955	0.217	0.719	0.010405201	Monocyte	MFSD6
5.9374760	2.965777	0.826	0.634	0.010561582	Monocyte	WTAP
6.0540401	3.080175	0.348	0.064	0.010768927	Monocyte	CALHM2
6.1590317	1.200683	1	0.992	0.010955686	Monocyte	TMSB4X
6.2288824	-3.99839	0.13	0.66	0.011079936	Monocyte	POF1B
6.2430050	-3.43549	0.783	0.859	0.011105057	Monocyte	PLA2G10
6.3394121	1.223138	1	0.836	0.011276546	Monocyte	BHLHE40
6.3507521	1.220324	0.957	0.769	0.011296718	Monocyte	TALDO1
6.4000021	-4.82333	0.348	0.753	0.011384324	Monocyte	AGR3
6.5420935	-2.84954	0.217	0.737	0.011637076	Monocyte	TMEM54
6.6543366	-2.92306	0.304	0.769	0.011836734	Monocyte	FAM221A
6.7940717	-2.38576	0.826	0.875	0.012085295	Monocyte	TSTA3
6.9812028	1.484906	0.826	0.377	0.012418164	Monocyte	RPS4Y1
7.1065345	-3.86013	0.304	0.74	0.012641104	Monocyte	ITGA2
7.1188241	4.067177	0.391	0.088	0.012662964	Monocyte	TMC8
7.1207496	-2.14448	0.739	0.865	0.012666389	Monocyte	ARPC1A
7.3044726	3.207757	0.739	0.353	0.012993196	Monocyte	IL1RN
7.5476878	3.656195	0.565	0.21	0.013425827	Monocyte	CCDC69
7.6670940	3.212483	0.739	0.451	0.013638227	Monocyte	ALCAM
7.9908280	3.185427	0.783	0.448	0.014214085	Monocyte	CLN8
8.0874689	3.889181	0.348	0.066	0.01438599	Monocyte	MB21D2
8.0922951	-2.73757	0.87	0.862	0.014394575	Monocyte	ITGA6
8.5211448	-1.29372	1	0.973	0.015157412	Monocyte	RPL30
8.6413472	-3.77841	0.478	0.772	0.015371228	Monocyte	SOX9
8.6739046	-3.00174	0.348	0.777	0.015429142	Monocyte	ASAP2
8.7809005	1.50459	0.913	0.586	0.015619466	Monocyte	RB1
8.8849179	1.999146	1	0.918	0.015804492	Monocyte	TIMP1
9.0706145	-3.62799	0	0.581	0.016134809	Monocyte	DLG3
9.1593448	-2.51542	0.478	0.814	0.016292643	Monocyte	NOSIP
9.3036153	-3.1145	0.522	0.806	0.016549271	Monocyte	ABHD17C
9.4243543	1.835263	0.913	0.788	0.016764041	Monocyte	ATP6V0D1
9.4713813	-3.44043	0.304	0.724	0.016847693	Monocyte	RHPN2

9.5692680	-2.27119	0.87	0.881	0.017021814	Monocyte	SF3B6
9.8957595	1.102294	0.913	0.849	0.017602577	Monocyte	ARPC5
9.9880773	2.227478	0.87	0.523	0.017766792	Monocyte	STAT2
1.0145615	2.924461	0.652	0.273	0.018047021	Monocyte	FES
1.0242385	-3.43903	0.435	0.788	0.018219155	Monocyte	FUT3
1.0490827	3.466765	0.261	0.037	0.018661084	Monocyte	SEMA6B
1.0513853	-2.95366	0.826	0.92	0.018702043	Monocyte	TES
1.0518560	-2.54249	0.565	0.82	0.018710415	Monocyte	GALNT3
1.0726062	-3.18484	0.609	0.801	0.019079521	Monocyte	PPIC
1.0733874	4.533891	0.478	0.13	0.019093417	Monocyte	WDR7
1.0758195	2.282951	0.783	0.435	0.019136678	Monocyte	TET2
1.0897762	3.419838	0.478	0.13	0.01938494	Monocyte	SH3TC1
1.1205523	1.03586	1	0.902	0.019932385	Monocyte	CSTB
1.1593992	-1.85091	0.913	0.897	0.020623393	Monocyte	MRPS21
1.2011547	-4.1654	0.739	0.854	0.021366141	Monocyte	ID1
1.2380144	-2.47848	0.478	0.79	0.022021801	Monocyte	NDFIP2
1.2723964	3.830689	0.304	0.053	0.022633387	Monocyte	DYSF
1.3000517	4.024961	0.304	0.053	0.023125321	Monocyte	HIC1
1.3518026	-1.43035	1	0.981	0.024045866	Monocyte	CHCHD2
1.3579048	-3.42781	0.087	0.623	0.024154412	Monocyte	NOSTRIN
1.3614259	1.599259	1	0.939	0.024217044	Monocyte	DUSP1
1.3799126	2.584283	0.652	0.255	0.024545886	Monocyte	PRDM1
1.4022689	-3.62883	0.565	0.859	0.02494356	Monocyte	ASS1
1.4156838	-1.26499	1	0.963	0.025182184	Monocyte	NDUFA13
1.4447863	-2.73448	0.261	0.703	0.02569986	Monocyte	ZFAND1
1.4467842	2.139741	0.913	0.557	0.025735398	Monocyte	MAP2K1
1.4524686	-3.009	0.174	0.684	0.025836512	Monocyte	IRF6
1.4594468	-2.695	0.478	0.817	0.025960641	Monocyte	C1orf106
1.4634442	-2.87246	0.304	0.729	0.026031747	Monocyte	CHMP4C
1.5628292	2.820829	0.348	0.069	0.027799607	Monocyte	GNG2
1.5631189	4.250522	0.478	0.141	0.027804759	Monocyte	BMP2K
1.6314237	-2.20755	0.957	0.897	0.029019765	Monocyte	TMBIM1
1.6457209	-2.87382	0.348	0.772	0.029274083	Monocyte	MUC3A
1.6768931	-3.48945	0.174	0.674	0.029828575	Monocyte	SH2D4A
1.6836477	1.3925	1	0.891	0.029948726	Monocyte	MCL1
1.6884096	-1.39391	1	0.958	0.030033431	Monocyte	KRTCAP2
1.7253642	3.050624	0.696	0.366	0.030690779	Monocyte	NABP1
1.7343258	-2.80112	0.304	0.743	0.030850187	Monocyte	HDHD3
1.7604617	-3.27356	0.304	0.735	0.031315094	Monocyte	AKR1C3
1.7811951	1.649386	0.87	0.653	0.031683899	Monocyte	BID
1.7871804	-3.27546	0.217	0.674	0.031790365	Monocyte	HIP1R
1.7906615	-2.13015	0.739	0.865	0.031852288	Monocyte	PSMA4
1.8360793	-2.86151	0.261	0.737	0.032660179	Monocyte	SLC2A1
1.8382145	3.069425	0.435	0.114	0.03269816	Monocyte	ATG16L2
1.8878661	-2.73924	0.304	0.769	0.033581363	Monocyte	TNFSF10
1.8920976	2.932356	0.609	0.244	0.033656633	Monocyte	RGS19
1.9200243	-1.64602	0.957	0.918	0.034153393	Monocyte	NDUFS6
1.9642689	1.97621	0.826	0.565	0.034940415	Monocyte	FOXN3
2.0082762	-3.57655	0.261	0.708	0.035723218	Monocyte	MLLT4
2.0085824	3.691818	0.609	0.257	0.035728664	Monocyte	SGPP1
2.0179076	-3.82362	0.522	0.817	0.035894542	Monocyte	CEACAM1
2.0290817	1.695777	0.957	0.769	0.036093305	Monocyte	FABP5
2.0564147	2.216052	0.783	0.499	0.036579505	Monocyte	WARS
2.0722320	-3.28304	0.304	0.727	0.036860864	Monocyte	TST
2.0921028	-2.09524	0.739	0.897	0.037214326	Monocyte	LRP10
2.1309067	2.333896	0.391	0.09	0.037904569	Monocyte	ARMCX1
2.1883710	-3.05324	0.13	0.629	0.038926744	Monocyte	YAP1
2.2905037	1.087589	1	0.905	0.04074348	Monocyte	SDCBP

2.3072607	-2.82835	0.913	0.862	0.041041554	Monocyte	GMDS
2.3351157	1.537786	0.87	0.684	0.04153704	Monocyte	WNK1
2.3819587	-4.51897	0.435	0.764	0.042370282	Monocyte	GCNT3
2.4386541	-2.72634	0.565	0.822	0.04337878	Monocyte	PARD6B
2.4951836	-3.25802	0.043	0.597	0.044384326	Monocyte	EPN2
2.5476156	-2.51245	0.217	0.743	0.045316987	Monocyte	ERBB2
2.5922405	-2.69246	0.783	0.859	0.046110775	Monocyte	RAB11FIP1
2.5939186	2.209351	0.783	0.472	0.046140625	Monocyte	GALM
2.6006724	-2.78954	0.435	0.782	0.046260762	Monocyte	LAPTM4B
2.7640708	-3.06784	0.652	0.801	0.049167292	Monocyte	PRR15L
2.7790206	2.513721	0.739	0.369	0.04943322	Monocyte	SWAP70
