# Supplementary material for: A prognostic model based on autophagy-and senescence-related genes for gastric cancer: implications for immunotherapy and personalized treatment
Source: Front Oncol. 2025 Mar 20;15:1509771. doi: 10.3389/fonc.2025.1509771 (PMC11965130; doi:10.3389/fonc.2025.1509771)
Supplement: Supplementary file 4 [file DataSheet4.docx]

Supplementary Material

# Supplementary Table

**Supplementary Table 1. Baseline data of GC patients with complete clinical information in this study.**

| **Characteristic** |  | **TCGA cohort** | **GEO cohort** |
| --- | --- | --- | --- |
| **No. of patients** |  | N=332 | N=298 |
| **Survival status, n(%)** | Alive | 197 (59.3%) | 146 (49.0%) |
|  | Dead | 135 (40.7%) | 152 (51.0%) |
| **Age, n(%)** | <65 | 150(45.2%) | 161(54.0%) |
|  | ≥65 | 182(54.8%) | 137(46.0%) |
| **Gender, n(%)** | Female | 120 (36.1%) | 101 (33.9%) |
|  | Male | 212 (63.9%) | 197 (66.1%) |
| **T Stage, n(%)** | T1 | 14 (4.22%) | 0 (0.00%) |
|  | T2 | 70 (21.1%) | 186 (62.4%) |
|  | T3 | 158 (47.6%) | 91 (30.5%) |
|  | T4 | 90 (27.1%) | 21 (7.05%) |
| **N Stage, n(%)** | N0 | 104 (31.3%) | 38 (12.8%) |
|  | N1 | 89 (26.8%) | 130 (43.6%) |
|  | N2 | 67 (20.2%) | 79 (26.5%) |
|  | N3 | 72 (21.7%) | 51 (17.1%) |
| **M Stage, n(%)** | M0 | 311 (93.7%) | 271 (90.9%) |
|  | M1 | 21 (6.33%) | 27 (9.06%) |
| **Clinical Stage, n(%)** | Stage I | 43 (13.0%) | 30 (10.1%) |
|  | Stage II | 109 (32.8%) | 96 (32.2%) |
|  | Stage III | 147 (44.3%) | 95 (31.9%) |
|  | Stage IV | 33 (9.94%) | 77 (25.8%) |
| **Risk, n(%)** | High | 167 (50.3%) | 205 (68.8%) |
|  | Low | 165 (49.7%) | 93 (31.2%) |

# Supplementary Figures

**
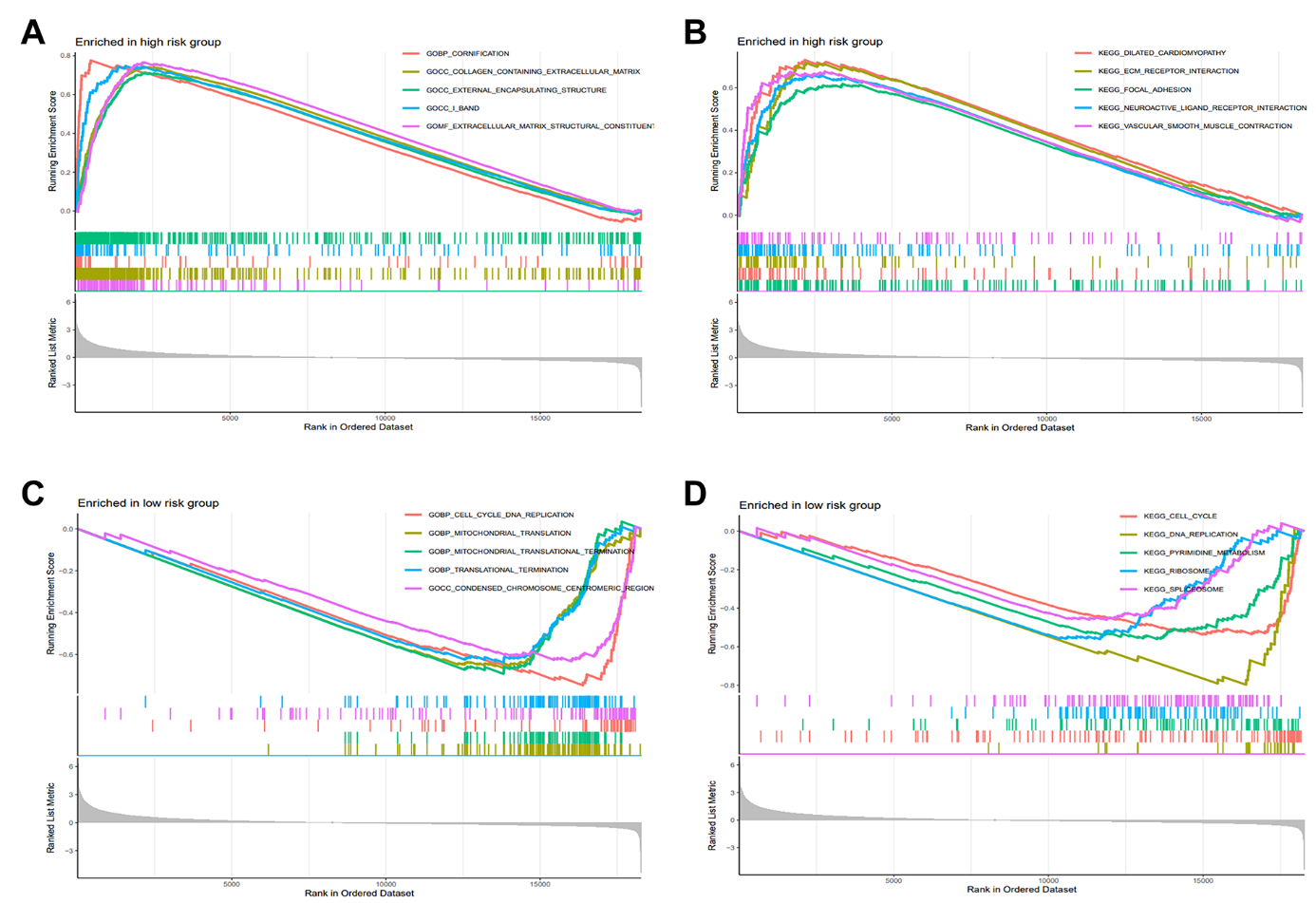
**

**Supplementary Figure 1.** GSEA analysis of risk model. (A) Enrichment analysis in TCGA cohort in high-risk group. (B) Enrichment analysis in GEO cohort in high-risk group. (C) Enrichment analysis in TCGA cohort in low-risk group. (D) Enrichment analysis in GEO cohort in low-risk group.


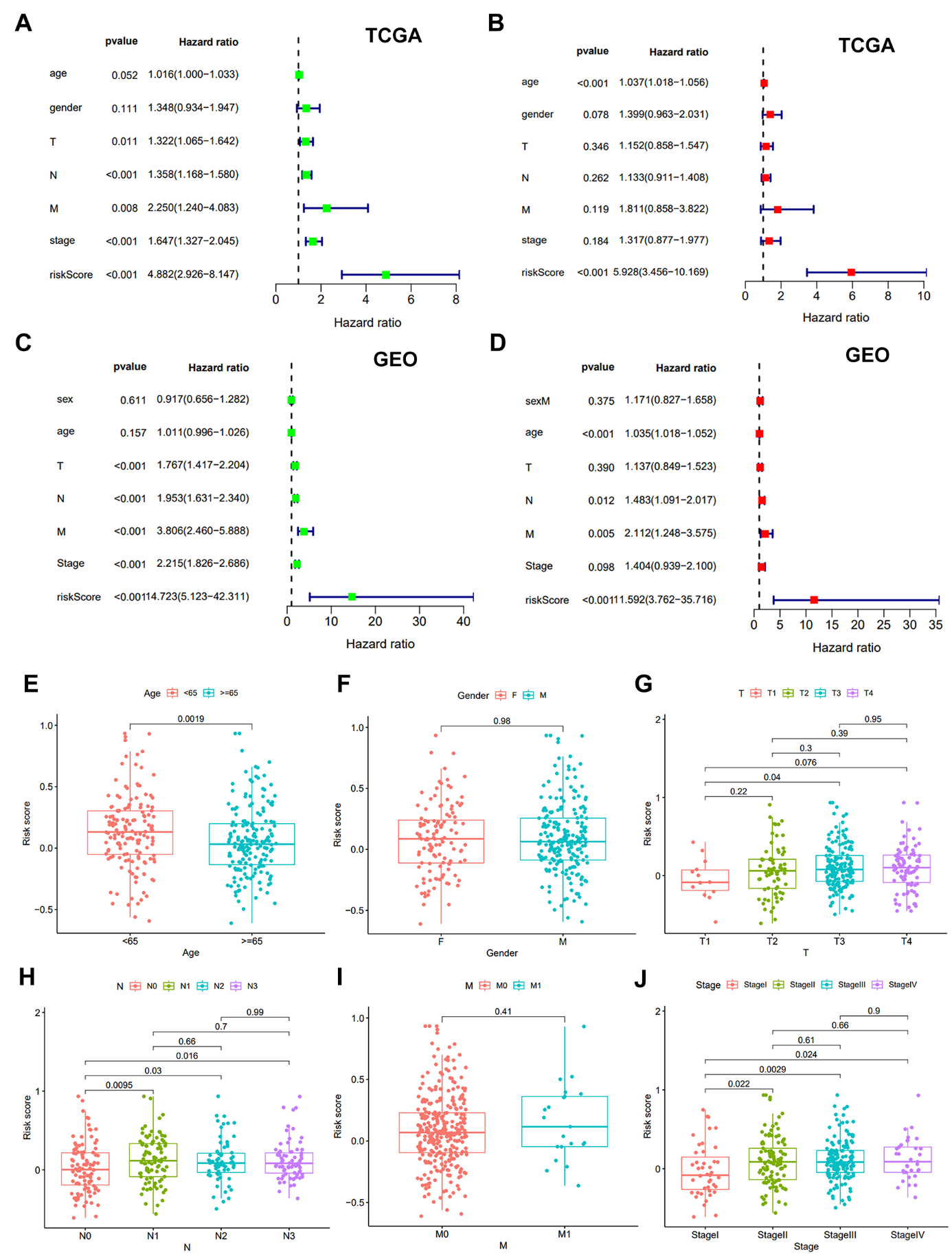


**Supplementary Figure 2** An independent prognostic analysis and subgroup analysis according to clinical features. (A-D) Univariate (A, C) and multivariate Cox regression analysis (B, D) of the risk score and other clinical features. (E-J) Clinical subgroup analysis in the training cohort.


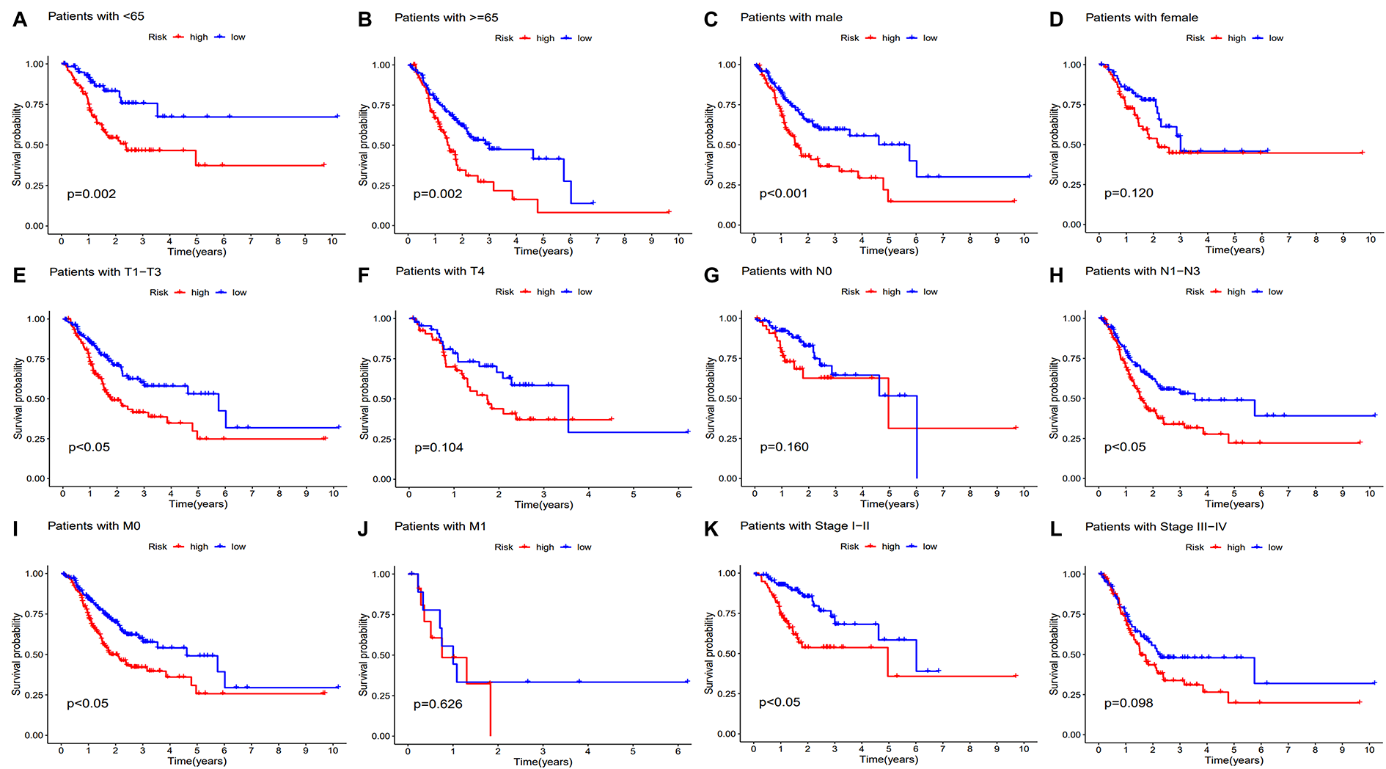


**Supplementary Figure 3.** Variations in survival rates among different clinical subgroups. (A, B) Gender, (C, D) Age, (E, F) T stage, (G, H) N stage, (I, J) M stage, (K, L) Clinical stage.


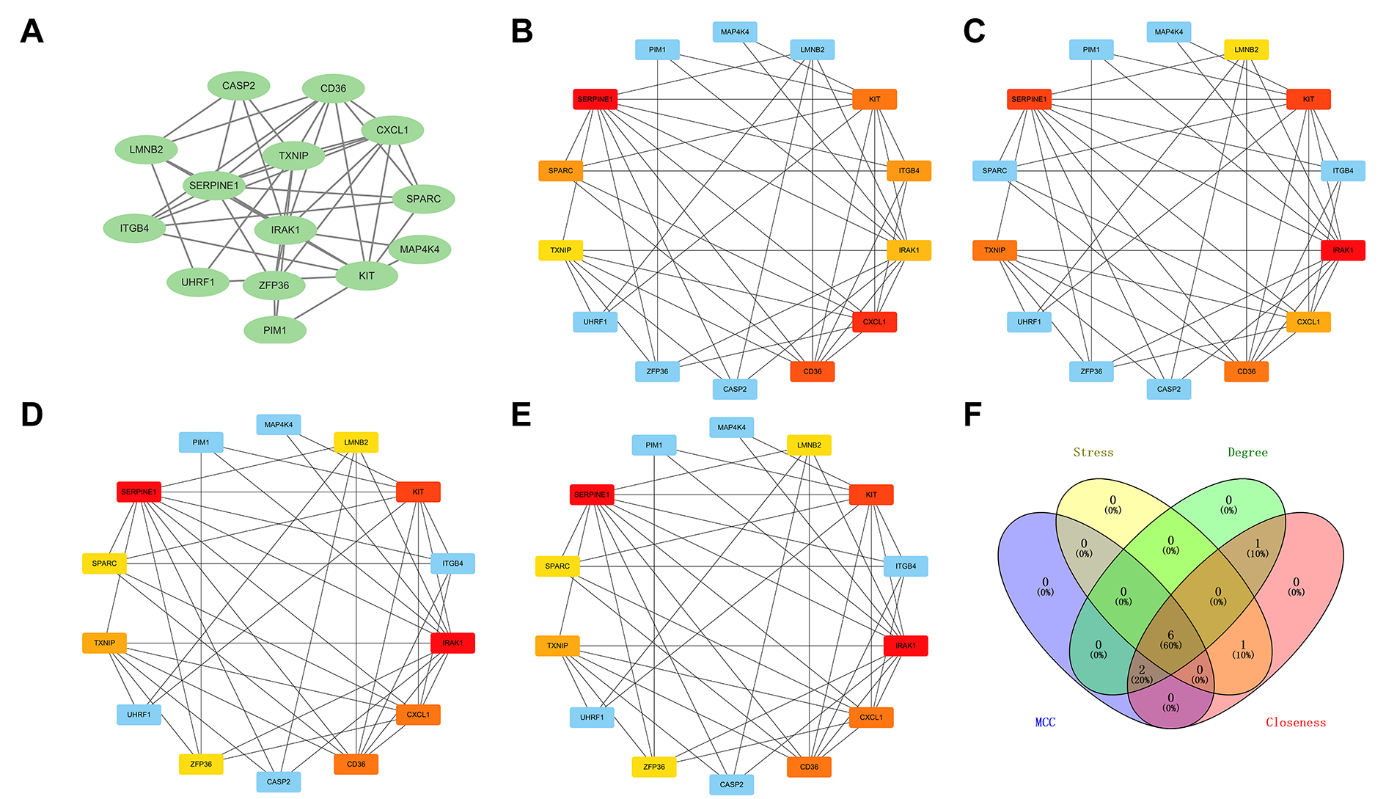


**Supplementary Figure 4** The construction of PPI network. (A) The interaction of model genes. (B-E) Hub genes screened by MCC, Stress, Degree and Closeness algorithms. (F) Venn diagram showing intersected genes of the four algorithms.


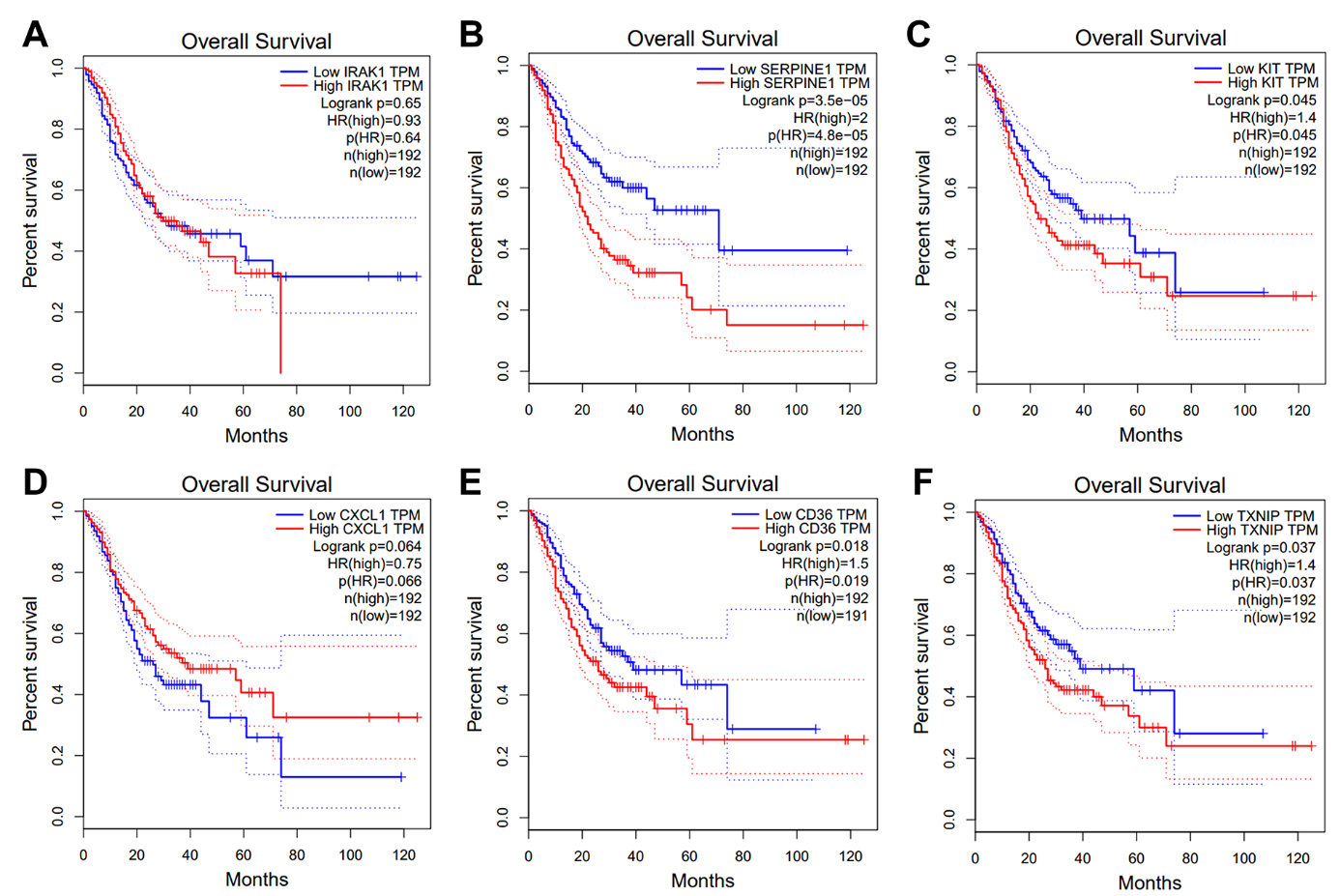


**Supplementary Figure 5.** KM survival analysis of IRAK1 (A), SERPINE1 (B), KIT (C), CXCL1 (D), CD36 (E), TXNIP (F).


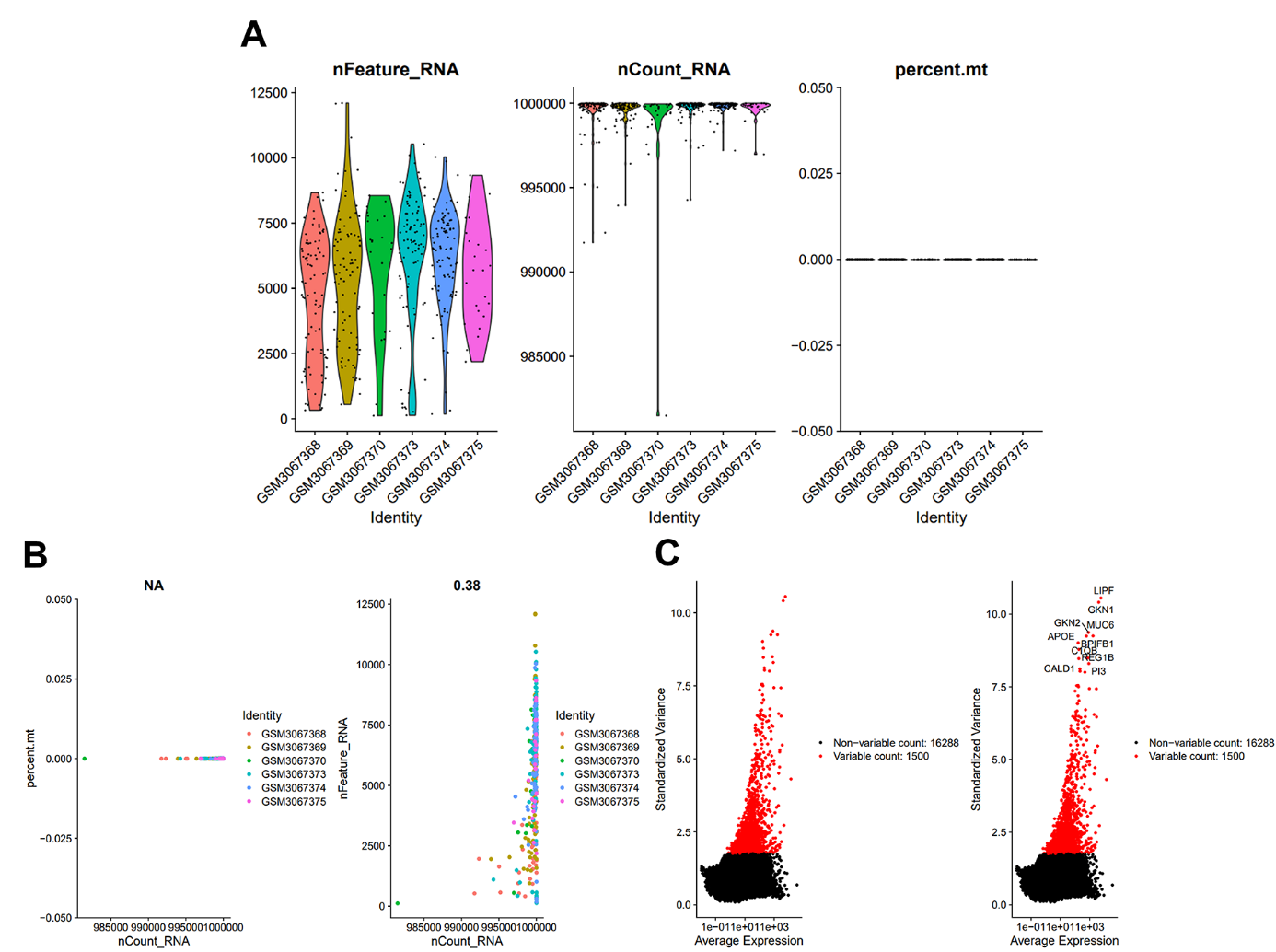


**Supplementary Figure 6.** Filtering and quality control of single-cell data. (A) Screening for genes that meet the eligibility criteria. (B) The correlation of sequencing depth with mitochondrial content and gene number. (C) The genes exhibiting a high coefficient of variation across cells were isolated for further analysis.

**
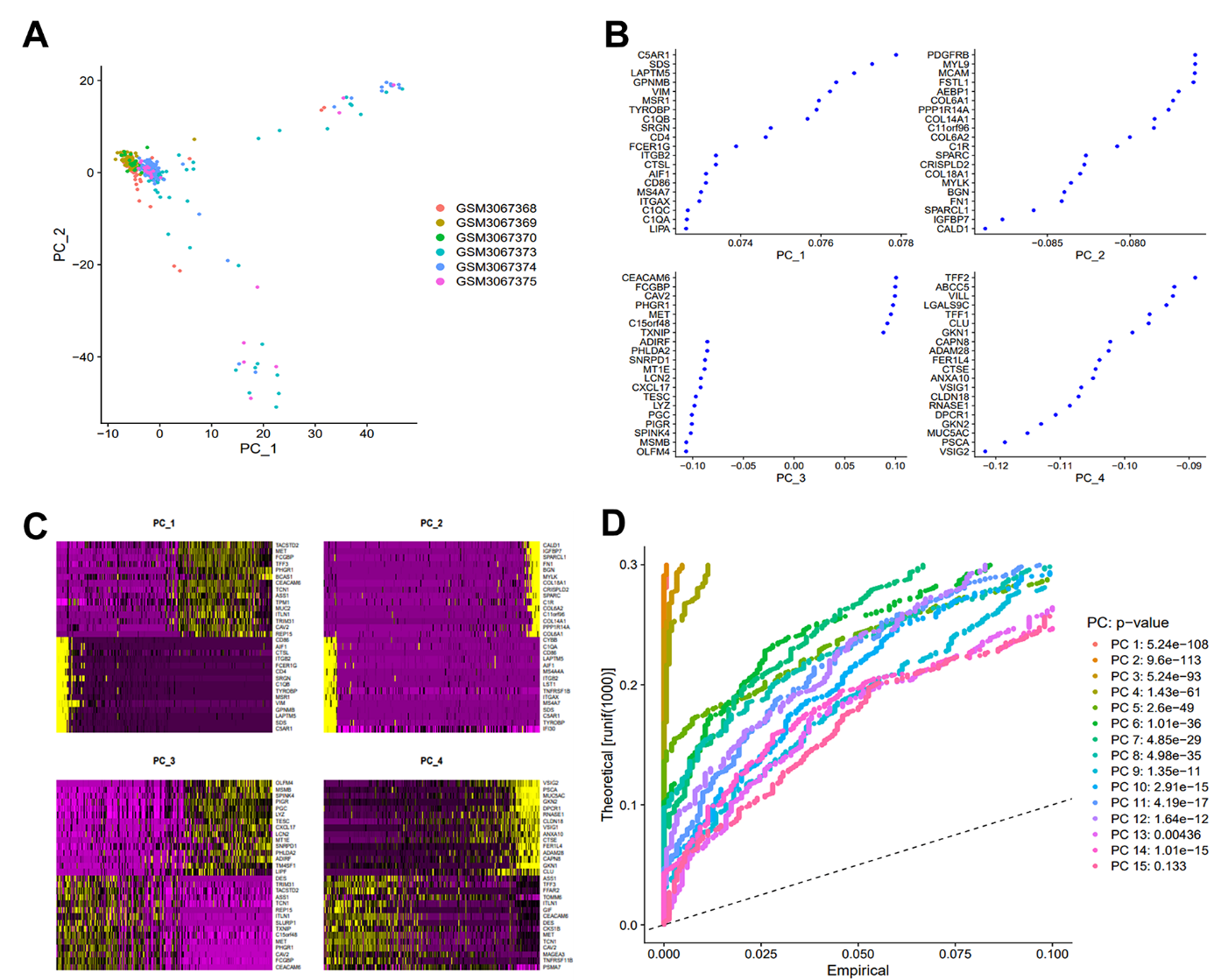
**

**Supplementary Figure 7.** PCA dimension reduction analysis. (A) PCA dimension reduction. (B) The genes characteristic of the initial four PCA components were visually represented. (C) Heat maps of characteristic genes. (D) P-values of PCA components.


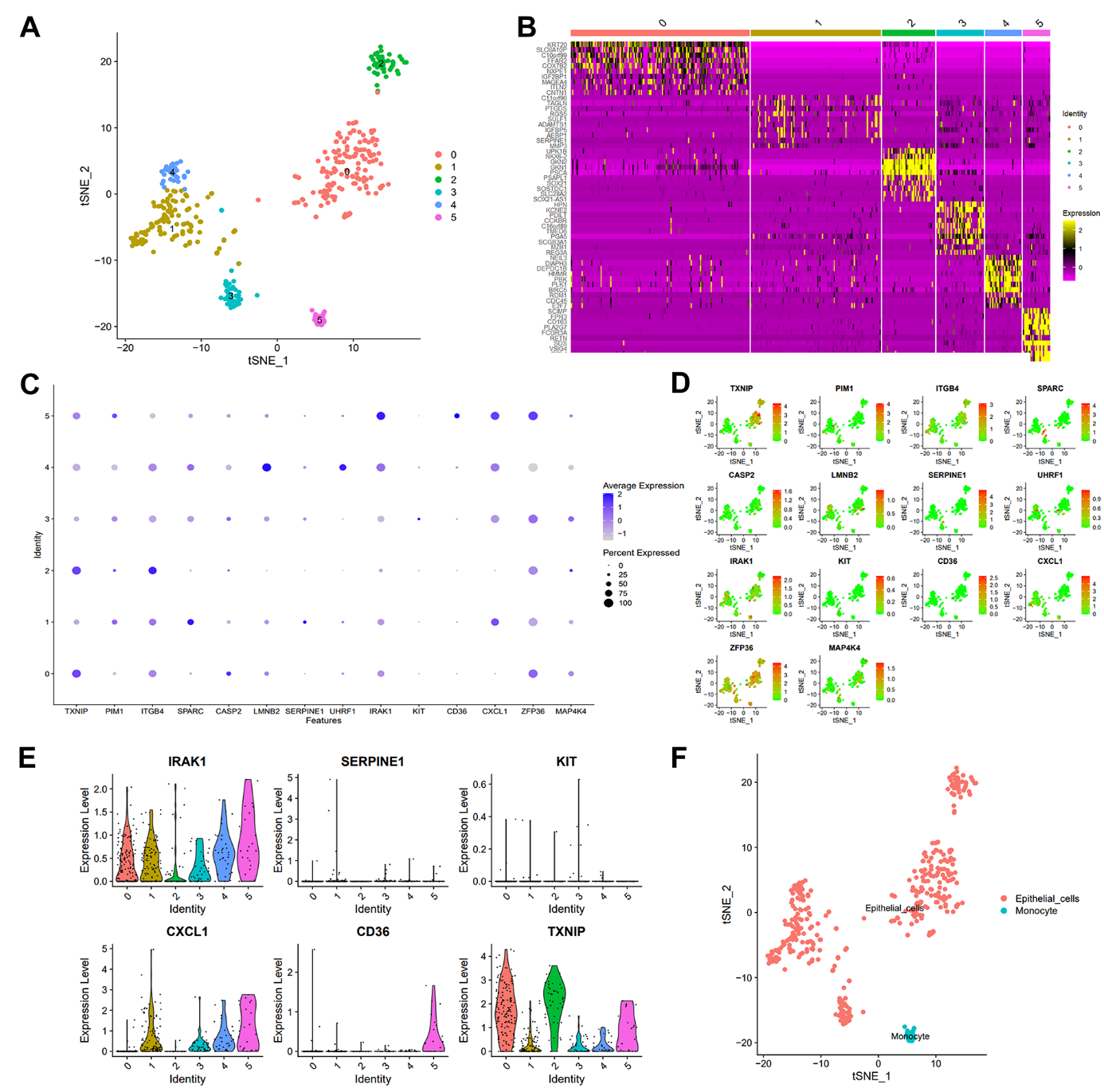


**Supplementary Figure 8** Visualization of single-cell RNA-sequencing analysis. (A) Cell clustering was performed using TSNE method. (B) Heat map of clustering differential genes. (C) Bubble diagram of model genes. (D) Scatter plot visualized the distribution of model genes (E) The expression of genes in each cluster was exhibited by violin diagram (F) Cell-type annotations of the six clusters.
